# Use of traditional Chinese medicine for the treatment and prevention of COVID-19 and rehabilitation of COVID-19 patients: An evidence mapping study

**DOI:** 10.3389/fphar.2023.1069879

**Published:** 2023-01-19

**Authors:** Yanfei Li, Yu Qin, Nan Chen, Long Ge, Qi Wang, Taslim Aboudou, Jiani Han, Liangying Hou, Liujiao Cao, Rui Li, Meixuan Li, Ningning Mi, Peng Xie, Siqing Wu, Linmin Hu, Xiuxia Li, Zhongyang Song, Jing Ji, Zhiming Zhang, Kehu Yang

**Affiliations:** ^1^ Evidence Based Medicine Center, School of Basic Medical Sciences, Lanzhou University, Lanzhou, China; ^2^ Key Laboratory of Evidence Based Medicine and Knowledge Translation of Gansu Province, Lanzhou, China; ^3^ WHO Collaborating Centre for Guideline Implementation and Knowledge Translation, Lanzhou University, Lanzhou, China; ^4^ Chinese GRADE Centre, Lanzhou University, Lanzhou, China; ^5^ Research and education department, Shaanxi Provincial Rehabilitation Hospital, Xi’an, China; ^6^ Department of Social Medicine and Health Management, School of Public Health, Lanzhou University, Lanzhou, China; ^7^ Evidence-Based Social Science Research Centre, School of Public Health, Lanzhou University, Lanzhou, China; ^8^ Department of Health Research Methods, Evidence and Impact, Faculty of Health Sciences, McMaster University, Hamilton, ON, Canada; ^9^ The First Hospital of Lanzhou University, Lanzhou University, Lanzhou, China; ^10^ West China School of Nursing/West China Hospital, Sichuan University, Chengdu, China; ^11^ National Health Commission of the People’s Republic of China, Beijing, China; ^12^ The Seventh Affiliated Hospital of Sun Yat-sen University, Shenzhen, China; ^13^ School of Medicine, Shenzhen Campus of Sun Yat-sen University, Shenzhen, China; ^14^ Affiliated Hospital of Gansu University of Traditional Chinese Medicine, Lanzhou, China; ^15^ Department of Rehabilitation, Gansu Provincial Hospital of Traditional Chinese Medicine, Lanzhou, China

**Keywords:** COVID-19, evidence mapping, gap maps, prevention, rehabilitation, traditional Chinese medicine, treatment

## Abstract

**Background:** The potential effectiveness of traditional Chinese medicine (TCM) against “epidemic diseases” has highlighted the knowledge gaps associated with TCM in COVID-19 management. This study aimed to map the matrix for rigorously assessing, organizing, and presenting evidence relevant to TCM in COVID-19 management.

**Methods:** In this study, we used the methodology of evidence mapping (EM). Nine electronic databases, the WHO International Clinical Trials Registry Platform (ICTRP) Search Portal, ClinicalTrials.gov, gray literature, reference lists of articles, and relevant Chinese conference proceedings, were searched for articles published until 23 March 2022. The EndNote X9, Rayyan, EPPI, and R software were used for data entry and management.

**Results:** In all, 126 studies, including 76 randomized controlled trials (RCTs) and 50 systematic reviews (SRs), met our inclusion criteria. Of these, only nine studies (7.14%) were designated as high quality: four RCTs were assessed as “low risk of bias” and five SRs as “high quality.” Based on the research objectives of these studies, the included studies were classified into treatment (53 RCTs and 50 SRs, 81.75%), rehabilitation (20 RCTs, 15.87%), and prevention (3 RCTs, 2.38%) groups. A total of 76 RCTs included 59 intervention categories and 57 efficacy outcomes. All relevant trials consistently demonstrated that TCM significantly improved 22 outcomes (i.e., consistent positive outcomes) without significantly affecting four (i.e., consistent negative outcomes). Further, 50 SRs included nine intervention categories and 27 efficacy outcomes, two of which reported consistent positive outcomes and two reported consistent negative outcomes. Moreover, 45 RCTs and 38 SRs investigated adverse events; 39 RCTs and 30 SRs showed no serious adverse events or significant differences between groups.

**Conclusion:** This study provides evidence matrix mapping of TCM against COVID-19, demonstrating the potential efficacy and safety of TCM in the treatment and prevention of COVID-19 and rehabilitation of COVID-19 patients, and also addresses evidence gaps. Given the limited number and poor quality of available studies and potential concerns regarding the applicability of the current clinical evaluation standards to TCM, the effect of specific interventions on individual outcomes needs further evaluation.

## Introduction

Since December 2019, multiple cases of pneumonia resulting from unknown causes and having a history of exposure to the South China Seafood Market were reported in Wuhan, Hubei Province (Pan et al., 2020). The causative agent for these pneumonia cases was subsequently identified as the novel coronavirus (SARS-Cov-2). On 11 February 2020, the WHO announced the outbreak of coronavirus disease (hereafter COVID-19) and classified it as a pandemic on 11 March 2020 (WHO, 2020). As of 13 October 2022, over 620 million cases and more than six million deaths resulting from COVID-19 have been reported worldwide (WHO, 2022a). While vaccines and drugs have substantially lowered the number of cases and hospitalizations in several high-income countries, they are largely unaffordable in several parts of the world. Furthermore, the efficacy of current vaccines and the duration of protection remain uncertain (Au and Cheung, 2022; Plata, 2022). In addition, the existing measures against emerging SARS-CoV-2 variants are insufficient and the prognosis and recovery of COVID-19 patients are poor (Au and Cheung, 2022; Huang et al., 2022; Saul et al., 2022; Update to living WHO guideline on drugs for covid-19, 2022).

Many biomolecules extracted from various natural products can prevent and treat a wide spectrum of diseases (Xie et al., 2021). Examples include glycyrrhizin for managing viral hepatitis, ellagic acid to control fibrosis, and phyllanthin for managing hepatitis B (Negi et al., 2008). Vitamin C and Vitamin E have been shown to have a potential effect on metabolic parameters while treating type 1 diabetes (Al Shamsi et al., 2004; Al-Shamsi et al., 2006a; Al-Shamsi et al., 2006b; Guo et al., 2021; Liao et al., 2022). Traditional Chinese medicine (TCM) is based on organic wholeness and treatment based on syndrome differentiation (Tang et al., 2008; Fu et al., 2021). It has been practiced for over 2,000 years and represents a vast and largely untapped resource for bioactive compounds against common pathological conditions aiding general wellbeing (Tu, 2016; Wang et al., 2018). For instance, artemisinin, derived from *Artemisia annua* L (Asteraceae; *Artemisia carvifolia* Buch.-Ham. ex Roxb), has been an active substance against malaria (Tu, 2016). Berberine is an active component of *Coptis chinensis* Franch (Ranunculaceae; *Coptis chinensis* var. brevisepala W.T.Wang et Hsiao). It is a botanical drug used to relieve diabetes and gastrointestinal disorders (Yin et al., 2012). Further, arsenic trioxide, or “Pi Shuang,” can treat acute promyelocytic leukemia (Wang et al., 2018). Saffron (Iridaceae; Crocus sativus L) and its constituents, especially safranal and crocin, have been studied owing to their effect against cancer cells (Amin et al., 2021). Apart from the ongoing discovery and characterization of active substances, studies have also focused on utilizing TCM compound formulations or miscellaneous natural products for effective adjuvant therapy combined with conventional treatments and their role in pain management and palliative treatment (Nurtay et al., 2021; Rizvi et al., 2022). Recent years have witnessed a rapid increase in the understanding and applications of TCM-derived botanical drugs and formulations in evidence-based therapy. In addition, TCM has played an essential role in disease prevention, treatment, and management (Hao et al., 2015; Huang et al., 2021; Li S et al., 2021; Yao et al., 2021; Lu et al., 2022). During the COVID-19 outbreak, TCM along with modern medical approaches significantly contributed to treating and rehabilitating patients (Ge et al., 2021; Lyu et al., 2021). On 14 March 2022, China released the Protocol for Prevention and Control of COVID-19 (Edition 9), which recommended the integration of TCM and modern medicine. In addition, the WHO Expert Meeting on Evaluation of TCM in the Treatment of COVID-19 conducted on 31 March 2022, concluded that the data on the benefits of TCM in reducing the disease exacerbation rate, time for viral clearance, length of hospital stay, and resolution of clinical symptoms for mild and moderate cases of COVID-19 is promising. Therefore, the WHO member states were encouraged to consider the integrative care model developed and applied in China (WHO, 2022b).

Randomized controlled trials (RCTs) and systematic reviews (SRs) are critical for healthcare decision-making (Djulbegovic and Guyatt, 2017; Tian et al., 2017; Ge et al., 2018). Despite some limitations, RCTs are the gold standard for evaluating the clinical efficacy of intervention strategies and an essential basis for developing SRs (Shamseer et al., 2015; Bothwell et al., 2016). In contrast, SRs are the basis for developing practice guidelines and filling the knowledge gaps (Institute of Medicine Committee on Standards for Developing Trustworthy Clinical Practice Guidlines, 2011; Shamseer et al., 2015). There is a lack of consensus on the integration of TCM with conventional COVID-19 treatment (Lyu et al., 2021). This is primarily because of the absence of any comprehensive analysis of existing evidence. Therefore, it is crucial to systematically analyze the published studies for managing COVID-19 with the help of TCM based on study quality, interventions, outcomes, populations, efficacy, and safety. Evidence mapping (EM) is a comprehensive method that systematically and rapidly collects, evaluates, organizes, and presents existing evidence to clarify the research status and address knowledge gaps. Therefore, it improves research value and reduces waste (Katz et al., 2003; Li et al., 2021a; Lu et al., 2022). This study aimed to develop evidence matrix mapping for rigorously assessing, organizing, and presenting evidence relevant to TCM in COVID-19 management.

## Methods

### Data sources and searches

Nine electronic databases (Campbell Library, Cochrane Library, EMBASE, PubMed, Web of Science, CBM, CNKI, CQVIP, and WanFang Data) were searched for studies published until 23 March 2022. In addition, the WHO International Clinical Trials Registry Platform (ICTRP) Search Portal, ClinicalTrials.gov, gray literature, reference lists of articles, and relevant Chinese conference proceedings were also searched. The search strategy was developed with assistance from a medical information retrieval expert and included the use of free terms in combination with Medical terms (MeSH) (for details, see Additional File Pages 2–8).

### Inclusion and exclusion criteria

RCTs and SRs that evaluated the efficacy and/or safety of TCM against COVID-19 were included in the study. The criteria of SRs were in line with the PRISMA-P protocol, which included articles that specifically stated the methods used to identify studies (i.e., search strategy), strategies for study selection (e.g., eligibility criteria and selection process), and detailed methods of synthesis (Shamseer et al., 2015). The inclusion criteria were as follows: 1) population: suspected, confirmed, or convalescent COVID-19 (treatment and rehabilitation) patient, high-risk population (prevention); 2) intervention: TCM or integrated Chinese and modern medicines 3) comparison: standard care (i.e., standard psychological or routine care without medication); or modern medicine; 4) outcome: no restrictions. The exclusion criteria were as follows: 1) duplicate reports; 2) studies with insufficient information (e.g., Abstracts, Letters, and Comments); 3) studies not published in peer-reviewed journals (e.g., studies only appearing on medRxiv or a similar preprint server).

### Study selection and data extraction

The relevant literature was independently screened, extracted, and cross-checked by two researchers, and any disagreements were resolved *via* discussion or consultation with a third researcher. Missing data were retrieved by contacting the relevant authors. Duplicate articles were removed using EndNote X9 and Rayyan software, and the titles and abstracts of the remaining articles were screened. After removing irrelevant studies, the full texts were further analyzed for final inclusion. The following data were extracted: publication year, first author, country, study design, sample size/number of RCTs, population, setting, intervention, control, outcome, and associated *p*-values.

### Quality assessment

The risk of bias assessment tool (Risk of Bias, RoB) (Higgins et al., 2011) recommended in the Cochrane Handbook (version 5.1.0) was used to assess the risk of bias of the included RCTs. A total of seven items were included, and each item was classified as “yes” (low risk of bias), “no” (high risk of bias), or “unclear” (unclear risk of bias). A trial was categorized as “low risk” when all the seven items were classified as “yes” and “high risk” when one or more items were classified as “no.” Otherwise, a trial was considered an “unclear risk”. A Measurement Tool to Assess Systematic Review 2 (AMSTAR-2) (Shea et al., 2017) was used to assess the methodological quality of SRs. AMSTAR-2 consists of 16 items evaluated as “yes,” “partial yes,” or “no.” The quality assessment process was performed online, and the overall quality of the study (“very low quality,” “low quality,” “moderate quality,” and “high quality”) was automatically generated after the assessment was completed. Two researchers independently conducted the quality assessment, and any conflicts were resolved by discussion with a third researcher.

### Data synthesis and analysis

This study was based on the methodology of Global Evidence Mapping (Katz et al., 2003; Bragge et al., 2011) and Campbell evidence and gap map (White et al., 2020) with some modifications (Yang et al., 2018; Li et al., 2020; Li et al., 2021a; Li et al., 2021b). All authors, including experts on evidence-based medicine, EM, and TCM, thoroughly discussed and approved the framework of this study. The EPPI software, R software, and Microsoft Excel 2019 were used for data entry and management. According to the WHO Family International Classifications (WHO-FICs) (Schippers et al., 2010), Core Outcome Set for Clinical Trials on Coronavirus Disease 2019 (COS-COVID) (Jin et al., 2020), and the included studies, evidence mapping was conducted using an established coding system. A bubble chart was used to present the key features of the evidence, and a coordinate system of the “intervention-outcome” evidence frame was constructed. Each bubble in the frame represented a study, the colors represented different study populations, and the size of the bubble represented the study sample size/number of RCTs. The outcome measures of the studies were plotted on the horizontal axis and the intervention measures and corresponding *p*-values on the vertical axis. Descriptive analyses of interventions, outcomes, adverse events, and evidence gaps were conducted with the help of bubble charts.

## Results

### Study selection

**FIGURE 1:**
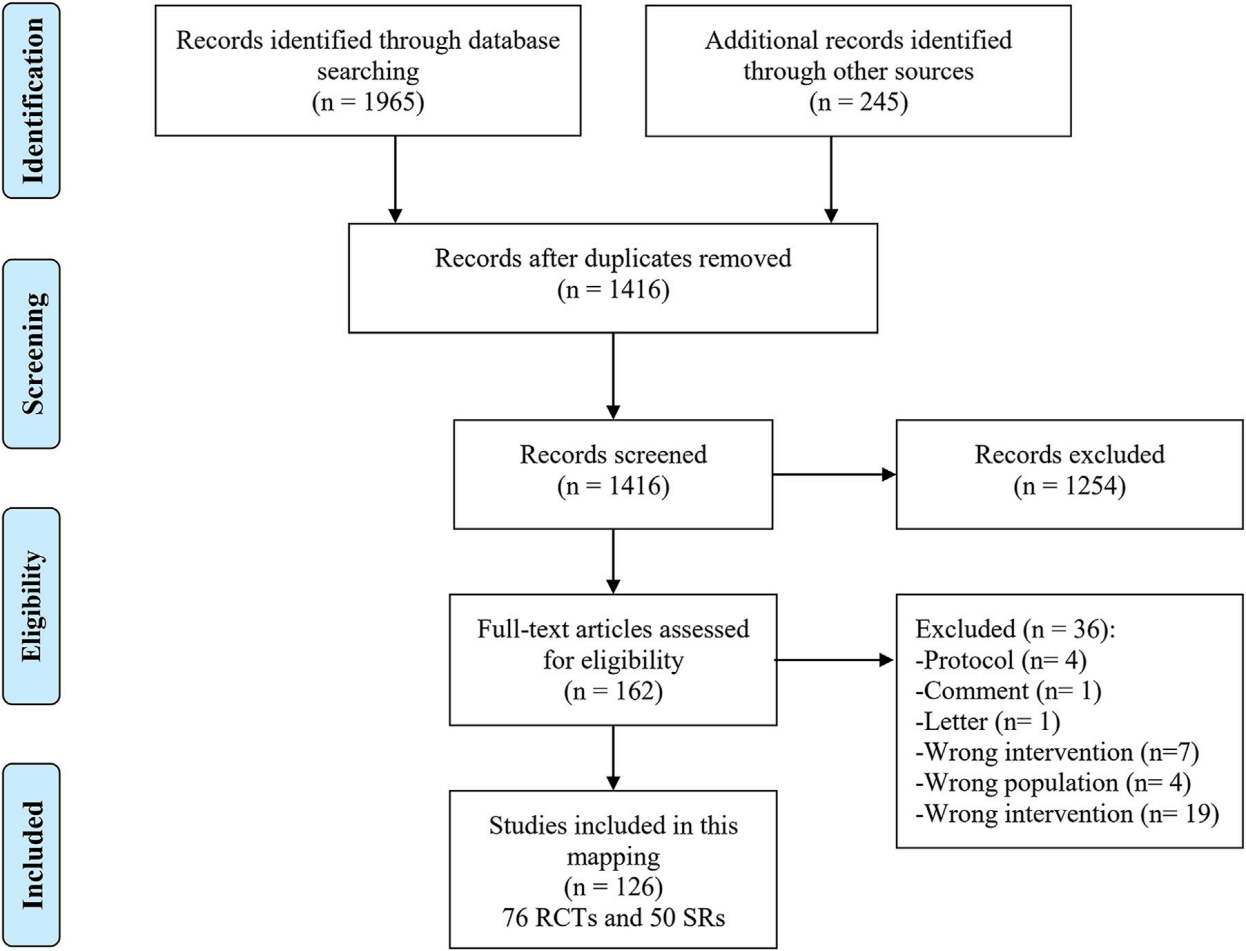
Flow diagram representing the process of literature screening.

As shown in Figure 1, 2210 studies were retrieved through the preliminary screening of databases and supplementary sources, of which 794 were duplicates. In addition, 1,254 studies were excluded after screening titles and abstracts. The full texts of the remaining 162 studies were screened, and 36 unrelated studies were excluded (Additional File Pages 9–10**)**. A total of 126 studies, including 76 RCTs and 50 SRs, were finally included.

### Study characteristics

As shown in Table 1 and Additional File Pages 11–43, 76 RCTs were included, of which 74 were conducted in China. Based on the objectives, the trials were divided into treatment (53, 69.74%), rehabilitation (20, 26.32%), and prevention (3, 3.95%), with 59 intervention categories. The primary intervention was Xuebijing injection (XBJ) (4, 5.26%), and the treatments were provided in a hospital setting (58, 76.32%). In all, 57 common efficacy outcomes and eight study population groups were reported in the relevant trials. The main outcome was the total effective rate (24, 31.58%), and the most prevalent population was that of non-severe COVID-19 patients (28, 36.84%). Adverse events were analyzed in 45 trials (59.21%). Further, 39 trials reported a lack of any serious adverse events or significant differences between the two groups.

**TABLE 1:** Essential characteristics of the included RCTs.

Category	Characteristic	Number	Percentage (*n* = 76)
First author’s country	China	74	97.37%
	Iran	2	2.63%
Health strategy	Prevention	3	3.95%
	Treatment	53	69.74%
	Rehabilitation	20	26.32%
	Health Promotion	0	0
	Palliative/Supportive	0	0
Intervention category^a^	XBJ	4	5.26%
	LHQW	3	3.95%
	HSBD	3	3.95%
	CHD (NS)	3	3.95%
	RDN	2	2.63%
	Others (including 54 categories)	60	78.95%
Setting	Outpatient clinic	3	3.95%
	Hospitalization	58	76.32%
	Day Care Center	0	0
	Home/Community	2	2.63%
	Workplace	0	0
	Remote intervention	0	0
	Others	4	5.26%
	NR	9	11.84%
Outcome (efficacy)	Total Effective Rate	24	31.58%
	Chest CT manifestations	24	31.58%
	Time to fever recovery	20	26.32%
	TCM symptom scores	19	25%
	Rate of cough recovery	18	23.68%
	Others	NA	NA
Population	Suspected COVID-19	3	3.95%
	Non-severe COVID-19	28	36.84%
	Severe COVID-19	4	5.26%
	Critical COVID-19	0	0
	Convalescent COVID-19	5	6.58%
	Confirmed or suspected COVID-19	4	5.26%
	COVID-19 (Mix)	7	9.21%
	COVID-19 (NR)	22	28.95%
	Non-COVID-19 (i.e., high-risk)	3	3.95%
Adverse event	No serious adverse events	24	31.58%
	No significant difference betweenthe two groups (*p* > 0.05)	15	19.74%
	The treatment group had ahigher incidence (*p* < 0.05)	3	3.95%
	The control group had ahigher incidence (*p* < 0.05)	3	3.95%
	NR	31	40.79%

^a^ See Additional File Pages 11–43 for details; NR, not reported; NS, not specified; NA, not applicable.

As shown in Table 2 and Additional File Pages 44–67, 50 SRs were included in this study, of which 47 (94%) were conducted in China. All SRs were therapeutic with nine intervention categories, and TCM (not specified/NS) was the most prevalent treatment method (29, 58%). The primary setting of the interventions was hospitalization (22, 44%). A total of 27 common efficacy outcomes and five study population groups were reported. The main outcome indicator and study population were chest CT manifestations (36, 72%) and COVID-19 patients (not reported) (34, 68%), respectively. Moreover, 38 studies (76%) analyzed adverse events, and 30 reported a lack of any serious adverse events or significant differences between the two groups.

**TABLE 2:** Essential characteristics of the included SRs.

Category	Characteristic	Number	Percentage (*n* = 50)
First author’s country	China	47	94%
	United Kingdom	1	2%
	Korea	1	2%
	India	1	2%
Health strategy	Prevention	0	0
	Treatment	50	100%
	Rehabilitation	0	0
	Health Promotion	0	0
	Palliative/Supportive	0	0
intervention category^a^	TCM (NS)	29	58%
	LHQW	14	28%
	CMI (NS)	1	2%
	QFPD	1	2%
	Honeysuckle	1	2%
	Others (including 4 categories)	4	8%
Setting	Outpatient clinic	0	0
	Hospitalization	22	44%
	Day Care Center	0	0
	Home/Community	0	0
	Workplace	0	0
	Remote intervention	0	0
	NR	28	56%
Outcome (efficacy)	Chest CT manifestations	36	72%
	Rate of cough recovery	32	64%
	Total Effective Rate	31	62%
	Rate of disease aggravation	31	62%
	Rate of fever recovery	31	62%
	Others	NA	NA
Population	Suspected COVID-19	1	2%
	Non-severe COVID-19	10	20%
	Severe COVID-19	0	0
	Critical COVID-19	0	0
	Convalescent COVID-19	0	0
	Confirmed or suspected COVID-19	3	6%
	COVID-19 (Mix)	2	4%
	COVID-19 (NR)	34	68%
	Non-COVID-19 (i.e., high-risk)	0	0
Adverse event	No serious adverse events	5	10%
	No significant difference between the two groups (*p* > 0.05)	25	50%
	The treatment group had a higher incidence (*p* < 0.05)	0	0
	The control group had a higher incidence (*p* < 0.05)	3	6%
	Unclear	5	10%
	NR	12	24%

^a^ See Additional File Pages 44–67 for details; NR, not reported; NS, not specified; NA, not applicable.

### Quality assessment

**FIGURE 2:**
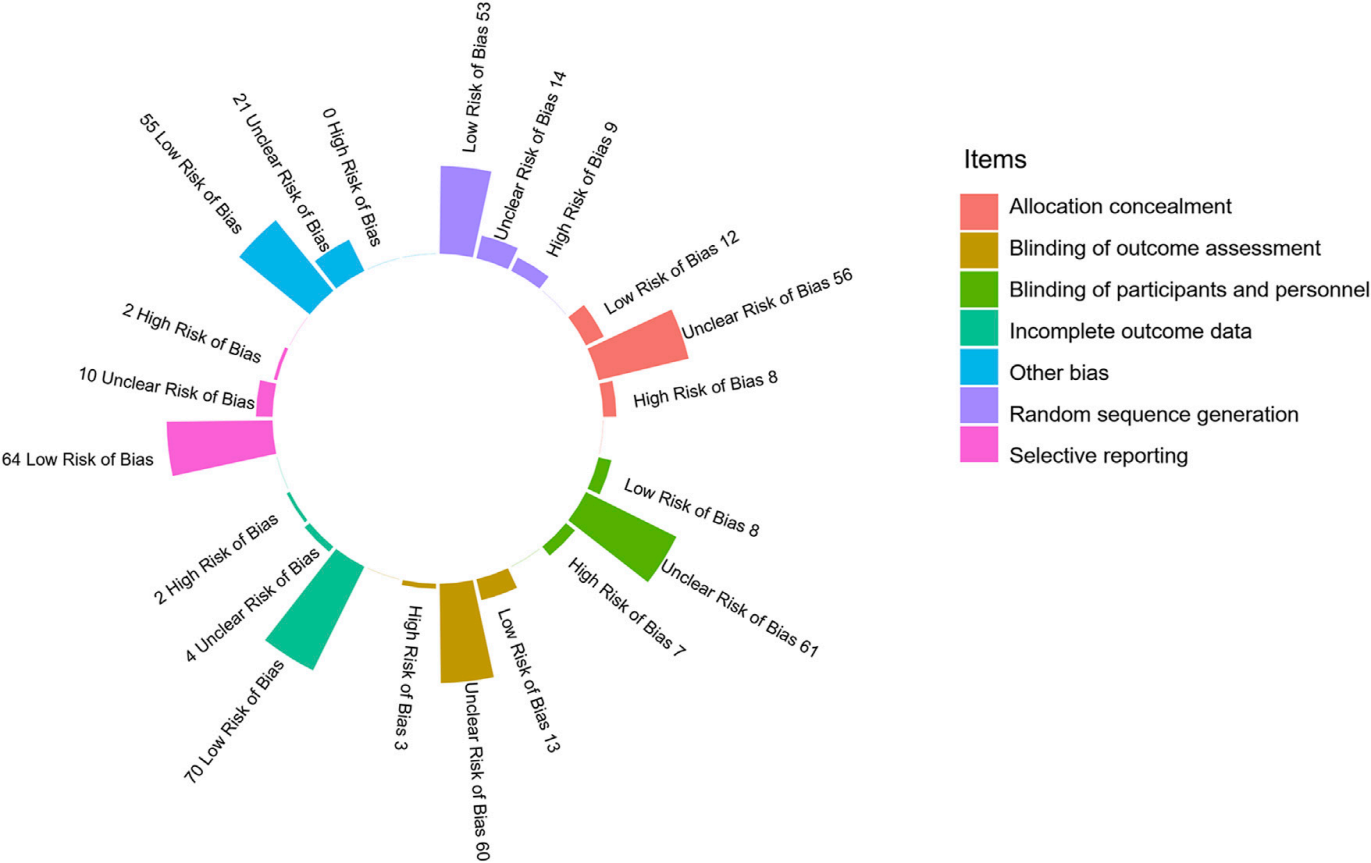
Risk of bias assessment of the included 76 randomized controlled trials.

Among the 76 RCTs, 22 (28.95%) were categorized as high risk of bias, 50 (65.79%) as unclear risk of bias, and 4 (5.26%) as low risk of bias. As shown in Figure 2 and Additional File Pages 68–70, 53 (69.74%) trials described appropriate random sequence generation processes, and 56 (73.68%) performed appropriate allocation concealment methods. Only eight (10.53%) trials were blinded for participants and personnel, and 13 (17.11%) were completed with the outcome assessors blinded to grouping. More than 90% (70/76) of the trials had a low risk of bias in the incomplete outcome data, and two (2.63%) were selective in their data reporting. No other bias could be confirmed in any of the trials.

**FIGURE 3:**
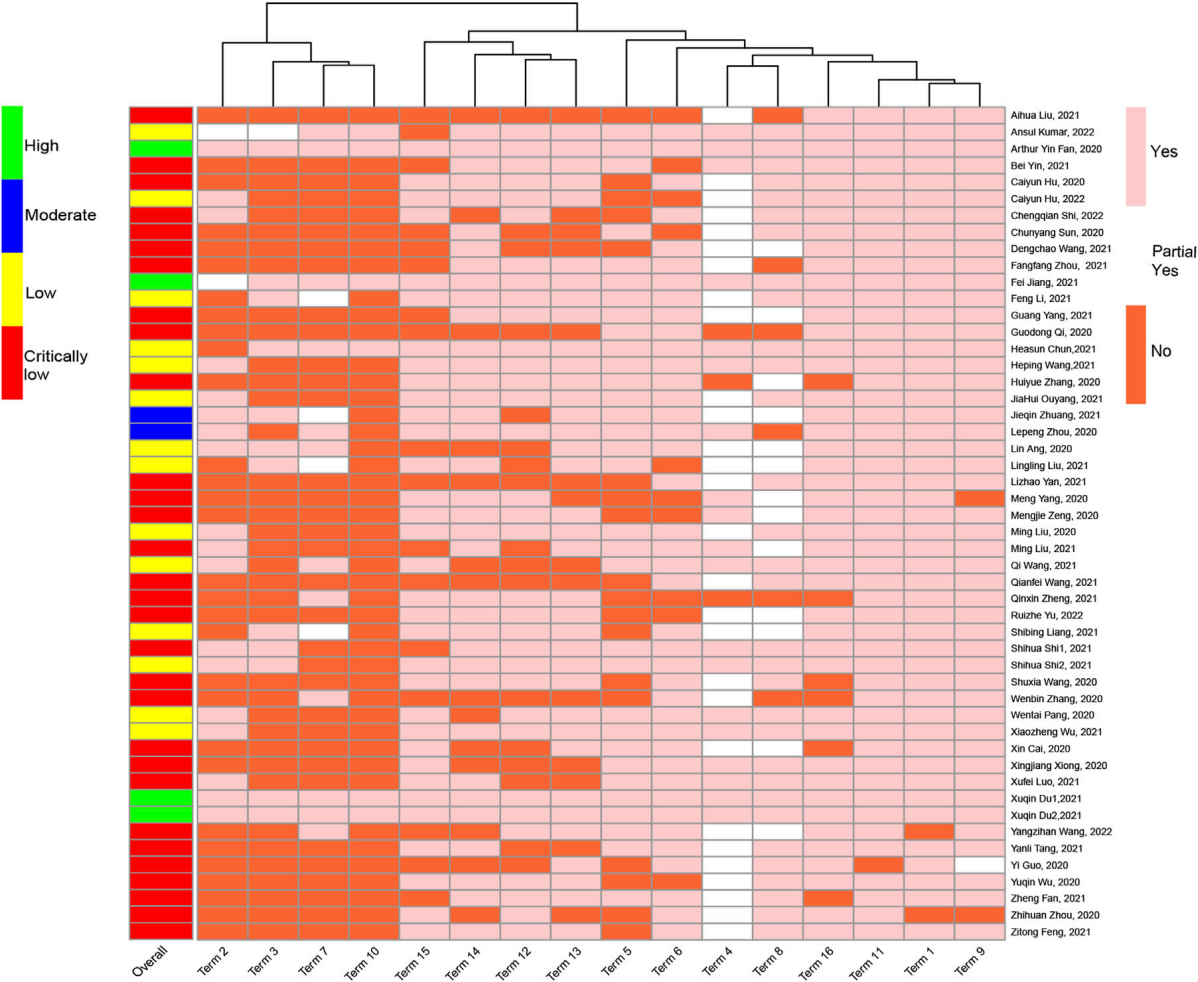
Methodological quality assessment of the included 50 systematic reviews.

Among the 50 SRs, 30 (60%) were characterized as very low quality, 14 (28%) as low quality, two (4%) as moderate quality, and only four (8%) as high quality. In addition, items 11, 1, 9, and 16 were well reported; particularly item 11 (using appropriate statistical combination method), which was fully reported in 49 (98%) SRs, was well reported. In addition, items 2, 3, 7, and 10 had significantly lower reporting rates, particularly item 10 (reporting study funding source), which was only reported in six SRs (12%) (Figure 3, see Additional File Pages 71–73 for details).

### Mapping

As already mentioned, the studies were classified based on their objectives into treatment, rehabilitation, and prevention. To compare the characteristics of the different interventions and outcomes, we also mapped the studies based on sample size/number of RCTs, population, intervention, outcome, and corresponding *p*-values (see Additional File Pages 11–67 for data sources).

#### Treatment

**FIGURE 4:**
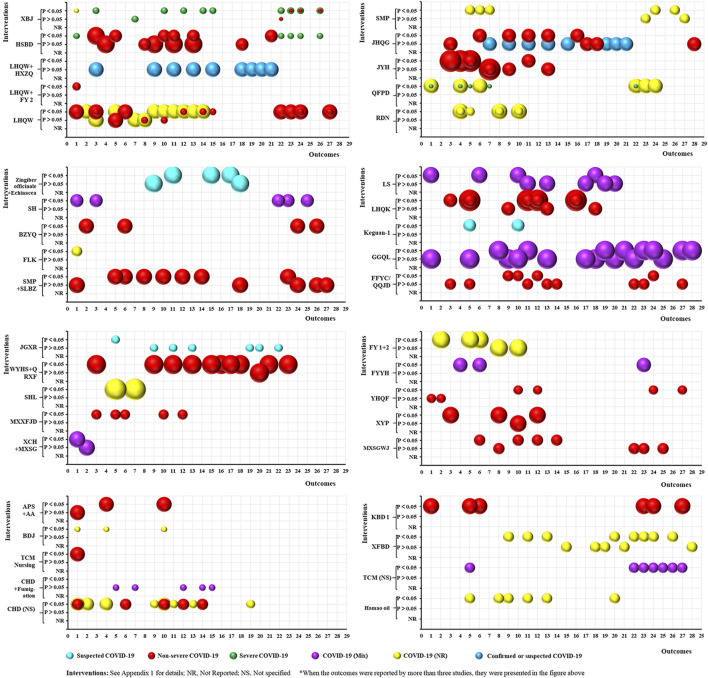
Evidence mapping of traditional Chinese medicine (TCM) interventions in coronavirus disease (COVID-19) treatment RCTs. Outcome codes: Total Effective Rate (1); Clinical cure rate (2); Disease aggravation rate (3); Duration of hospitalization (4); Chest CT manifestations (5); TCM syndrome score (6); Viral nucleic acid negative conversion rate (7); Time to viral assay conversion (8); Fever recovery rate (9); Time to fever recovery (10); Cough recovery rate (11); Time to cough recovery (12); Fatigue recovery rate (13); Time to fatigue recovery (14); Rate of shortness of breath recovery (15); Sputum disappearance rate (16); Rate of muscle pain disappearance (17); Rate of sore throat disappearance (18); Diarrhea disappearance rate (19); Anorexia disappearance rate (20); Rate of chest tightness disappearance (21); Inflammatory biomarkers: WBC (22), LYM (23), CRP (24), NEU (25), ESR (26), and PCT (27); and Nausea/vomiting disappearance rate (28).

As shown in Figure 4, 53 RCTs focused on treating COVID-19 using TCM. The studies included 39 intervention categories, 28 common efficacy outcomes (excluding the outcomes reported by less than three trials), and the following study populations: 1) suspected COVID-19: 3, 5.66%; 2) non-severe COVID-19: 23, 43.40%; 3) severe COVID-19: 4, 7.55%; 4) COVID-19 (Mix): 7, 13.21%; 5) COVID-19 (NR): 14, 26.42%; and 6) confirmed or suspected COVID-19: 2, 3.77%. Based on the mapping, XBJ (4, 7.55%) was the most commonly used intervention, and chest CT manifestations (22, 41.51%), time to fever recovery (20, 37.74%), total effective rate (19, 35.85%), rate of cough recovery (18, 33.96%), and rate of fatigue recovery (16, 30.19%) were the most common outcomes. Furthermore, all relevant trials consistently demonstrated that TCM significantly improved TCM syndrome scores and rate of sputum disappearance (i.e., consistent positive outcomes) (*p* < 0.05). However, the statistical effect of TCM was still contradictory for the remaining 26 outcomes (i.e., inconsistent outcomes).

**FIGURE 5:**
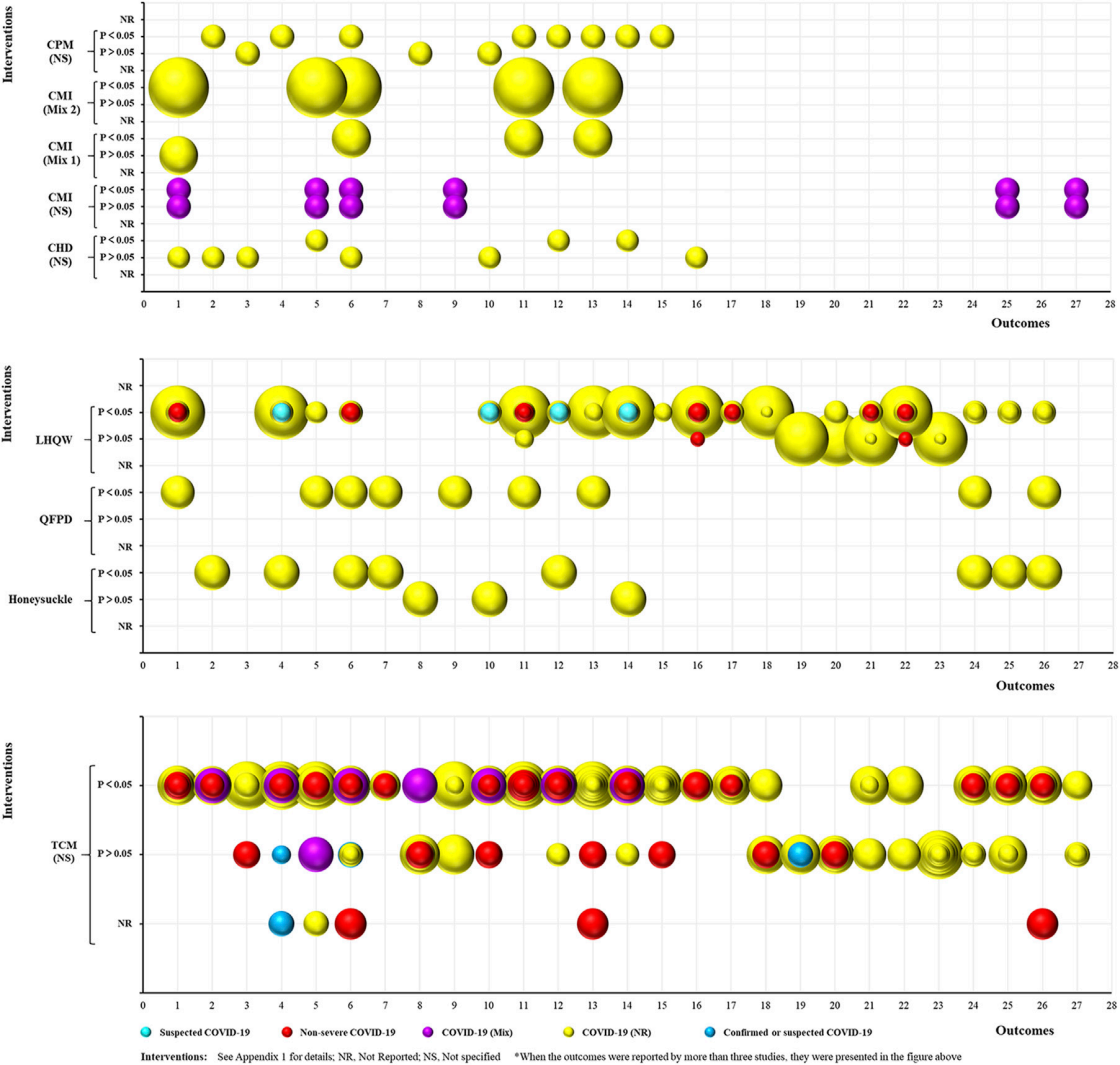
Evidence mapping of TCM intervention in COVID-19 treatment SRs. Outcome codes: Total Effective Rate (1); Clinical cure rate (2); Mortality rate (3); Disease aggravation rate (4); Duration of hospitalization (5); Chest CT manifestations (6); TCM syndrome score (7); Viral nucleic acid negative conversion rate (8); Time to viral assay conversion (9); Fever recovery rate (10); Time to fever recovery (11); Cough recovery rate (12); Time to cough recovery (13); Fatigue recovery rate (14); Time to fatigue recovery (15); Rate of shortness of breath recovery (16); Sputum disappearance rate (17); Rate of muscle pain disappearance (18); Rate of sore throat disappearance (19); Diarrhea disappearance rate (20); Anorexia disappearance rate (21); Rate of chest tightness disappearance (22); Nausea/vomiting disappearance rate (23);and Inflammatory biomarkers: WBC (24), LYM (25), CRP (26), and IL-6 (27).

In all, 50 SRs focused on the treatment of COVID-19 using TCM (Figure 5), and included nine intervention categories, 27 common efficacy outcomes (excluding the outcomes reported by less than three SRs), and the following study populations: 1) suspected COVID-19: 1, 2%; 2) non-severe COVID-19: 10, 20%; 3) confirmed or suspected COVID-19: 3, 6%; 4) COVID-19 (Mix): 2, 4%; and 5) COVID-19 (NR): 34, 68%. The most common interventions were NS TCM (29, 58%) and LHQW (14, 28%). Chest CT manifestations (36, 72%), rate of cough recovery (32, 64%), total effective rate (31, 62%), rate of disease aggravation (31, 62%), and rate of fever recovery (31, 62%) were the most common outcomes. Moreover, two outcomes (TCM syndrome scores and rate of sputum disappearance) were consistently positive (*p* < 0.05), two (rate of sore throat disappearance and nausea/vomiting disappearance) were consistently negative (*p* > 0.05), and the remaining 23 were inconsistent.

#### Rehabilitation

**FIGURE 6:**
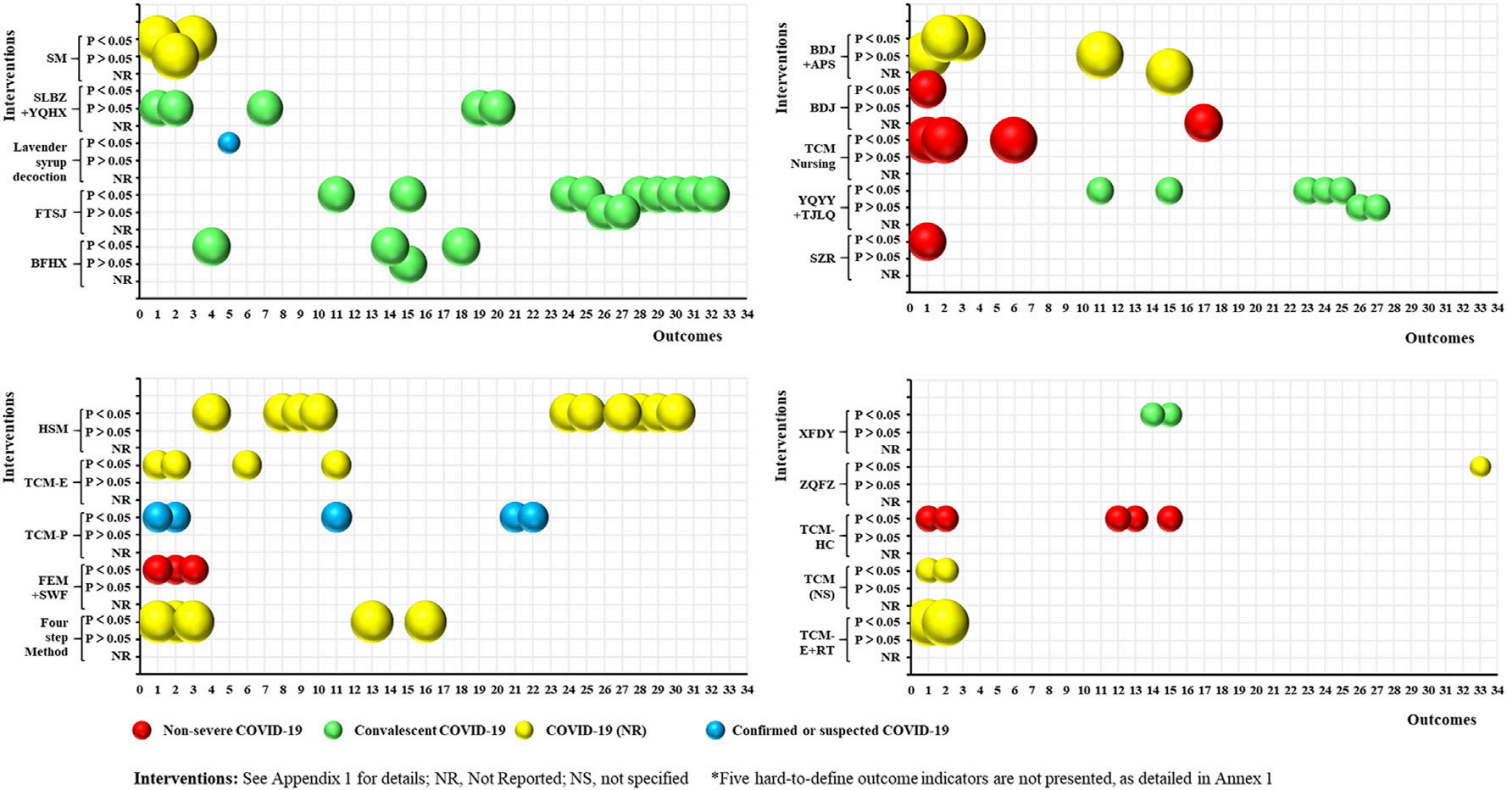
Evidence mapping of TCM interventions in COVID-19 rehabilitation RCTs. Outcome codes: Anxiety score (1); Depression score (2); Sleep quality scale (3); 6-Min Walk Distance (4); Olfactory dysfunction (5); Patient satisfaction (6); QOL (7); Skeletal muscle index (8); Grip strength (9); Balance function (10); Total effective rate (11); Disease aggravation rate (12); Duration of hospitalization (13); Chest CT manifestations (14); TCM syndrome score (15); Time to viral assay conversion (16); Fatigue recovery rate (17); Fatigue Assessment Inventory score(18); Recurrence of SARS-CoV-2 viral RNA positive (19); Abnormality rate of chest CT manifestations (20); Stress scores (21); The TCM Five Emotions (22); Peak expiratory flow (23); and Immune Function Indexes: CD3 (24), CD4 (25), CD8 (26), CD4/CD8 (27), IgA (28), IgG (29), IgM (30), C3 (31), C4 (32), and Immune Function (33).

As shown in Figure 6, 20 RCTs focused on the rehabilitation of COVID-19 patients using TCM. This included 20 intervention categories, 33 common efficacy outcomes (excluding the five hard-to-define outcomes), and four study population groups (non-severe COVID-19: 5, 25%; convalescent COVID-19: 5, 25%; COVID-19 (NR): 8, 40%; and confirmed or suspected COVID-19: 2, 10%). The anxiety (13, 65%) and depression (11, 55%) scores were the most common outcomes reported. Furthermore, 23 outcomes were consistently positive (*p* < 0.05), four (QoL, recurrence of SARS-CoV-2 viral RNA positivity, abnormality rate of chest CT manifestations, and CD8 levels) were consistently negative (*p* > 0.05), and five (anxiety score, depression score, total effective rate, TCM syndrome score, and CD4/CD8) were inconsistent. The statistical effect of TCM was unclear for the remaining outcomes. However, no SRs related to the rehabilitation of COVID-19 patients were identified.

#### Prevention

Three RCTs focused on preventing COVID-19 *via* TCM interventions. Interventions included heat-sensitive Moxibustion combined with acupoint application (Yan X. et al., 2020), Fuzheng Gubiao Fanggan particles (Dan, 2021), and Jinhao Jiere granules combined with Huoxiangzhengqi oral liquids (Yan B. H. et al., 2020). There were three common efficacy outcomes, including the incidence of cold-like symptoms, COVID-19 infection, and improvement in immune function. All outcomes improved significantly in the intervention group compared with the control group in each study (*p* < 0.05). However, no SRs associated with COVID-19 prevention could be identified.

### Adverse events

A total of 45 RCTs and 38 SRs analyzed the adverse events of TCM interventions against COVID-19. No serious adverse events or significant differences were observed between the two groups in 39 RCTs. Three RCTs reported a significantly higher incidence of diarrhea and nausea in the TCM-intervention groups (*p* < 0.05), and three showed that the control group had significantly higher rates of gastrointestinal bleeding, rash, insomnia, tremor, and pruritus (*p* < 0.05). In addition, 30 SRs did not report any serious adverse events or significant differences between the two groups. Three SRs showed that adverse reactions were significantly higher in the control group (*p* < 0.05), and five SRs reported unclear results for adverse events.

## Discussion

### Main findings

In this evidence-mapping study, RCTs and SRs of TCM-based COVID-19 management published before March 2022 were systematically searched. A total of 126 studies were included, of which 90% were from China. The studies focused on three objectives/health strategies, 64 intervention categories, 59 common efficacy outcomes, and eight population groups. Among the 76 RCTs, only four were categorized as having a low risk of bias. The primary health strategy was therapeutic, and non-severe COVID-19 patients comprised the most common population. In addition, XBJ was the most common intervention. Most studies evaluated the effect of a specific intervention on the total effective rate. Among the 50 SRs, only four were assessed as “high quality.” Furthermore, the objective of all the studies included in the SRs was therapeutic, and non-severe COVID-19 patients were the most common population. TCM (NS) was the most applied intervention, and most studies evaluated its effect on chest CT manifestations.

Overall, most of the included studies focused on treating COVID-19 patients. A total of 53 treatment-related RCTs included 39 intervention categories, of which XBJ was the most common intervention, with four published RCTs. Only one RCT was published for each of the 31 other interventions. The high number of intervention categories may be related to the specific characteristics of TCM formulations (Tang et al., 2008; Fu et al., 2021). Compared with modern medicine, TCM emphasizes on the constitution of the patient more than the disease and considers individual differences. Therefore, TCM prescriptions are formulated based on the environment and physical and mental health of the patient. This considerably increases the difficulty of large-sample clinical research for a specific intervention. Therefore, further investigation into designing and conducting relevant studies according to the theories of TCM is warranted (ManKe et al., 2020). Only a few RCTs recruited critical COVID-19 patients. The sample size of severe COVID-19 patients was small, which could be attributed to the characteristics of the SARS-CoV-2 virus (An et al., 2021; Young et al., 2022). Given the efficacy of TCM against the non-severe COVID-19 population, it is reasonable to expect similar outcomes of TCM among severe/critical COVID-19 patient population.

Although most RCTs and SRs evaluated the efficacy of TCM interventions for a wide range of outcomes, the results were inconsistent for approximately 85% of the efficacy outcomes in SRs and 54% of the outcomes in RCTs. High-quality SRs should be conducted to overcome this limitation. Nevertheless, all therapeutic studies that analyzed “TCM syndrome scores” reported significant improvement with the interventions. Therefore, the abovementioned discrepancy could be because of different standards for evaluating the clinical efficacy between TCM and modern medicine (Qinggang, 2018; Yu et al., 2020; Xin et al., 2021). Further studies are needed to evaluate the efficacy of TCM solely depending on the Core Outcome Set and evaluation standards of modern medicine. In addition, most studies reported that TCM formulations were safe for COVID-19 patients (Jiang et al., 2021; Wang et al., 2022). Some common adverse events were diarrhea, nausea, and other minor reactions, which disappeared on their own and were not more frequent in the TCM intervention group compared with those in the control group (Ang et al., 2020; Liu et al., 2020). TCM has also shown promising results in rehabilitating COVID-19 patients (Huang et al., 2022). Most studies showed that TCM intervention could significantly improve the psychological function, respiratory function, lung function, and immune function of patients who had been diagnosed with COVID-19 or discharged from the hospital (Jingling et al., 2021; Li L. et al., 2021; Chen et al., 2022). In addition, TCM reduced the infection rate of COVID-19 and the incidence of cold-like symptoms in individuals at risk of contracting COVID-19 (Yan B. H. et al., 2020; Yan X. et al., 2020; Dan, 2021). However, only a few studies on COVID-19 prevention using TCM are available, which are not enough to draw a definite conclusion.

### Evidence gaps and future directions

The main evidence gaps in this study involved the study quality, study populations, interventions, and outcomes. For instance, more than 28% of the relevant RCTs had a high risk of bias, and more than 80% of the SRs were of low or very low quality. In addition, only four RCTs were conducted in severe COVID-19 patients. No SRs or RCTs were observed including critical COVID-19 cases, and no SRs were found for rehabilitating and preventing COVID-19. The definition of specific interventions in the included studies was unclear, as were the respective efficacies of the different interventions because of a lack of continuous research. More than 50% of the SRs did not specify intervention programs, and 76 RCTs had 59 intervention categories. For studies on prevention and rehabilitation, only one RCT was published for each of the 23 different interventions. There is currently no consensus on the efficacy of some outcomes following TCM, which will require further high-quality RCTs or SRs. Finally, the differences in the evaluation standards for TCM and modern medicine and the Core Outcome Set for TCM, warrant further investigation.

### Strengths and limitations

We systematically searched for RCTs and SRs related to TCM interventions for COVID-19 and compared the specific interventions, outcomes, populations, and study quality. Evidence mapping can help identify the knowledge gaps that provide a reference for researchers. Meanwhile, this study provides an evidence matrix of TCM, which is a scientific and comprehensive reference basis for clinical policymakers in COVID-19 management. Nevertheless, this study has some limitations that need to be considered. First, given that RCTs and SRs are highly representative of a particular research topic, other types of studies, such as cohort studies, and case-control studies, were not included. Second, given the research purpose and evidence-mapping methodology, we focused on the analysis and presentation of evidence rather than quantitative statistics. Third, most of the included studies were conducted in China; thus, the generalizability of the outcomes is limited. Fourth, our results were based only on publications published before March 2022, and results need to be regularly updated as new studies emerge.

## Conclusion

Through the evidence mapping methodology, we finalized an evidence matrix consisting of 64TCM intervention categories and 59 common efficacy outcomes. Although the number and quality of studies are limited, TCM is a promising alternative for the treatment, rehabilitation, and prevention of COVID-19. Most relevant studies showed significant effects of the intervention on various outcomes. However, many outcomes have conflicting results, which may require further clarification through high-quality RCTs or SRs. Furthermore, it remains to be determined whether the existing standards of evaluating the clinical efficacy of modern medicine are also applicable to TCM. In addition, high-quality and large-sample studies, studies including severe or critical COVID-19 cases, and studies related to COVID-19 rehabilitation and prevention are scarce and will have to be explored further.

## Data Availability

The original contributions presented in the study are included in the article/Supplementary Material, further inquiries can be directed to the corresponding authors.

## Glossary

**APS**: Auricular Point Sticking
**BDJ**: Baduanjin exercise
**BFHX**: Bufei Huoxue Capsules
**BZYQ**: Buzhong Yiqi Decoction
**CHD**: Chinese Herbal Decoction
**CMI**: Chinese Medicine Injection
**CRP**: C-reactive protein
**CT**: Computed Tomography
**ESR**: Erythrocyte Sedimentation Rate
**FFYC**: Fufang Yinchai granules
**FLK**: Feilike capsule
**FTSJ**: Futu Shengjin Rehabilitation Formul
**FYYH**: Feiyan Yihao Chinese medicine granules
GGQL: Gegen Qinlian Pills
**HSBD**: Huashi Baidu granules
**HXZQ**: Huoxiang Zhengqi dropping pills
**JGXR**: Jiegeng Xingren Decoction
**JHQG**: Jinhua Qinggan Granules
**JYH**: Jinyinhua Oral Liquid
**LHQK**: Lianhua Qingke tablets
**LHQW**: Lianhua Qingwen granules/capsules
**LS**: Liushen Pill
**LYM**: Lymphocyte
**MXSG**: Maxing Shigan Decoction
**MXSGWJ**: Maxingshigan-Weijing Decoction
**MXXFJD**: Maxing Xuanfei Jiedu Decoction
**NEU**: Neutrophils
**PCT**: Procalcitonin
**QFPD**: Qingfei Paidu Decoction
**QoL**: Quality of life
**QQJD**: Qingqiao Jiedu granule
**QRXF**: Qingre Xuanfei Recipe
**RDN**: Reduning injection
**SH**: Shenhuang granules
**SHL**: Shuanghuanglian oral liquids
**SLBZ**: Shenling Baizhu Powder
**SMP**: Shengmai Powder
**TCM**: Traditional Chinese medicine
**WYHS**: Wenyang Huashi Prescription
**XBJ**: Xuebijing injection
**XCH**: Xiaochaihu Decoction
**XFBD**: Xuanfei Baidu Decoction
**XYP**: Xiyanping Injection
**YHQF**: Yinghuang Qingfei Capsule
