# Supplementary material for: Use of traditional Chinese medicine for the treatment and prevention of COVID-19 and rehabilitation of COVID-19 patients: An evidence mapping study
Source: Front Pharmacol. 2023 Jan 19;14:1069879. doi: 10.3389/fphar.2023.1069879 (PMC9892723; doi:10.3389/fphar.2023.1069879)
Supplement: Supplementary file 1 [file DataSheet1.DOCX]

Supplementary Material

**Contents**

[Table S1 Electronic search strategies 2](#_Toc123829370)

[Campbell Library <March 23, 2022> 0 2](#_Toc123829371)

[Cochrane Library <March 23, 2022> 135 3](#_Toc123829372)

[EMBASE <March 23, 2022> 323 4](#_Toc123829373)

[PubMed <March 23, 2022> 232 5](#_Toc123829375)

[Web of Science <March 23, 2022> 292 6](#_Toc123829377)

[CBM <March 23, 2022> 294 7](#_Toc123829379)

[CNKI <March 23, 2022> 204 7](#_Toc123829381)

[CQVIP <March 23, 2022> 185 7](#_Toc123829383)

[WanFang Data <March 23, 2022> 300 8](#_Toc123829385)

[Clinical trial registry <March 23, 2022> 32 8](#_Toc123829387)

[WHO International Clinical Trials Registry Platform <March 23, 2022> 187 8](#_Toc123829388)

[Gray literature, reference lists of articles and relevant conference <March 23, 2022> 26 8](#_Toc123829389)

[Table S2 Excluded studies: 36 9](#_Toc123829390)

[Table S3. Summary of the included RCTs 11](#_Toc123829391)

[Table S4. Summary of the included SRs 44](#_Toc123829392)

[Table S5. Risk of bias assessment for RCTs 68](#_Toc123829393)

[Table S6. Quality assessment results for SRs (AMSTAR-2) 71](#_Toc123829394)

[Table S7. Traditional Chinese medicine prescriptions (including main components) 74](#_Toc123829395)

# Table S1 Electronic search strategies

| **Databases [Platform]** Searches run March, 2022 | **Results** |
| --- | --- |
| **Campbell Library** | 0 |
| **Cochrane Library** | 135 |
| **EMBASE** | 323 |
| **PubMed** | 232 |
| **Web of Science** | 292 |
| **CBM** | 294 |
| **CNKI** | 204 |
| **CQVIP** | 185 |
| **WanFang Data** | 300 |
| **Other sources (ClinicalTrials.gov, gray literature, and reference lists of articles)** | 245 |
| **TOTAL** | **2210** |
| **Duplicate** | **794** |

**Database: Campbell Library <March 23, 2022> 0**

**Search Strategy:**

| **#** | **Searches** | **Results** |
| --- | --- | --- |
| #1 | "Traditional Chinese Medicine OR Medicine, Chinese Traditional OR Traditional Medicine, Chinese OR Chinese Traditional Medicine OR Chinese Medicine, Traditional OR Drugs, Chinese Herbal OR Chinese Drugs, Plant OR Chinese Herbal Drugs OR Herbal Drugs, Chinese OR Plant Extracts, Chinese OR Chinese Plant Extracts OR Extracts, Chinese Plant OR Chinese medicine formula OR Chinese medicine decoction OR Medicine, Kampo OR “herbal formula” OR “herbal medicine” OR “herbal decoction” OR “herbal preparation” OR Chinese medicine injection OR qinghai pandu Decoction OR haushi Baidu Formula OR xuanwei Baidu Formula OR Chinese patent medicine OR Chinese medicine Granule OR Chinese medicine Capsule OR Jinhua qinglan Granule OR lianhuan qingben Capsule OR lianhuan qingben Granule OR Xuebijing injection OR xifanping injection" **anywhere and** "coronavirus* OR coronovirus* OR coronavirinae* OR "2019‐nCoV" OR 2019ncovr OR “2019‐CoV” OR ncovid19 OR "nCoV‐2019" OR "COVID‐19" OR COVID19 OR "CORVID‐19" OR covid19 OR "WN‐CoV" OR wecov OR "HCoV‐19" OR hcox19 OR CoV OR "2019 novel*" OR Ncov OR "n‐cov" OR "SARS‐CoV‐2" OR "SARSCoV‐2" OR "sarscov" OR "SARS‐CoV2" OR sarscov2 OR "SARS‐Cov19" OR "SARSCov‐19" OR "SARS‐Cov‐19"" **anywhere** **published in** "Campbell Systematic Reviews" | 0 |

**Database: Cochrane Library <March 23, 2022> 135**

**Search Strategy:**

| **#** | **Searches** | **Results** |
| --- | --- | --- |
| #1 | MeSH descriptor: [Medicine, Chinese Traditional] explode all trees | 1237 |
| #2 | (Traditional Chinese Medicine OR Medicine, Chinese Traditional OR Traditional Medicine, Chinese OR Chinese Traditional Medicine OR Chinese Medicine, Traditional OR Drugs, Chinese Herbal OR Chinese Drugs, Plant OR Chinese Herbal Drugs OR Herbal Drugs, Chinese OR Plant Extracts, Chinese OR Chinese Plant Extracts OR Extracts, Chinese Plant OR Chinese medicine formula OR Chinese medicine decoction OR Medicine, Kampo OR “herbal formula” OR “herbal medicine” OR “herbal decoction” OR “herbal preparation” OR Chinese medicine injection OR Qingfei Paidu Decoction OR Huashi Baidu Formula OR Xuanfei Baidu Formula OR Chinese patent medicine OR Chinese medicine Granule OR Chinese medicine Capsule OR Jinhua Qinggan Granule OR Lianhua Qingwen Capsule OR Lianhua Qingwen Granule OR Xuebijing injection OR Xiyanping injection):ti,ab,kw | 14,264 |
| #3 | (coronavirus* OR coronovirus* OR coronavirinae* OR "2019‐nCoV" OR 2019nCoV OR “2019‐CoV” OR nCoV2019 OR "nCoV‐2019" OR "COVID‐19" OR COVID19 OR "CORVID‐19" OR CORVID19 OR "WN‐CoV" OR WNCoV OR "HCoV‐19" OR HCoV19 OR CoV OR "2019 novel*" OR Ncov OR "n‐cov" OR "SARS‐CoV‐2" OR "SARSCoV‐2" OR "SARSCoV2" OR "SARS‐CoV2" OR SARSCov19 OR "SARS‐Cov19" OR "SARSCov‐19" OR "SARS‐Cov‐19"):ti,ab,kw | 8,533 |
| #4 | #1 OR #2 | 14,419 |
| #5 | #3 AND #4 | 135 |

**Database: EMBASE <March 23, 2022> 323**

**Search Strategy:**

| **#** | **Searches** | **Results** |
| --- | --- | --- |
| #1 | 'systematic review (topic)'/exp | 27,535 |
| #2 | 'meta analysis (topic)'/exp | 47,430 |
| #3 | 'meta analysis'/exp | 229,181 |
| #4 | 'systematic review'/exp | 320,233 |
| #5 | 'multicenter study (topic)'/exp OR 'phase 2 clinical trial (topic)'/exp OR 'phase 3 clinical trial (topic)'/exp OR 'phase 4 clinical trial (topic)'/exp OR 'controlled clinical trial (topic)'/exp OR 'randomized controlled trial (topic)'/exp OR 'single blind procedure'/exp OR 'double blind procedure'/exp | 497,872 |
| #6 | coronavirus*:ab,ti OR coronovirus*:ab,ti OR coronavirinae*:ab,ti OR '2019‐ncov':ab,ti OR 2019ncov:ab,ti OR '2019‐cov':ab,ti OR ncov2019:ab,ti OR 'ncov‐2019':ab,ti OR 'covid‐19':ab,ti OR covid19:ab,ti OR 'corvid‐19':ab,ti OR corvid19:ab,ti OR 'wn‐cov':ab,ti OR wncov:ab,ti OR 'hcov‐19':ab,ti OR hcov19:ab,ti OR cov:ab,ti OR '2019 novel*':ab,ti OR ncov:ab,ti OR 'n‐cov':ab,ti OR 'sars‐cov‐2':ab,ti OR 'sarscov‐2':ab,ti OR 'sarscov2':ab,ti OR 'sars‐cov2':ab,ti OR sarscov19:ab,ti OR 'sars‐cov19':ab,ti OR 'sarscov‐19':ab,ti OR 'sars‐cov‐19':ab,ti | 207,164 |
| #7 | 'traditional chinese medicine':ab,ti OR 'medicine, chinese traditional':ab,ti OR 'traditional medicine, chinese':ab,ti OR 'chinese traditional medicine':ab,ti OR 'chinese medicine, traditional':ab,ti OR 'drugs, chinese herbal':ab,ti OR 'chinese drugs, plant':ab,ti OR 'chinese herbal drugs':ab,ti OR 'herbal drugs, chinese':ab,ti OR 'plant extracts, chinese':ab,ti OR 'chinese plant extracts':ab,ti OR 'extracts, chinese plant':ab,ti OR 'chinese medicine formula':ab,ti OR 'chinese medicine decoction':ab,ti OR 'medicine, kampo':ab,ti OR 'herbal formula':ab,ti OR 'herbal medicine':ab,ti OR 'herbal decoction':ab,ti OR 'herbal preparation':ab,ti OR 'chinese medicine injection':ab,ti OR 'qingfei paidu decoction':ab,ti OR 'huashi baidu formula':ab,ti OR 'xuanfei baidu formula':ab,ti OR 'chinese patent medicine':ab,ti OR 'chinese medicine granule':ab,ti OR 'chinese medicine capsule':ab,ti OR 'jinhua qinggan granule':ab,ti OR 'lianhua qingwen capsule':ab,ti OR 'lianhua qingwen granule':ab,ti OR 'xuebijing injection':ab,ti OR 'xiyanping injection':ab,ti | 51,224 |
| #8 | systematic*:ab,ti OR 'meta analysis':ab,ti OR 'meta analyses':ab,ti OR metaanalysis:ab,ti OR metanalysis:ab,ti OR metaanalyse:ab,ti OR metanalyses:ab,ti OR 'randomized controlled trial':ab,ti OR 'clinical trial':ab,ti OR 'controlled clinical trial':ab,ti OR random*:ab,ti OR blind*:ab,ti OR singleblind*:ab,ti OR doubleblind*:ab,ti OR trebleblind*:ab,ti OR tripleblind*:ab,ti | 2,662,347 |
| #9 | #1 OR #2 OR #3 OR #4 OR #5 OR #8 | 2,913,162 |
| #10 | 'chinese medicine'/exp | 59,668 |
| #11 | #7 OR #10 | 86,008 |
| #12 | #6 AND #9 AND #11 | 323 |

**Database: PubMed <March 23, 2022> 232**

**Search Strategy:**

| **#** | **Searches** | **Results** |
| --- | --- | --- |
| #1 | "Medicine, Chinese Traditional"[Mesh] | 21,351 |
| #2 | "Meta-Analysis" [Publication Type] OR "Meta-Analysis as Topic"[Mesh] | 166,911 |
| #3 | "Systematic Review" [Publication Type] OR "Systematic Reviews as Topic"[Mesh] | 182,313 |
| #4 | Traditional Chinese Medicine[Title/Abstract] OR Medicine, Chinese Traditional[Title/Abstract] OR Traditional Medicine, Chinese[Title/Abstract] OR Chinese Traditional Medicine[Title/Abstract] OR Chinese Medicine, Traditional[Title/Abstract] OR Drugs, Chinese Herbal[Title/Abstract] OR Chinese Drugs, Plant[Title/Abstract] OR Chinese Herbal Drugs[Title/Abstract] OR Herbal Drugs, Chinese[Title/Abstract] OR Plant Extracts, Chinese[Title/Abstract] OR Chinese Plant Extracts[Title/Abstract] OR Extracts, Chinese Plant[Title/Abstract] OR Chinese medicine formula[Title/Abstract] OR Chinese medicine decoction[Title/Abstract] OR Medicine, Kampo[Title/Abstract] OR "herbal formula"[Title/Abstract] OR "herbal medicine"[Title/Abstract] OR "herbal decoction"[Title/Abstract] OR "herbal preparation"[Title/Abstract] OR Chinese medicine injection[Title/Abstract] OR Qingfei Paidu Decoction[Title/Abstract] OR Huashi Baidu Formula[Title/Abstract] OR Xuanfei Baidu Formula[Title/Abstract] OR Chinese patent medicine[Title/Abstract] OR Chinese medicine Granule[Title/Abstract] OR Chinese medicine Capsule[Title/Abstract] OR Jinhua Qinggan Granule[Title/Abstract] OR Lianhua Qingwen Capsule[Title/Abstract] OR Lianhua Qingwen Granule[Title/Abstract] OR Xuebijing injection[Title/Abstract] OR Xiyanping injection[Title/Abstract] | 44,193 |
| #5 | coronavirus*[Title/Abstract] OR coronovirus*[Title/Abstract] OR coronavirinae*[Title/Abstract] OR "2019‐nCoV"[Title/Abstract] OR 2019nCoV[Title/Abstract] OR "2019‐CoV"[Title/Abstract] OR nCoV2019[Title/Abstract] OR "nCoV‐2019"[Title/Abstract] OR "COVID‐19"[Title/Abstract] OR COVID19[Title/Abstract] OR "CORVID‐19"[Title/Abstract] OR CORVID19[Title/Abstract] OR "WN‐CoV"[Title/Abstract] OR WNCoV[Title/Abstract] OR "HCoV‐19"[Title/Abstract] OR HCoV19[Title/Abstract] OR CoV[Title/Abstract] OR "2019 novel*"[Title/Abstract] OR Ncov[Title/Abstract] OR "n‐cov"[Title/Abstract] OR "SARS‐CoV‐2"[Title/Abstract] OR "SARSCoV‐2"[Title/Abstract] OR "SARSCoV2"[Title/Abstract] OR "SARS‐CoV2"[Title/Abstract] OR SARSCov19[Title/Abstract] OR "SARS‐Cov19"[Title/Abstract] OR "SARSCov‐19"[Title/Abstract] OR "SARS‐Cov‐19"[Title/Abstract] | 203,650 |
| #6 | systematic*[Title/Abstract] OR "meta-analysis"[Title/Abstract] OR metaanalysis[Title/Abstract] OR "meta analysis"[Title/Abstract] OR "meta analyses"[Title/Abstract] OR "meta-analysis"[Title/Abstract] OR "meta-analyses"[Title/Abstract] OR metaanalysis[Title/Abstract] OR metanalysis[Title/Abstract] OR metaanalyse[Title/Abstract] OR metanalyses[Title/Abstract] OR randomized controlled trial[Title/Abstract] OR Clinical trial[Title/Abstract] OR Controlled Clinical trial[Title/Abstract] OR random*[Title/Abstract] OR blind*[Title/Abstract] OR singleblind*[Title/Abstract] OR doubleblind*[Title/Abstract] OR trebleblind*[Title/Abstract] OR tripleblind*[Title/Abstract] | 2,001,382 |
| #7 | "Clinical Trials, Phase II as Topic"[Mesh] OR "Clinical Trials, Phase III as Topic"[Mesh] OR "Clinical Trials, Phase IV as Topic"[Mesh] OR "Controlled Clinical Trials as Topic"[Mesh] OR "Randomized Controlled Trials as Topic"[Mesh] OR "Intention to Treat Analysis"[Mesh] OR "Pragmatic Clinical Trials as Topic"[Mesh] OR "Clinical Trials, Phase II"[Publication Type] OR "Clinical Trials, Phase III"[Publication Type] OR "Clinical Trials, Phase IV"[Publication Type] OR "Controlled Clinical Trials"[Publication Type] OR "Randomized Controlled Trials"[Publication Type] OR "Pragmatic Clinical Trials as Topic"[Publication Type] OR "Single-Blind Method"[Mesh] OR "Double-Blind Method"[Mesh] | 358,927 |
| #8 | #1 OR #4 | 56,076 |
| #9 | #2 OR #3 OR #6 OR #7 | 2,110,529 |
| #10 | #5 AND #8 AND #9 | 232 |

**Database: Web of Science <March 23, 2022> 292**

**Search Strategy:**

| **#** | **Searches** | **Results** |
| --- | --- | --- |
| #1 | Traditional Chinese Medicine OR Medicine, Chinese Traditional OR Traditional Medicine, Chinese OR Chinese Traditional Medicine OR Chinese Medicine, Traditional OR Drugs, Chinese Herbal OR Chinese Drugs, Plant OR Chinese Herbal Drugs OR Herbal Drugs, Chinese OR Plant Extracts, Chinese OR Chinese Plant Extracts OR Extracts, Chinese Plant OR Chinese medicine formula OR Chinese medicine decoction OR Medicine, Kampo OR “herbal formula” OR “herbal medicine” OR “herbal decoction” OR “herbal preparation” OR Chinese medicine injection OR qinghai pandu Decoction OR haushi Baidu Formula OR xuanwei Baidu Formula OR Chinese patent medicine OR Chinese medicine Granule OR Chinese medicine Capsule OR Jinhua qinglan Granule OR lianhuan qingben Capsule OR lianhuan qingben Granule OR Xuebijing injection OR xifanping injection (Topic) **and** coronavirus* OR coronovirus* OR coronavirinae* OR "2019‐nCoV" OR 2019ncovr OR “2019‐CoV” OR ncovid19 OR "nCoV‐2019" OR "COVID‐19" OR COVID19 OR "CORVID‐19" OR covid19 OR "WN‐CoV" OR wecov OR "HCoV‐19" OR hcox19 OR CoV OR "2019 novel*" OR Ncov OR "n‐cov" OR "SARS‐CoV‐2" OR "SARSCoV‐2" OR "sarscov" OR "SARS‐CoV2" OR sarscov2 OR "SARS‐Cov19" OR "SARSCov‐19" OR "SARS‐Cov‐19" (Topic) **and** systematic* OR “meta-analysis” or metaanalysis OR “meta analysis” OR “meta analyses” OR “meta-analysis” OR “meta-analyses” OR metaanalysis OR metanalysis OR metaanalyses OR metanalysis OR randomized controlled trial OR Clinical trial OR Controlled Clinical trial OR random* OR blind* OR singleblind* OR doubleblind* OR trebleblind* OR tripleblind* (Topic) | 292 |

**Database: CBM <March 23, 2022> 294**

**Search Strategy:**

| **#** | **Searches** | **Results** |
| --- | --- | --- |
| #1 | "中草药"[不加权:扩展] OR "抗病毒药(中药)"[不加权:扩展] | 139,153 |
| #2 | "Meta分析"[不加权:扩展] | 28,424 |
| #3 | ("中国医学"[常用字段:智能] OR "中药"[常用字段:智能] OR "传统医学"[常用字段:智能] OR "中草药"[常用字段:智能] OR "中成药"[常用字段:智能] OR "中医药"[常用字段:智能] OR "中医学"[常用字段:智能] OR "中医"[常用字段:智能] OR "中药学"[常用字段:智能]) | 1,181,776 |
| #4 | ("新型冠状病毒肺炎"[常用字段:智能] OR "新冠肺炎"[常用字段:智能] OR "新型冠状病毒"[常用字段:智能]) | 24,181 |
| #5 | "随机对照试验"[不加权:扩展] OR "随机对照试验(主题)"[不加权:扩展] | 491,418 |
| #6 | ("系统评价"[常用字段:智能] OR "meta分析"[常用字段:智能] OR "荟萃分析"[常用字段:智能] OR "系统综述"[常用字段:智能]) | 48,980 |
| #7 | ("随机"[常用字段:智能] OR "干预"[常用字段:智能]) AND( "对照"[常用字段:智能] OR "分组"[常用字段:智能] OR "实验"[常用字段:智能] OR "试验"[常用字段:智能] OR "设计"[常用字段:智能] OR "研究"[常用字段:智能]) | 1,766,956 |
| #8 | (#4) OR (#2) | 1,189,377 |
| #9 | (#10) OR (#8) OR (#7) OR (#3) | 1,790,908 |
| #10 | (#12) AND (#11) AND (#6) | 294 |

**Database: CNKI <March 23, 2022> 204**

**Search Strategy:**

| **#** | **Searches** | **Results** |
| --- | --- | --- |
| #1 | (篇关摘：中国医学 + 中药 + 传统医学 + 中草药 + 中成药 + 中医药 + 中医学 + 中医 + 中药学 (精确)) AND (篇关摘：新型冠状病毒肺炎 + 新冠肺炎 + 新型冠状病毒 (精确)) AND (篇关摘：系统评价 + meta分析 + 荟萃分析 + 系统综述 + ((随机 + 干预) * (对照 + 分组 + 实验 + 试验 + 设计 + 研究)) (精确)) | 204 |

**Database: CQVIP <March 23, 2022> 185**

**Search Strategy:**

| **#** | **Searches** | **Results** |
| --- | --- | --- |
| #1 | 摘要=中国医学 OR 中药 OR 传统医学 OR 中草药 OR 中成药 OR 中医药 OR 中医学 OR 中医 OR 中药学 AND 摘要=新型冠状病毒肺炎 OR 新冠肺炎 OR 新型冠状病毒 AND 摘要=系统评价 OR meta分析 OR 荟萃分析 OR 系统综述 OR ((随机 OR 干预) AND (对照 OR 分组 OR 实验 OR 试验 OR 设计 OR 研究)) | 185 |

**Database: WanFang Data <March 23, 2022> 300**

**Search Strategy:**

| **#** | **Searches** | **Results** |
| --- | --- | --- |
| #1 | 主题:(中国医学 OR 中药 OR 传统医学 OR 中草药 OR 中成药 OR 中医药 OR 中医学 OR 中医 OR 中药学) and 主题:(新型冠状病毒肺炎 OR 新冠肺炎 OR 新型冠状病毒) and 主题:(系统评价 OR meta分析 OR 荟萃分析 OR 系统综述 OR ((随机 OR 干预) AND (对照 OR 分组 OR 实验 OR 试验 OR 设计 OR 研究))) | 300 |

**Database: Clinical trial registry** <March 23, 2022> 32

**Search terms:** Traditional Chinese Medicine OR Medicine, Chinese Traditional OR Traditional Medicine, Chinese OR Chinese Traditional Medicine OR Chinese Medicine, Traditional OR Drugs, Chinese Herbal OR Chinese Drugs, Plant OR Chinese Herbal Drugs OR Herbal Drugs, Chinese OR Plant Extracts, Chinese OR Chinese Plant Extracts OR Extracts, Chinese Plant OR Chinese medicine formula OR Chinese medicine decoction OR Medicine, Kampo OR “herbal formula” OR “herbal medicine” OR “herbal decoction” OR “herbal preparation” OR Chinese medicine injection OR qinghai pandu Decoction OR haushi Baidu Formula OR xuanwei Baidu Formula OR Chinese patent medicine OR Chinese medicine Granule OR Chinese medicine Capsule OR Jinhua qinglan Granule OR lianhuan qingben Capsule OR lianhuan qingben Granule OR Xuebijing injection OR xifanping injection

**Applied filters**: Condition or disease: COVID-19

**Database: WHO International Clinical Trials Registry Platform (ICTRP) Search Portal** <March 23, 2022> 187

**Search terms:** Traditional Chinese Medicine OR Medicine, Chinese Traditional OR Traditional Medicine, Chinese OR Chinese Traditional Medicine OR Chinese Medicine, Traditional OR Drugs, Chinese Herbal OR Chinese Drugs, Plant OR Chinese Herbal Drugs OR Herbal Drugs, Chinese OR Plant Extracts, Chinese OR Chinese Plant Extracts OR Extracts, Chinese Plant OR Chinese medicine formula OR Chinese medicine decoction OR Medicine, Kampo OR “herbal formula” OR “herbal medicine” OR “herbal decoction” OR “herbal preparation” OR Chinese medicine injection OR qinghai pandu Decoction OR haushi Baidu Formula OR xuanwei Baidu Formula OR Chinese patent medicine OR Chinese medicine Granule OR Chinese medicine Capsule OR Jinhua qinglan Granule OR lianhuan qingben Capsule OR lianhuan qingben Granule OR Xuebijing injection OR xifanping injection

**Applied filters:** Restrict to COVID-19

**Gray literature, reference lists of articles and relevant Chinese conference proceedings** <March 23, 2022> 26

**Table S2 Excluded studies: 36**

| **Title** | **Author, year** | **Reason for Exclude** | |
| --- | --- | --- | --- |
| Baidu Jieduan granules, traditional Chinese medicine, in the treatment of moderate coronavirus disease-2019 (COVID-19): study protocol for an open-label, randomized controlled clinical trial | Zhang, W, 2021 | Protocol | |
| Efficacy of the combination of modern medicine and traditional Chinese medicine in pulmonary fibrosis arising as a sequelae in convalescent COVID-19 patients: a randomized multicenter trial | Lu, ZH, 2021 | Protocol | |
| Moxibustion for abdominal pain in COVID-19: A protocol for systematic review and meta-analysis | Li, X, 2022 | Protocol | |
| Efficacy and safety of the combination of modern medicine and traditional Chinese medicine in pulmonary fibrosis caused by novel coronavirus disease: A protocol for Bayesian network meta-analysis | Li, F, 2021 | Protocol | |
| Limited evidence for Chinese drug therapy in COVID-19: systematic review and meta-analysis | Wiebrecht, A, 2020 | Comment | |
| The efficacy and safety of traditional Chinese medicines, modified Radix Fici Simplicissimae, combined with Western medicines amongst patients infected with the 2019 novel coronavirus (SARS-CoV-2) in tropical tourist area, China | Chen, J, 2021 | Letter | |
| Effects of non-drug interventions on depression, anxiety and sleep in COVID-19 patients: A systematic review and meta-analysis | Ding, H, 2021 | Wrong intervention | |
| Current treatment strategies for COVID‑19 (Review) | Han, F, 2021 | Wrong intervention | |
| Lactobacillus plantarum induces innate cytokine responses that potentially provide a protective benefit against COVID-19: A single-arm, double-blind, prospective trial combined with an in vitro cytokine response assay | Kageyama, Y, 2022 | Wrong intervention | |
| Efficacy of Persian medicine herbal formulations (capsules and decoction) compared to standard care in patients with COVID-19, a multicenter open-labeled, randomized, controlled clinical trial | Karimi, M, 2021 | Wrong intervention | |
| Sex-tailored pharmacology and COVID-19: Next steps towards appropriateness and health equity | Spini, A, 2021 | Wrong intervention | |
| 甘草酸二胺在普通型新型冠状病毒肺炎患者治疗中的临床价值 | 周外民, 2020 | Wrong intervention | |
| 新型冠状病毒肺炎治疗药物临床试验研究进展 | 胡晓, 2020 | Wrong intervention | |
| Application of Chinese medicine in the management of critical conditions: A review on sepsis | Fan, TT, 2020 | Wrong population | |
| Chinese herbal medicine for severe acute respiratory syndrome: A systematic review and meta-analysis | Liu, JP, 2004 | Wrong population | |
| 清热解毒类中药在病毒性呼吸系统疾病中的应用探讨 | 陈明会, 2020 | Wrong population | |
| 中西医结合治疗SARS的临床疗效观察 | 黄小波, 2004 | Wrong population | |
| Efficacy and safety assessment of severe COVID-19 patients with Chinese medicine: A retrospective case series study at early stage of the COVID-19 epidemic in Wuhan, China | Huang, L, 2021 | Wrong research design | |
| Effects of Tanreqing Capsule on the negative conversion time of nucleic acid in patients with COVID-19: A retrospective cohort study | Xing, ZA, 2020 | Wrong research design | |
| Association between Use of Qingfei Paidu Tang and Mortality in Hospitalized Patients with COVID-19: A national retrospective registry study | Zhang, L, 2020 | Wrong research design | |
| Association between early treatment with Qingfei Paidu decoction and favorable clinical outcomes in patients with COVID-19: A retrospective multicenter cohort study | Shi, N, 2020 | Wrong research design | |
| Synergic Role of Dexamethasone And Lianhua Qingwen Capsule Accelerate Nucleic Acid Negative Conversion In Severe COVID-19 Patients | Combret, Y, 2017 | Wrong research design | |
| Yindan Jiedu granules exhibit anti-inflammatory effect in patients with novel Coronavirus disease (COVID-19) by suppressing the NF-κB signaling pathway | Feng, Y, 2022 | Wrong research design | |
| Clinical evaluation of Shufeng Jiedu Capsules combined with umifenovir (Arbidol) in the treatment of common-type COVID-19: a retrospective study | Chen, J, 2020 | Wrong research design | |
| Positive effects of Lianhuaqingwen granules in COVID-19 patients: A retrospective study of 248 cases | Pan, S, 2021 | Wrong research design | |
| Traditional Chinese Medicine as a complementary therapy in combat with COVID-19-A review of evidence-based research and clinical practice | Xi, VW, 2020 | Wrong research design | |
| The Beneficial Role of Auricular Point Pressure in Insomnia and Anxiety in Isolated COVID-19 Patients | Luo, YM, 2021 | Wrong research design | |
| Arbidol combined with the Chinese medicine Lianhuaqingwen capsule versus arbidol alone in the treatment of COVID-19 | Liu, L, 2021 | Wrong research design | |
| The three syndromes and six Chinese patent medicine study during the recovery phase of COVID-19 | An, X, 2021 | Wrong research design | |
| 中西医结合治疗新型冠状病毒肺炎34例临床研究 | 夏文广, 2020 | Wrong research design | |
| 清肺排毒汤对痰热壅肺型新冠肺炎患者临床疗效的影响 | 曾宪红, 2020 | Wrong research design | |
| 采用清肺排毒汤联合西药 43 例与单用西药46 例的新型冠状病毒肺炎临床疗效比较 | 余雪源, 2020 | Wrong research design | |
| 血必净治疗新型冠状病毒肺炎的临床疗效观察 | 张从玉, 2020 | Wrong research design | |
| 疏风解毒胶囊联合阿比多尔治疗新型冠状病毒肺炎的回顾性研究 | 瞿香坤, 2020 | Wrong research design | |
| 51 例新型冠状病毒肺炎患者应用中药连花清瘟疗效分析：多中心回顾性研究 | 程德忠, 2020 | Wrong research design | |
| 阿比多尔、清肺排毒汤、连花清瘟胶囊、金叶败毒颗粒对某方舱医院轻型/普通型新冠肺炎患者疗效的回顾性研究 | 余恒毅, 2020 | Wrong research design | |
|  | | |  |

**Table S3. Summary of the included RCTs**

| **Study** | **Country** | **Sample size** | **Population** | **Intervention category** | **Abbreviations** | **TCM alone/Integrated Chinese and modern medicines** | **Health strategy** | **Setting** | **Control** | **Outcome** | **p-value** | **Adverse event** |
| --- | --- | --- | --- | --- | --- | --- | --- | --- | --- | --- | --- | --- |
| Xuedong An, 2021 | China | 123 | Confirmed or suspected COVID-19 | Jinhua Qinggan granules | JHQG | Integrated Chinese and modern medicines | Treatment | NR | Western medicine | Rate of shortness of breath recovery | P＞0.05 | NR |
|  |  |  |  |  |  |  |  |  |  | Rate of fever recovery | P＞0.05 |  |
|  |  |  |  |  |  |  |  |  |  | Rate of cough recovery | P＞0.05 |  |
|  |  |  |  |  |  |  |  |  |  | Rate of fatigue recovery | P＞0.05 |  |
|  |  |  |  |  |  |  |  |  |  | Rate of diarrhea disappearance | P＞0.05 |  |
|  |  |  |  |  |  |  |  |  |  | Rate of nausea/vomiting disappearance | P＞0.05 |  |
|  |  |  |  |  |  |  |  |  |  | Rate of anorexia disappearance | P＞0.05 |  |
|  |  |  |  |  |  |  |  |  |  | Rate of muscle pain disappearance | P＞0.05 |  |
|  |  |  |  |  |  |  |  |  |  | Rate of chest tightness disappearance | P＞0.05 |  |
|  |  |  |  |  |  |  |  |  |  | Treatment duration | P＞0.05 |  |
|  |  |  |  |  |  |  |  |  |  | Rate of disease aggravation | P＞0.05 |  |
|  |  |  |  |  |  |  |  |  |  | Viral nucleic acid negative conversion rate | P＞0.05 |  |
|  |  |  |  |  |  |  |  |  |  | Antiviral drugs use | P＞0.05 |  |
|  |  |  |  |  |  |  |  |  |  | Anti-infective drugs use | P＜0.05 |  |
| Xiangying Ai, 2020 | China | 98 | (COVID-19) Mix | Feiyan Yihao Chinese medicine granules | FYYH | Integrated Chinese and modern medicines | Treatment | Hospitalization | Western medicine | TCM symptom scores | P＜0.05 | No serious adverse events |
|  |  |  |  |  |  |  |  |  |  | Improvement rate of TCM syndrome score | P＜0.05 |  |
|  |  |  |  |  |  |  |  |  |  | LYM | P＜0.05 |  |
|  |  |  |  |  |  |  |  |  |  | D-dimer | P＞0.05 |  |
|  |  |  |  |  |  |  |  |  |  | ALB | P＞0.05 |  |
|  |  |  |  |  |  |  |  |  |  | LDH | P＜0.05 |  |
|  |  |  |  |  |  |  |  |  |  | LAC | P＞0.05 |  |
|  |  |  |  |  |  |  |  |  |  | Duration of hospital stay | P＜0.05 |  |
| Wenming Ban, 2021 | China | 120 | (COVID-19) NR | Four-step Method of TCM Language Therapy | Four-step Method | Integrated Chinese and modern medicines | Rehabilitation | Hospitalization | Western medicine | Depression score | P＜0.05 | NR |
|  |  |  |  |  |  |  |  |  |  | Anxiety score | P＜0.05 |  |
|  |  |  |  |  |  |  |  |  |  | Sleep quality scale | P＜0.05 |  |
|  |  |  |  |  |  |  |  |  |  | Time to viral assay conversion | P＜0.05 |  |
|  |  |  |  |  |  |  |  |  |  | Duration of hospital stay | P＜0.05 |  |
| Kequn Chai, 2021 | China | 150 | (COVID-19) NR | Feiyan 1+2 | FY 1+2 | Integrated Chinese and modern medicines | Treatment | Hospitalization | Western medicine | TCM symptom scores | P＜0.05 | No serious adverse events |
|  |  |  |  |  |  |  |  |  |  | Time to fever recovery | P＞0.05 |  |
|  |  |  |  |  |  |  |  |  |  | Time to viral assay conversion | P＞0.05 |  |
|  |  |  |  |  |  |  |  |  |  | Chest CT manifestations | P＜0.05 |  |
|  |  |  |  |  |  |  |  |  |  | MuLBSTA | P＜0.05 |  |
|  |  |  |  |  |  |  |  |  |  | Clinical cure rate | P＜0.05 |  |
| Zizhou Zheng, 2020 | China | 130 | (COVID-19) Mix | Xiaochaihu Decoction + Maxing Shigan Decoction | XCH + MXSG | Integrated Chinese and modern medicines | Treatment | Hospitalization | Western medicine | Clinical cure rate | P＞0.05 | NR |
|  |  |  |  |  |  |  |  |  |  | Total Effective Rate | P＜0.05 |  |
| chaowu chen,2021 | China | 60 | Non-severe COVID-19 | Lianhua Qingwen granules/capsules | LHQW | Integrated Chinese and modern medicines | Treatment | Hospitalization | Western medicine | IL-10 | P＜0.05 | No significant difference between the two groups (P＞0.05) |
|  |  |  |  |  |  |  |  |  |  | PCT | P＜0.05 |  |
|  |  |  |  |  |  |  |  |  |  | CRP | P＜0.05 |  |
|  |  |  |  |  |  |  |  |  |  | Time to fever recovery | P＞0.05 |  |
|  |  |  |  |  |  |  |  |  |  | Time to fatigue recovery | P＜0.05 |  |
|  |  |  |  |  |  |  |  |  |  | Time to cough recovery | P＜0.05 |  |
|  |  |  |  |  |  |  |  |  |  | Time to shortness of breath recovery | P＜0.05 |  |
|  |  |  |  |  |  |  |  |  |  | Rate of disease aggravation | P＜0.05 |  |
|  |  |  |  |  |  |  |  |  |  | Clinical recovery time | P＞0.05 |  |
|  |  |  |  |  |  |  |  |  |  | Time to viral assay conversion | P＞0.05 |  |
| Erhui chen, 2020 | China | 59 | Non-severe COVID-19 | Baduanjin exercise | BDJ | Integrated Chinese and modern medicines | Rehabilitation | Hospitalization | Western medicine | Rate of fatigue recovery | NR | NR |
|  |  |  |  |  |  |  |  |  |  | Anxiety score | P＜0.05 |  |
| liangzhong chen, 2020 | China | 30 | (COVID-19) NR | Xuebijing injection | XBJ | Integrated Chinese and modern medicines | Treatment | Hospitalization | Western medicine | Total Effective Rate | P＜0.05 | No significant difference between the two groups (P＞0.05) |
|  |  |  |  |  |  |  |  |  |  | CRP | P＜0.05 |  |
| suxin chen | China | 60 | (COVID-19) NR | Traditional Chinese Medicine | TCM | Integrated Chinese and modern medicines | Rehabilitation | Hospitalization | Western medicine | Anxiety score | P＜0.05 | NR |
|  |  |  |  |  |  |  |  |  |  | Depression score | P＜0.05 |  |
| Yuqin Chen,2021 | China | 131 | Convalescent COVID-19 | Bufei Huoxue capsules | BFHX | TCM alone | Rehabilitation | Hospitalization | Standard care | 6-Min Walk | P＜0.05 | No significant difference between the two groups (P＞0.05) |
|  |  |  |  |  |  |  |  |  |  | Chest CT Indexes Volume | P＜0.05 |  |
|  |  |  |  |  |  |  |  |  |  | Fatigue Assessment Inventory | P＜0.05 |  |
|  |  |  |  |  |  |  |  |  |  | Total St. George’s Respiratory Questionnaire (SGRQ) score | P＞0.05 |  |
|  |  |  |  |  |  |  |  |  |  | Borg Dyspnea score | P＞0.05 |  |
|  |  |  |  |  |  |  |  |  |  | TCM symptom scores | P＞0.05 |  |
|  |  |  |  |  |  |  |  |  |  | Chest CT manifestations | P＜0.05 |  |
| Shuang Zhou，2021 | China | 54 | (COVID-19) Mix | Shenhuang granules | SH | Integrated Chinese and modern medicines | Treatment | Hospitalization | Western medicine | Total Effective Rate | P＜0.05 | No significant difference between the two groups (P＞0.05) |
|  |  |  |  |  |  |  |  |  |  | Mortality rate | P＜0.05 |  |
|  |  |  |  |  |  |  |  |  |  | Rate of disease aggravation | P＜0.05 |  |
|  |  |  |  |  |  |  |  |  |  | Using an invasive ventilator | P＜0.05 |  |
|  |  |  |  |  |  |  |  |  |  | LYM | P＜0.05 |  |
|  |  |  |  |  |  |  |  |  |  | WBC | P＜0.05 |  |
|  |  |  |  |  |  |  |  |  |  | NEU | P＜0.05 |  |
| Fataneh Hashem-Dabaghian, 2021 | Iran | 43 | Confirmed or suspected COVID-19 | Lavender syrup decoction | Lavender syrup decoction | Integrated Chinese and modern medicines | Rehabilitation | Outpatient clinic | Western medicine |  |  | No serious adverse events |
|  |  |  |  |  |  |  |  |  |  | The VAS score for Olfactory dysfunction decreased | P＜0.05 |  |
| Qin He, 2021 | China | 72 | Non-severe COVID-19 | Buzhong Yiqi Decoction | BZYQ | Integrated Chinese and modern medicines | Treatment | NR | Western medicine | Improve clinical symptoms | P＜0.05 | NR |
|  |  |  |  |  |  |  |  |  |  | CRP | P＜0.05 |  |
|  |  |  |  |  |  |  |  |  |  | ESR | P＜0.05 |  |
|  |  |  |  |  |  |  |  |  |  | IL-6 | P＞0.05 |  |
|  |  |  |  |  |  |  |  |  |  | Clinical cure rate | P＜0.05 |  |
|  |  |  |  |  |  |  |  |  |  | TCM symptom scores | P＜0.05 |  |
| Qin He, 2020 | China | 64 | (COVID-19) NR | Shengmai Powder | SMP | Integrated Chinese and modern medicines | Treatment | NR | Western medicine | TCM symptom scores | P＜0.05 | NR |
|  |  |  |  |  |  |  |  |  |  | CRP | P＜0.05 |  |
|  |  |  |  |  |  |  |  |  |  | ESR | P＜0.05 |  |
|  |  |  |  |  |  |  |  |  |  | PCT | P＞0.05 |  |
|  |  |  |  |  |  |  |  |  |  | LYM | P＞0.05 |  |
|  |  |  |  |  |  |  |  |  |  | Pulse oxygen saturation | P＜0.05 |  |
|  |  |  |  |  |  |  |  |  |  | Chest CT manifestations | P＜0.05 |  |
|  |  |  |  |  |  |  |  |  |  | Viral nucleic acid negative conversion rate | P＜0.05 |  |
| Can Duan, 2020 | China | 123 | Non-severe COVID-19 | Jinhua Qinggan granules | JHQG | Integrated Chinese and modern medicines | Treatment | NR | Western medicine | Rate of fever recovery | P＜0.05 | The treatment group had a higher incidence（P＜0.05） |
|  |  |  |  |  |  |  |  |  |  | Rate of fatigue recovery | P＜0.05 |  |
|  |  |  |  |  |  |  |  |  |  | Rate of cough recovery | P＜0.05 |  |
|  |  |  |  |  |  |  |  |  |  | Rate of sputum disappearance | P＜0.05 |  |
|  |  |  |  |  |  |  |  |  |  | Rate of chills recovery | P＞0.05 |  |
|  |  |  |  |  |  |  |  |  |  | Rate of muscle pain disappearance | P＞0.05 |  |
|  |  |  |  |  |  |  |  |  |  | Rate of carebaria recovery | P＞0.05 |  |
|  |  |  |  |  |  |  |  |  |  | Rate of sore throat disappearance | P＞0.05 |  |
|  |  |  |  |  |  |  |  |  |  | Rate of sore throat disappearance | P＞0.05 |  |
|  |  |  |  |  |  |  |  |  |  | Rate of nasal congestion/runny nose recovery | P＞0.05 |  |
|  |  |  |  |  |  |  |  |  |  | Rate of nausea/vomiting disappearance | P＞0.05 |  |
|  |  |  |  |  |  |  |  |  |  | Rate of disease aggravation | P＞0.05 |  |
|  |  |  |  |  |  |  |  |  |  | TCM symptom scores | P＜0.05 |  |
|  |  |  |  |  |  |  |  |  |  | Anxiety score | P＜0.05 |  |
|  |  |  |  |  |  |  |  |  |  | Patient satisfaction | P＞0.05 |  |
| Ke Hu, 2021 | China | 284 | (COVID-19) NR | Lianhua Qingwen granules/capsules | LHQW | Integrated Chinese and modern medicines | Treatment | NR | Western medicine | Rate of fever recovery | P＜0.05 | No significant difference between the two groups (P＞0.05) |
|  |  |  |  |  |  |  |  |  |  | Time to fever recovery | P＜0.05 |  |
|  |  |  |  |  |  |  |  |  |  | Rate of cough recovery | P＜0.05 |  |
|  |  |  |  |  |  |  |  |  |  | Time to cough recovery | P＜0.05 |  |
|  |  |  |  |  |  |  |  |  |  | Rate of fatigue recovery | P＜0.05 |  |
|  |  |  |  |  |  |  |  |  |  | Time to fatigue recovery | P＜0.05 |  |
|  |  |  |  |  |  |  |  |  |  | Clinical cure rate | P＜0.05 |  |
|  |  |  |  |  |  |  |  |  |  | Chest CT manifestations | P＜0.05 |  |
|  |  |  |  |  |  |  |  |  |  | Clinical cure rate | P＜0.05 |  |
|  |  |  |  |  |  |  |  |  |  | Viral nucleic acid negative conversion rate | P＞0.05 |  |
|  |  |  |  |  |  |  |  |  |  | Rate of disease aggravation | P＞0.05 |  |
|  |  |  |  |  |  |  |  |  |  | Time to viral assay conversion | P＞0.05 |  |
| Lulu Li, 2021 | China | 118 | Convalescent COVID-19 | Shenling Baizhu Powder + Yiqi Huoxue Powder参苓白术散合益气活血散加减方+针灸 | SLBZ +YQHX | TCM alone | Rehabilitation | NR | Standard care | Quality Of Life (QOL) | P＞0.05 | NR |
|  |  |  |  |  |  |  |  |  |  | Anxiety score | P＞0.05 |  |
|  |  |  |  |  |  |  |  |  |  | Depression score | P＞0.05 |  |
|  |  |  |  |  |  |  |  |  |  | Rate of major symptoms recovery | NR |  |
|  |  |  |  |  |  |  |  |  |  | Recurrence of SARS-CoV-2 viral RNA positive | P＞0.05 |  |
|  |  |  |  |  |  |  |  |  |  | Abnormality rate of chest CT manifestations | P＞0.05 |  |
| Lan Li, 2021 | China | 60 | Non-severe COVID-19 | TCM Five Elements Music + Six Words Formula | FEM + SWF | TCM alone | Rehabilitation | NR | Standard care | Depression score | P＜0.05 | NR |
|  |  |  |  |  |  |  |  |  |  | Anxiety score | P＜0.05 |  |
|  |  |  |  |  |  |  |  |  |  | Sleep quality scale | P＜0.05 |  |
| Wenxue Ju, 2022 | China | 63 | Confirmed or suspected COVID-19 | TCM Psychotherapy | TCM-P | Integrated Chinese and modern medicines | Rehabilitation | Hospitalization | Western medicine | Depression score | P＜0.05 | NR |
|  |  |  |  |  |  |  |  |  |  | Anxiety score | P＜0.05 |  |
|  |  |  |  |  |  |  |  |  |  | Stress scores | P＜0.05 |  |
|  |  |  |  |  |  |  |  |  |  | The TCM Five Emotions | P＜0.05 |  |
|  |  |  |  |  |  |  |  |  |  | Total Effective Rate | P＜0.05 |  |
| Wei Jin, 2020 | China | 38 | Non-severe COVID-19 | Fufang Yinchai granules/Qingqiao Jiedu granule | FFYC + QQJD | Integrated Chinese and modern medicines | Treatment | Hospitalization | Western medicine | Rate of fever recovery | P＜0.05 | NR |
|  |  |  |  |  |  |  |  |  |  | Rate of cough recovery | P＞0.05 |  |
|  |  |  |  |  |  |  |  |  |  | Rate of fatigue recovery | P＞0.05 |  |
|  |  |  |  |  |  |  |  |  |  | Time to fever recovery | P＜0.05 |  |
|  |  |  |  |  |  |  |  |  |  | Time to cough recovery | P＜0.05 |  |
|  |  |  |  |  |  |  |  |  |  | Time to fatigue recovery | P＞0.05 |  |
|  |  |  |  |  |  |  |  |  |  | Rate of disease aggravation | P＞0.05 |  |
|  |  |  |  |  |  |  |  |  |  | Improvement rate | P＞0.05 |  |
|  |  |  |  |  |  |  |  |  |  | Discharge rate | P＞0.05 |  |
|  |  |  |  |  |  |  |  |  |  | Mortality rate | P＞0.05 |  |
|  |  |  |  |  |  |  |  |  |  | WBC | P＞0.05 |  |
|  |  |  |  |  |  |  |  |  |  | LYM | P＞0.05 |  |
|  |  |  |  |  |  |  |  |  |  | PCT | P＞0.05 |  |
|  |  |  |  |  |  |  |  |  |  | PaO2 | P＜0.05 |  |
|  |  |  |  |  |  |  |  |  |  | CRP | P＜0.05 |  |
|  |  |  |  |  |  |  |  |  |  | Chest CT manifestations | P＞0.05 |  |
| Li, L., 2021 | China | 200 | (COVID-19) NR | Shumian capsule | SM | TCM alone | Rehabilitation | Hospitalization | Standard care | Sleep quality scale | P＜0.05 | NR |
|  |  |  |  |  |  |  |  |  |  | Anxiety score | P＜0.05 |  |
|  |  |  |  |  |  |  |  |  |  | Depression score | P＞0.05 |  |
| Guorong Liao, 2020 | China | 35 | (COVID-19) NR | Chinese herbal decoction | CHD | Integrated Chinese and modern medicines | Treatment | Hospitalization | Western medicine | Rate of fever recovery | P＜0.05 | The control group had a higher incidence（P＜0.05） |
|  |  |  |  |  |  |  |  |  |  | Rate of fatigue recovery | P＜0.05 |  |
|  |  |  |  |  |  |  |  |  |  | Rate of cough recovery | P＜0.05 |  |
|  |  |  |  |  |  |  |  |  |  | Rate of diarrhea disappearance | P＜0.05 |  |
| Fan Liu, 2020 | China | 84 | (COVID-19) NR | Chinese herbal decoction | CHD | Integrated Chinese and modern medicines | Treatment | Hospitalization | Western medicine | Clinical cure rate | P＜0.05 | No serious adverse events |
|  |  |  |  |  |  |  |  |  |  | Total Effective Rate | P＜0.05 |  |
|  |  |  |  |  |  |  |  |  |  | Duration of hospital stay | P＜0.05 |  |
|  |  |  |  |  |  |  |  |  |  | Time to fever recovery | P＜0.05 |  |
|  |  |  |  |  |  |  |  |  |  | Treatment results (Mix) | P＜0.05 |  |
| Wu Liu, 2021 | China | 88 | Non-severe COVID-19 | Lianhua Qingwen granules/capsules + Feiyan 2 | LHQW + FY2 | Integrated Chinese and modern medicines | Treatment | Hospitalization | Western medicine | Total Effective Rate | P＜0.05 | The control group had a higher incidence（P＜0.05） |
| Jia Liu, 2021 | China | 204 | Non-severe COVID-19 | Huashibaidu Granule | HSBD | Integrated Chinese and modern medicines | Treatment | Hospitalization | Western medicine | Time to viral assay conversion | P＞0.05 | No serious adverse events |
|  |  |  |  |  |  |  |  |  |  | Time to fever recovery | P＜0.05 |  |
|  |  |  |  |  |  |  |  |  |  | blood routine test | P＞0.05 |  |
|  |  |  |  |  |  |  |  |  |  | blood biochemical test | P＞0.05 |  |
|  |  |  |  |  |  |  |  |  |  | Rate of cough recovery | P＜0.05 |  |
|  |  |  |  |  |  |  |  |  |  | Rate of fatigue recovery | P＜0.05 |  |
|  |  |  |  |  |  |  |  |  |  | Rate of chest tightness disappearance | P＜0.05 |  |
|  |  |  |  |  |  |  |  |  |  | Rate of headache recovery | P＞0.05 |  |
|  |  |  |  |  |  |  |  |  |  | Rate of sore throat disappearance | P＞0.05 |  |
|  |  |  |  |  |  |  |  |  |  | Chest CT manifestations | P＜0.05 |  |
| Yuanyuan Meng, 2020 | China | 100 | Non-severe COVID-19 | Comprehensive TCM Nursing | TCM Nursing | TCM alone | Treatment | Hospitalization | Standard care | Total Effective Rate | P＜0.05 | NR |
|  |  |  |  |  |  |  |  |  |  | Quality Of Life (QOL) | P＜0.05 |  |
| Min Qiu, 2020 | China | 50 | Non-severe COVID-19 | Maxing Xuanfei Jiedu Decoction | MXXFJD | Integrated Chinese and modern medicines | Treatment | Others | Western medicine | TCM symptom scores | P＜0.05 | NR |
|  |  |  |  |  |  |  |  |  |  | Time to fever recovery | P＜0.05 |  |
|  |  |  |  |  |  |  |  |  |  | Time to cough recovery | P＜0.05 |  |
|  |  |  |  |  |  |  |  |  |  | Chest CT manifestations | P＜0.05 |  |
|  |  |  |  |  |  |  |  |  |  | Rate of disease aggravation | P＜0.05 |  |
| Qiuyue Shi, 2020 | China | 60 | (COVID-19) NR | TCM emotional nursing | TCM-E | TCM alone | Rehabilitation | Hospitalization | Standard care | Anxiety score | P＜0.05 | NR |
|  |  |  |  |  |  |  |  |  |  | Depression score | P＜0.05 |  |
|  |  |  |  |  |  |  |  |  |  | Total Effective Rate | P＜0.05 |  |
|  |  |  |  |  |  |  |  |  |  | Patient satisfaction | P＜0.05 |  |
| Li Ni, 2021 | China | 235 | (COVID-19) NR | Shuanghuanglian oral liquids | SHL | Integrated Chinese and modern medicines | Treatment | Hospitalization | Western medicine | Time to disease recovery | P＞0.05 | No significant difference between the two groups (P＞0.05) |
|  |  |  |  |  |  |  |  |  |  | Viral nucleic acid negative conversion rate | P＜0.05 |  |
|  |  |  |  |  |  |  |  |  |  | Time to improvement of primary symptoms | P＞0.05 |  |
|  |  |  |  |  |  |  |  |  |  | Chest CT manifestations | P＜0.05 |  |
|  |  |  |  |  |  |  |  |  |  | Analyses of serum inflammatory factors and plasma markers of myocardial injury | P＞0.05 |  |
| Mesri, M., 2021 | Iran | 100 | Suspected COVID-19 | Zingiber officinale + Echinacea | Zingiber officinale + Echinacea | Integrated Chinese and modern medicines | Treatment | Outpatient clinic | Western medicine | Rate of cough recovery | P＜0.05 | NR |
|  |  |  |  |  |  |  |  |  |  | Rate of muscle pain disappearance | P＜0.05 |  |
|  |  |  |  |  |  |  |  |  |  | Rate of shortness of breath recovery | P＜0.05 |  |
|  |  |  |  |  |  |  |  |  |  | Rate of fever recovery | P＞0.05 |  |
|  |  |  |  |  |  |  |  |  |  | Rate of sore throat disappearance | P＞0.05 |  |
|  |  |  |  |  |  |  |  |  |  | Hospitalization rate | P＞0.05 |  |
| Qinhai Ma, 2021 | China | 50 | (COVID-19) NR | Reduning injection | RDN | Integrated Chinese and modern medicines | Treatment | Hospitalization | Western medicine | median time for the resolution of clinical symptoms | P＜0.05 | No significant difference between the two groups (P＞0.05) |
|  |  |  |  |  |  |  |  |  |  | Time to viral assay conversion | P＜0.05 |  |
|  |  |  |  |  |  |  |  |  |  | Duration of hospital stay | P＜0.05 |  |
|  |  |  |  |  |  |  |  |  |  | Time to fever recovery | P＜0.05 |  |
|  |  |  |  |  |  |  |  |  |  | Chest CT manifestations | P＜0.05 |  |
|  |  |  |  |  |  |  |  |  |  | symptom resolution rates | P＜0.05 |  |
|  |  |  |  |  |  |  |  |  |  | inflammatory cytokine production | P＜0.05 |  |
| Huimin Sun, 2020 | China | 57 | Non-severe COVID-19 | Lianhua Qingke Granule | LHQK | Integrated Chinese and modern medicines | Treatment | Hospitalization | Western medicine | Rate of cough recovery | P＜0.05 | NR |
|  |  |  |  |  |  |  |  |  |  | Time to cough recovery | P＜0.05 |  |
|  |  |  |  |  |  |  |  |  |  | Rate of sputum disappearance | P＜0.05 |  |
|  |  |  |  |  |  |  |  |  |  | Time to sputum recovery | P＜0.05 |  |
|  |  |  |  |  |  |  |  |  |  | Chest CT manifestations | P＜0.05 |  |
|  |  |  |  |  |  |  |  |  |  | OI | P＜0.05 |  |
|  |  |  |  |  |  |  |  |  |  | Rate of fever recovery | P＞0.05 |  |
|  |  |  |  |  |  |  |  |  |  | Rate of fatigue recovery | P＞0.05 |  |
|  |  |  |  |  |  |  |  |  |  | Rate of sore throat disappearance | P＞0.05 |  |
|  |  |  |  |  |  |  |  |  |  | Rate of disease aggravation | P＜0.05 |  |
| Dandan Song, 2021 | China | 60 | Non-severe COVID-19 | Suanzaoren Decoction | SZR | TCM alone | Rehabilitation | Hospitalization | Standard care | Anxiety score | P＜0.05 | NR |
| Suofang Shi 1, 2020 | China | 30 | Convalescent COVID-19 | Yiqi Yangyin Granule +Taiji Liuqi method | YQYY + TJLQ | TCM alone | Rehabilitation | Hospitalization | Standard care | TCM symptom scores | P＜0.05 | No serious adverse events |
|  |  |  |  |  |  |  |  |  |  | Total Effective Rate | P＜0.05 |  |
|  |  |  |  |  |  |  |  |  |  | PEF | P＜0.05 |  |
|  |  |  |  |  |  |  |  |  |  | CD3 | P＜0.05 |  |
|  |  |  |  |  |  |  |  |  |  | CD4 | P＜0.05 |  |
|  |  |  |  |  |  |  |  |  |  | CD8 | P＞0.05 |  |
|  |  |  |  |  |  |  |  |  |  | CD4/CD8 | P＞0.05 |  |
| Suofang Shi 2, 2020 | China | 120 | Convalescent COVID-19 | Futu Shengjin Rehabilitation Formul | FTSJ | TCM alone | Rehabilitation | Hospitalization | Standard care | Total Effective Rate | P＜0.05 | No serious adverse events |
|  |  |  |  |  |  |  |  |  |  | TCM symptom scores | P＜0.05 |  |
|  |  |  |  |  |  |  |  |  |  | IgA | P＜0.05 |  |
|  |  |  |  |  |  |  |  |  |  | IgM | P＜0.05 |  |
|  |  |  |  |  |  |  |  |  |  | IgG | P＜0.05 |  |
|  |  |  |  |  |  |  |  |  |  | C3 | P＜0.05 |  |
|  |  |  |  |  |  |  |  |  |  | C4 | P＜0.05 |  |
|  |  |  |  |  |  |  |  |  |  | CD3 | P＜0.05 |  |
|  |  |  |  |  |  |  |  |  |  | CD4 | P＜0.05 |  |
|  |  |  |  |  |  |  |  |  |  | CD8 | P＞0.05 |  |
|  |  |  |  |  |  |  |  |  |  | CD4/CD8 | P＞0.05 |  |
| Linqun Wang, 2020 | China | 118 | (COVID-19) Mix | Gegen Qinlian Pills | GGQL | Integrated Chinese and modern medicines | Treatment | Hospitalization | Western medicine | Rate of fever recovery | P＞0.05 | No serious adverse events |
|  |  |  |  |  |  |  |  |  |  | Rate of fatigue recovery | P＞0.05 |  |
|  |  |  |  |  |  |  |  |  |  | Rate of cough recovery | P＜0.05 |  |
|  |  |  |  |  |  |  |  |  |  | Rate of anorexia disappearance | P＞0.05 |  |
|  |  |  |  |  |  |  |  |  |  | Rate of chest tightness disappearance | P＜0.05 |  |
|  |  |  |  |  |  |  |  |  |  | Rate of nausea/vomiting disappearance | P＜0.05 |  |
|  |  |  |  |  |  |  |  |  |  | Rate of dyspnea recovery | P＞0.05 |  |
|  |  |  |  |  |  |  |  |  |  | Rate of headache recovery | P＞0.05 |  |
|  |  |  |  |  |  |  |  |  |  | Rate of sore throat disappearance | P＞0.05 |  |
|  |  |  |  |  |  |  |  |  |  | Rate of muscle pain disappearance | P＞0.05 |  |
|  |  |  |  |  |  |  |  |  |  | Rate of diarrhea disappearance | P＜0.05 |  |
|  |  |  |  |  |  |  |  |  |  | Rate of abdominal pain disappearance | P＞0.05 |  |
|  |  |  |  |  |  |  |  |  |  | Rate of aversion to cold recovery | P＞0.05 |  |
|  |  |  |  |  |  |  |  |  |  | Rate of nasal congestion/runny nose recovery | P＞0.05 |  |
|  |  |  |  |  |  |  |  |  |  | Gastrointestinal symptom score | P＜0.05 |  |
|  |  |  |  |  |  |  |  |  |  | Total symptom score | P＜0.05 |  |
|  |  |  |  |  |  |  |  |  |  | Rate of tongue picture recovery | P＜0.05 |  |
|  |  |  |  |  |  |  |  |  |  | WBC | P＞0.05 |  |
|  |  |  |  |  |  |  |  |  |  | NEU | P＞0.05 |  |
|  |  |  |  |  |  |  |  |  |  | LYM | P＜0.05 |  |
|  |  |  |  |  |  |  |  |  |  | CRP | P＜0.05 |  |
|  |  |  |  |  |  |  |  |  |  | PCT | P＜0.05 |  |
|  |  |  |  |  |  |  |  |  |  | Time to fever recovery | P＞0.05 |  |
|  |  |  |  |  |  |  |  |  |  | Time to viral assay conversion | P＜0.05 |  |
|  |  |  |  |  |  |  |  |  |  | Chest CT manifestations | P＞0.05 |  |
|  |  |  |  |  |  |  |  |  |  | Total Effective Rate | P＞0.05 |  |
| Lin Wang, 2020 | China | 80 | Non-severe COVID-19 | Shengmai Powder + Shenling Baizhu Powder | SMP + SLBZ | Integrated Chinese and modern medicines | Treatment | Hospitalization | Western medicine | Total Effective Rate | P＞0.05 | No serious adverse events |
|  |  |  |  |  |  |  |  |  |  | TCM symptom scores | P＜0.05 |  |
|  |  |  |  |  |  |  |  |  |  | Time to fever recovery | P＜0.05 |  |
|  |  |  |  |  |  |  |  |  |  | Time to cough recovery | P＜0.05 |  |
|  |  |  |  |  |  |  |  |  |  | Time to fatigue recovery | P＜0.05 |  |
|  |  |  |  |  |  |  |  |  |  | Time to anorexia recovery | P＜0.05 |  |
|  |  |  |  |  |  |  |  |  |  | Time to abdominal pain recovery | P＜0.05 |  |
|  |  |  |  |  |  |  |  |  |  | LYM | P＜0.05 |  |
|  |  |  |  |  |  |  |  |  |  | D-D | P＜0.05 |  |
|  |  |  |  |  |  |  |  |  |  | CD4+ | P＜0.05 |  |
|  |  |  |  |  |  |  |  |  |  | Rate of sore throat disappearance | P＞0.05 |  |
|  |  |  |  |  |  |  |  |  |  | ESR | P＞0.05 |  |
|  |  |  |  |  |  |  |  |  |  | PCT | P＞0.05 |  |
|  |  |  |  |  |  |  |  |  |  | CRP | P＞0.05 |  |
|  |  |  |  |  |  |  |  |  |  | CD8+ | P＞0.05 |  |
|  |  |  |  |  |  |  |  |  |  | Time to viral assay conversion | P＜0.05 |  |
|  |  |  |  |  |  |  |  |  |  | Chest CT manifestations | P＜0.05 |  |
| Jianming Wang, 2021 | China | 60 | Non-severe COVID-19 | Chinese herbal decoction | CHD | Integrated Chinese and modern medicines | Treatment | Hospitalization | Western medicine | Total Effective Rate | P＜0.05 | NR |
|  |  |  |  |  |  |  |  |  |  | TCM symptom scores | P＜0.05 |  |
|  |  |  |  |  |  |  |  |  |  | Time to fatigue recovery | P＜0.05 |  |
|  |  |  |  |  |  |  |  |  |  | Time to cough recovery | P＜0.05 |  |
|  |  |  |  |  |  |  |  |  |  | Time to fever recovery | P＜0.05 |  |
| Yali Wang, 2020 | China | 20 | (COVID-19) Mix | Chinese herbal decoction + Fumigation | CHD + Fumigation | Integrated Chinese and modern medicines | Treatment | Hospitalization | Western medicine | Time to fatigue recovery | P＞0.05 | NR |
|  |  |  |  |  |  |  |  |  |  | Time to cough recovery | P＞0.05 |  |
|  |  |  |  |  |  |  |  |  |  | Time to sore throat recovery | P＞0.05 |  |
|  |  |  |  |  |  |  |  |  |  | Time to shortness of breath recovery | P＞0.05 |  |
|  |  |  |  |  |  |  |  |  |  | Chest CT manifestations | P＞0.05 |  |
|  |  |  |  |  |  |  |  |  |  | Viral nucleic acid negative conversion rate | P＞0.05 |  |
| Fangdan Wang, 2020 | China | 16 | (COVID-19) NR | Baduanjin exercise | BDJ | TCM alone | Treatment | Hospitalization | Standard care | Total Effective Rate | P＜0.05 | No significant difference between the two groups (P＞0.05) |
|  |  |  |  |  |  |  |  |  |  | Time to fever recovery | P＜0.05 |  |
|  |  |  |  |  |  |  |  |  |  | Duration of hospital stay | P＜0.05 |  |
| WANG Jia-bo, 2020 | China | 48 | Suspected COVID-19 | Keguan-1 | Keguan-1 | Integrated Chinese and modern medicines | Treatment | Hospitalization | Western medicine | the incidents of ARDS development | P＜0.05 | No serious adverse events |
|  |  |  |  |  |  |  |  |  |  | Chest CT manifestations | P＜0.05 |  |
|  |  |  |  |  |  |  |  |  |  | Time to fever recovery | P＜0.05 |  |
| Jingling zhou, 2021 | China | 100 | (COVID-19) NR | Heat-sensitive Moxibustion | HSM | TCM alone | Rehabilitation | Others | Standard care | Skeletal muscle index | P＜0.05 | NR |
|  |  |  |  |  |  |  |  |  |  | grip strength | P＜0.05 |  |
|  |  |  |  |  |  |  |  |  |  | 6-Min Walk | P＜0.05 |  |
|  |  |  |  |  |  |  |  |  |  | Balance function | P＜0.05 |  |
|  |  |  |  |  |  |  |  |  |  | IgA | P＜0.05 |  |
|  |  |  |  |  |  |  |  |  |  | IgM | P＜0.05 |  |
|  |  |  |  |  |  |  |  |  |  | IgG | P＜0.05 |  |
|  |  |  |  |  |  |  |  |  |  | CD3 | P＜0.05 |  |
|  |  |  |  |  |  |  |  |  |  | CD4 | P＜0.05 |  |
|  |  |  |  |  |  |  |  |  |  | CD4/CD8 | P＜0.05 |  |
| Long Wen, 2020 | China | 60 | Severe COVID-19 | Xuebijing injection | XBJ | Integrated Chinese and modern medicines | Treatment | Hospitalization | Western medicine | WBC | P＜0.05 | No serious adverse events |
|  |  |  |  |  |  |  |  |  |  | LYM | P＜0.05 |  |
|  |  |  |  |  |  |  |  |  |  | APACHEⅡ score | P＜0.05 |  |
|  |  |  |  |  |  |  |  |  |  | CRP | P＜0.05 |  |
|  |  |  |  |  |  |  |  |  |  | ESR | P＜0.05 |  |
|  |  |  |  |  |  |  |  |  |  | Viral nucleic acid negative conversion rate | P＞0.05 |  |
| Yanshan Wei,2021 | China | 105 | Non-severe COVID-19 | Auricular Point Sticking + Acupoint Application | APS + AA | TCM alone | Treatment | Hospitalization | Standard care | Time to fever recovery | P＜0.05 | No serious adverse events |
|  |  |  |  |  |  |  |  |  |  | The frequency of defecation | P＜0.05 |  |
|  |  |  |  |  |  |  |  |  |  | Bris-tol | P＜0.05 |  |
|  |  |  |  |  |  |  |  |  |  | Sleep quality scale | P＜0.05 |  |
|  |  |  |  |  |  |  |  |  |  | Duration of hospital stay | P＜0.05 |  |
|  |  |  |  |  |  |  |  |  |  | Total Effective Rate | P＞0.05 |  |
| Yue Wang, 2021 | China | 140 | (COVID-19) NR | Qingfei Paidu Decoction | QFPD | Integrated Chinese and modern medicines | Treatment | Hospitalization | Western medicine | Total Effective Rate | P＜0.05 | No serious adverse events |
|  |  |  |  |  |  |  |  |  |  | TCM symptom scores | P＜0.05 |  |
|  |  |  |  |  |  |  |  |  |  | WBC | P＜0.05 |  |
|  |  |  |  |  |  |  |  |  |  | LYM | P＜0.05 |  |
|  |  |  |  |  |  |  |  |  |  | CRP | P＜0.05 |  |
|  |  |  |  |  |  |  |  |  |  | Duration of hospital stay | P＜0.05 |  |
| Mingzhong Xiao, 2020 | China | 283 | Confirmed or suspected COVID-19 | Huoxiang Zhengqi dropping pills+ Lianhua Qingwen granules/capsules | HXZQ+ LHQW | Integrated Chinese and modern medicines | Treatment | Others | Western medicine | Rate of fever recovery | P＞0.05 | NR |
|  |  |  |  |  |  |  |  |  |  | Rate of cough recovery | P＞0.05 |  |
|  |  |  |  |  |  |  |  |  |  | Rate of fatigue recovery | P＞0.05 |  |
|  |  |  |  |  |  |  |  |  |  | Rate of sore throat disappearance | P＞0.05 |  |
|  |  |  |  |  |  |  |  |  |  | Rate of diarrhea disappearance | P＞0.05 |  |
|  |  |  |  |  |  |  |  |  |  | Rate of anorexia disappearance | P＞0.05 |  |
|  |  |  |  |  |  |  |  |  |  | Rate of chest tightness disappearance | P＞0.05 |  |
|  |  |  |  |  |  |  |  |  |  | Rate of shortness of breath recovery | P＞0.05 |  |
|  |  |  |  |  |  |  |  |  |  | Rate of disease aggravation | P＞0.05 |  |
|  |  |  |  |  |  |  |  |  |  | The utilization rate of anti-infective drugs | P＜0.05 |  |
| Hongbo Zhou, 2021 | China | 40 | (COVID-19) NR | Hamao oil pieces | Hamao oil | Integrated Chinese and modern medicines | Treatment | Hospitalization | Western medicine | Rate of fever recovery | P＜0.05 | NR |
|  |  |  |  |  |  |  |  |  |  | Rate of fatigue recovery | P＜0.05 |  |
|  |  |  |  |  |  |  |  |  |  | Rate of dyspnea recovery | P＞0.05 |  |
|  |  |  |  |  |  |  |  |  |  | Rate of anorexia disappearance | P＜0.05 |  |
|  |  |  |  |  |  |  |  |  |  | Rate of cough recovery | P＜0.05 |  |
|  |  |  |  |  |  |  |  |  |  | Chest CT manifestations | P＜0.05 |  |
|  |  |  |  |  |  |  |  |  |  | Time to viral assay conversion | P＜0.05 |  |
| Xiangyong Yan, 2021 | China | 178 | Non-severe COVID-19 | Wenyang Huashi Prescription + Qingre Xuanfei Recipe | WYHS + QRXF | Integrated Chinese and modern medicines | Treatment | Hospitalization | Western medicine | Rate of fever recovery | P＜0.05 | NR |
|  |  |  |  |  |  |  |  |  |  | Rate of cough recovery | P＜0.05 |  |
|  |  |  |  |  |  |  |  |  |  | Rate of sputum disappearance | P＜0.05 |  |
|  |  |  |  |  |  |  |  |  |  | Rate of fatigue recovery | P＜0.05 |  |
|  |  |  |  |  |  |  |  |  |  | Rate of chest tightness disappearance | P＜0.05 |  |
|  |  |  |  |  |  |  |  |  |  | Rate of shortness of breath recovery | P＜0.05 |  |
|  |  |  |  |  |  |  |  |  |  | Rate of sore throat disappearance | P＜0.05 |  |
|  |  |  |  |  |  |  |  |  |  | Rate of anorexia disappearance | P＞0.05 |  |
|  |  |  |  |  |  |  |  |  |  | Rate of muscle pain disappearance | P＜0.05 |  |
|  |  |  |  |  |  |  |  |  |  | LYM | P＜0.05 |  |
|  |  |  |  |  |  |  |  |  |  | Rate of disease aggravation | P＜0.05 |  |
| Yan Xie, 2020 | China | 120 | Non COVID-19 | Heat-sensitive Moxibustion + Acupoint Application | HSM + AA | TCM alone | Prevention | Outpatient clinic | Standard care | CMI | P＜0.05 | NR |
|  |  |  |  |  |  |  |  |  |  | Sleep quality scale | P＜0.05 |  |
|  |  |  |  |  |  |  |  |  |  | IgA | P＜0.05 |  |
|  |  |  |  |  |  |  |  |  |  | IgG | P＜0.05 |  |
| Haiyan Wu, 2020 | China | 200 | (COVID-19) NR | TCM emotional nursing + Respiratory training | TCM-E + RT | Integrated Chinese and modern medicines | Rehabilitation | Hospitalization | Western medicine | Anxiety score | P＜0.05 | NR |
|  |  |  |  |  |  |  |  |  |  | Depression score | P＜0.05 |  |
| Xiaolong Xu, 2021 | China | 157 | (COVID-19) NR | Reduning injection | RDN | Integrated Chinese and modern medicines | Treatment | Hospitalization | Western medicine | Time to symptom resolution | P＜0.05 | No significant difference between the two groups (P＞0.05) |
|  |  |  |  |  |  |  |  |  |  | Time to viral assay conversion | P＜0.05 |  |
|  |  |  |  |  |  |  |  |  |  | Cure rates of symptoms 14days | P＜0.05 |  |
|  |  |  |  |  |  |  |  |  |  | Rate of symptom resolution | P＜0.05 |  |
|  |  |  |  |  |  |  |  |  |  | Duration of hospital stay | P＜0.05 |  |
|  |  |  |  |  |  |  |  |  |  | Time to fever recovery | P＜0.05 |  |
|  |  |  |  |  |  |  |  |  |  | The 28-day survival | P＞0.05 |  |
| Wuzhong Xiong, 2020 | China | 42 | (COVID-19) NR | Xuanfei Baidu Decoction | XFBD | Integrated Chinese and modern medicines | Treatment | Hospitalization | Western medicine | Rate of fever recovery | P＜0.05 | No serious adverse events |
|  |  |  |  |  |  |  |  |  |  | Rate of cough recovery | P＜0.05 |  |
|  |  |  |  |  |  |  |  |  |  | Rate of fatigue recovery | P＜0.05 |  |
|  |  |  |  |  |  |  |  |  |  | Rate of anorexia disappearance | P＜0.05 |  |
|  |  |  |  |  |  |  |  |  |  | Rate of shortness of breath recovery | P＞0.05 |  |
|  |  |  |  |  |  |  |  |  |  | Rate of chest tightness disappearance | P＞0.05 |  |
|  |  |  |  |  |  |  |  |  |  | Disappearance rate of Insomnia | P＞0.05 |  |
|  |  |  |  |  |  |  |  |  |  | Rate of sore throat disappearance | P＞0.05 |  |
|  |  |  |  |  |  |  |  |  |  | Disappearance rate of Chill | P＞0.05 |  |
|  |  |  |  |  |  |  |  |  |  | Rate of headache recovery | P＞0.05 |  |
|  |  |  |  |  |  |  |  |  |  | Rate of nausea/vomiting disappearance | P＞0.05 |  |
|  |  |  |  |  |  |  |  |  |  | Rate of diarrhea disappearance | P＞0.05 |  |
|  |  |  |  |  |  |  |  |  |  | WBC | P＜0.05 |  |
|  |  |  |  |  |  |  |  |  |  | LYM | P＜0.05 |  |
|  |  |  |  |  |  |  |  |  |  | CRP | P＜0.05 |  |
|  |  |  |  |  |  |  |  |  |  | ESR | P＜0.05 |  |
| Chao Yang, 2021 | China | 90 | (COVID-19) NR | Baduanjin exercise+Auricular Point Sticking | BDJ + APS | Integrated Chinese and modern medicines | Rehabilitation | Others | Western medicine | Sleep quality scale | P＜0.05 | NR |
|  |  |  |  |  |  |  |  |  |  | Anxiety score | P＞0.05 |  |
|  |  |  |  |  |  |  |  |  |  | Depression score | P＜0.05 |  |
|  |  |  |  |  |  |  |  |  |  | TCM symptom scores | NR |  |
|  |  |  |  |  |  |  |  |  |  | Total Effective Rate | P＞0.05 |  |
| Zancuo Yang, 2021 | China | 88 | Non-severe COVID-19 | Comprehensive TCM Nursing | TCM Nursing | Integrated Chinese and modern medicines | Rehabilitation | Hospitalization | Western medicine | Anxiety score | P＜0.05 | NR |
|  |  |  |  |  |  |  |  |  |  | Depression score | P＜0.05 |  |
|  |  |  |  |  |  |  |  |  |  | VAS score | P＜0.05 |  |
|  |  |  |  |  |  |  |  |  |  | Patient satisfaction | P＜0.05 |  |
| Yanqun Yang, 2021 | China | 34 | Suspected COVID-19 | Modified Jiegeng Xingren Decoction | JGXR | Integrated Chinese and modern medicines | Treatment | NR | Western medicine | Rate of fever recovery | P＞0.05 | No serious adverse events |
|  |  |  |  |  |  |  |  |  |  | Rate of cough recovery | P＞0.05 |  |
|  |  |  |  |  |  |  |  |  |  | Rate of fatigue recovery | P＞0.05 |  |
|  |  |  |  |  |  |  |  |  |  | Rate of anorexia disappearance | P＞0.05 |  |
|  |  |  |  |  |  |  |  |  |  | Rate of diarrhea disappearance | P＞0.05 |  |
|  |  |  |  |  |  |  |  |  |  | Rate of greasy fur recovery | P＜0.05 |  |
|  |  |  |  |  |  |  |  |  |  | Chest CT manifestations | P＜0.05 |  |
|  |  |  |  |  |  |  |  |  |  | WBC | P＞0.05 |  |
|  |  |  |  |  |  |  |  |  |  | ALT | P＞0.05 |  |
| Bohua Yan, 2020 | China | 22065 | Non COVID-19 | Jinhao Jiere granules + Huoxiangzhengqi oral liquids | JHJR + HXZQ | TCM alone | Prevention | Home/Community | Standard care | Incidence of cold-like symptoms | P＜0.05 | The treatment group had a higher incidence（P＜0.05） |
|  |  |  |  |  |  |  |  |  |  | COVID-19 infection | There were no cases of infection during the study |  |
|  |  |  |  |  |  |  |  |  |  | Protection rate of intervention | There were no cases of infection during the study |  |
| Dan Zhang, 2021 | China | 480 | Non COVID-19 | Fuzheng Gubiao Fanggan particles | FZGBFG | TCM alone | Prevention | NR | Standard care | Incidence of COVID-19 cases | P＜0.05 | No serious adverse events |
|  |  |  |  |  |  |  |  |  |  | The incidence of fever symptoms | P＜0.05 |  |
|  |  |  |  |  |  |  |  |  |  | The incidence of cough symptoms | P＜0.05 |  |
|  |  |  |  |  |  |  |  |  |  | The incidence of fatigue symptoms | P＜0.05 |  |
|  |  |  |  |  |  |  |  |  |  | The incidence of sore throat symptoms | P＜0.05 |  |
|  |  |  |  |  |  |  |  |  |  | The incidence of nasal congestion recovery symptoms | P＜0.05 |  |
|  |  |  |  |  |  |  |  |  |  | The incidence of runny nose symptoms | P＜0.05 |  |
|  |  |  |  |  |  |  |  |  |  | The incidence of diarrhea symptoms | P＜0.05 |  |
| Ping Yu, 2020 | China | 295 | Non-severe COVID-19 | Lianhua Qingwen granules/capsules | LHQW | Integrated Chinese and modern medicines | Treatment | Hospitalization | Western medicine | Total Effective Rate | P＜0.05 | No serious adverse events |
|  |  |  |  |  |  |  |  |  |  | Rate of disease aggravation | P＜0.05 |  |
|  |  |  |  |  |  |  |  |  |  | TCM symptom scores | P＜0.05 |  |
|  |  |  |  |  |  |  |  |  |  | WBC | P＜0.05 |  |
|  |  |  |  |  |  |  |  |  |  | LYM | P＜0.05 |  |
|  |  |  |  |  |  |  |  |  |  | CRP | P＜0.05 |  |
|  |  |  |  |  |  |  |  |  |  | PCT | P＜0.05 |  |
|  |  |  |  |  |  |  |  |  |  | Chest CT manifestations | P＞0.05 |  |
| Congcong Zeng, 2021 | China | 59 | Non-severe COVID-19 | Maxingshigan-Weijing Decoction | MXSGWJ | Integrated Chinese and modern medicines | Treatment | Hospitalization | Western medicine | Time to fever recovery | P＜0.05 | No serious adverse events |
|  |  |  |  |  |  |  |  |  |  | Time to fatigue recovery | P＜0.05 |  |
|  |  |  |  |  |  |  |  |  |  | Time to cough recovery | P＜0.05 |  |
|  |  |  |  |  |  |  |  |  |  | Time to dyspnea recovery | P＜0.05 |  |
|  |  |  |  |  |  |  |  |  |  | TCM symptom scores | P＜0.05 |  |
|  |  |  |  |  |  |  |  |  |  | Time to viral assay conversion | P＞0.05 |  |
|  |  |  |  |  |  |  |  |  |  | WBC | P＞0.05 |  |
|  |  |  |  |  |  |  |  |  |  | NEU | P＞0.05 |  |
|  |  |  |  |  |  |  |  |  |  | LYM | P＞0.05 |  |
|  |  |  |  |  |  |  |  |  |  | Red blood cells | P＞0.05 |  |
|  |  |  |  |  |  |  |  |  |  | Platelets | P＞0.05 |  |
|  |  |  |  |  |  |  |  |  |  | Hemoglobin | P＜0.05 |  |
| Xudong Yang， 2022 | China | 40 | (COVID-19) Mix | Traditional Chinese Medicine | TCM | Integrated Chinese and modern medicines | Treatment | Hospitalization | Western medicine | shortened the time of disease recovery | P＜0.05 | No serious adverse events |
|  |  |  |  |  |  |  |  |  |  | shortened the time of tongue amelioration | P＜0.05 |  |
|  |  |  |  |  |  |  |  |  |  | shortened the time of symptom disappearance | P＜0.05 |  |
|  |  |  |  |  |  |  |  |  |  | Virus removal time | P＜0.05 |  |
|  |  |  |  |  |  |  |  |  |  | Chest CT manifestations | P＜0.05 |  |
|  |  |  |  |  |  |  |  |  |  | CRP | P＜0.05 |  |
|  |  |  |  |  |  |  |  |  |  | ESR | P＜0.05 |  |
|  |  |  |  |  |  |  |  |  |  | PCT | P＜0.05 |  |
|  |  |  |  |  |  |  |  |  |  | Inflammatory factor | P＜0.05 |  |
|  |  |  |  |  |  |  |  |  |  | WBC | P＜0.05 |  |
|  |  |  |  |  |  |  |  |  |  | NEU | P＜0.05 |  |
|  |  |  |  |  |  |  |  |  |  | LYM | P＜0.05 |  |
|  |  |  |  |  |  |  |  |  |  | Rate of breathing | P＜0.05 |  |
|  |  |  |  |  |  |  |  |  |  | PCO2 | P＜0.05 |  |
|  |  |  |  |  |  |  |  |  |  | PO2 | P＜0.05 |  |
| Yanyun Zheng, 2010 | China | 60 | Non-severe COVID-19 | TCM features humanistic care | TCM-HC | TCM alone | Rehabilitation | Hospitalization | Standard care | TCM symptom scores | P＜0.05 | NR |
|  |  |  |  |  |  |  |  |  |  | Anxiety score | P＜0.05 |  |
|  |  |  |  |  |  |  |  |  |  | Depression score | P＜0.05 |  |
|  |  |  |  |  |  |  |  |  |  | Behavior on Medicine | P＜0.05 |  |
|  |  |  |  |  |  |  |  |  |  | Duration of hospital stay | P＜0.05 |  |
|  |  |  |  |  |  |  |  |  |  | Rate of disease aggravation | P＜0.05 |  |
| Jialiang Zhao, 2021 | China | 96 | Non-severe COVID-19 | Kangbingdu 1 | KBD 1 | Integrated Chinese and modern medicines | Treatment | Hospitalization | Western medicine | Total Effective Rate | P＜0.05 | NR |
|  |  |  |  |  |  |  |  |  |  | TCM symptom scores | P＜0.05 |  |
|  |  |  |  |  |  |  |  |  |  | LYM | P＜0.05 |  |
|  |  |  |  |  |  |  |  |  |  | CRP | P＜0.05 |  |
|  |  |  |  |  |  |  |  |  |  | PCT | P＜0.05 |  |
|  |  |  |  |  |  |  |  |  |  | LDH | P＜0.05 |  |
|  |  |  |  |  |  |  |  |  |  | Chest CT manifestations | P＜0.05 |  |
| Feng Zhao, 2020 | China | 52 | Convalescent COVID-19 | Xuanfei Dayu Decoction | XFDY | TCM alone | Rehabilitation | Home/Community | Standard care | WBC | P＞0.05 | No significant difference between the two groups (P＞0.05) |
|  |  |  |  |  |  |  |  |  |  | LYM | P＞0.05 |  |
|  |  |  |  |  |  |  |  |  |  | NEU | P＞0.05 |  |
|  |  |  |  |  |  |  |  |  |  | ESR | P＜0.05 |  |
|  |  |  |  |  |  |  |  |  |  | Liver function | P＞0.05 |  |
|  |  |  |  |  |  |  |  |  |  | TCM symptom scores | P＜0.05 |  |
|  |  |  |  |  |  |  |  |  |  | Chest CT manifestations | P＜0.05 |  |
| Chen Zhao, 2021 | China | 408 | Non-severe COVID-19 | Huashibaidu Granule | HSBD | Integrated Chinese and modern medicines | Treatment | Hospitalization | Western medicine | Rate of disease aggravation | P＜0.05 | No significant difference between the two groups (P＞0.05) |
|  |  |  |  |  |  |  |  |  |  | Duration of hospital stay | P＞0.05 |  |
|  |  |  |  |  |  |  |  |  |  | Discharge rate | P＞0.05 |  |
|  |  |  |  |  |  |  |  |  |  | Rate of fever recovery | P＞0.05 |  |
|  |  |  |  |  |  |  |  |  |  | Rate of cough recovery | P＞0.05 |  |
|  |  |  |  |  |  |  |  |  |  | Rate of fatigue recovery | P＞0.05 |  |
|  |  |  |  |  |  |  |  |  |  | Patients taking Arbidol hydrochloride during the trial, n (%) | P＞0.05 |  |
|  |  |  |  |  |  |  |  |  |  | Patients taking Chinese Medicine (CM) after 7-day treatment, n (%) | P＞0.05 |  |
| Xinyi Zhang, 2021 | China | 130 | Non-severe COVID-19 | Xiyanping injection | XYP | Integrated Chinese and modern medicines | Treatment | Hospitalization | Western medicine | Time to complete symptom resolution (days) decrease | P＜0.05 | No serious adverse events |
|  |  |  |  |  |  |  |  |  |  | Time to fever recovery | P＞0.05 |  |
|  |  |  |  |  |  |  |  |  |  | Time to cough recovery | P＜0.05 |  |
|  |  |  |  |  |  |  |  |  |  | Time to viral assay conversion | P＜0.05 |  |
|  |  |  |  |  |  |  |  |  |  | Rate of disease aggravation | P＜0.05 |  |
| Ling Zhang, 2022 | China | 144 | Non-severe COVID-19 | Lianhua Qingke tablets | LHQK | Integrated Chinese and modern medicines | Treatment | Hospitalization | routine treatment(supportive oxygen therapy, administration of antivirals, and symptom management ) | overall symptom resolution rate | P＜0.05 | No significant difference between the two groups (P＞0.05) |
|  |  |  |  |  |  |  |  |  |  | median time to symptom resolution | P＜0.05 |  |
|  |  |  |  |  |  |  |  |  |  | Rate of cough recovery | P＜0.05 |  |
|  |  |  |  |  |  |  |  |  |  | Rate of sputum disappearance | P＜0.05 |  |
|  |  |  |  |  |  |  |  |  |  | Time to cough recovery | P＜0.05 |  |
|  |  |  |  |  |  |  |  |  |  | Time to sputum recovery | P＜0.05 |  |
|  |  |  |  |  |  |  |  |  |  | Chest CT manifestations | P＜0.05 |  |
| Yusheng Yan, 2021 | China | 40 | Non-severe COVID-19 | Yinghuang Qingfei Capsule | YHQF | Integrated Chinese and modern medicines | Treatment | Hospitalization | routine treatment(supportive oxygen therapy, administration of antivirals, and symptom management ) | Total Effective Rate | P＞0.05 | No significant difference between the two groups (P＞0.05) |
|  |  |  |  |  |  |  |  |  |  | Clinical cure rate | P＞0.05 |  |
|  |  |  |  |  |  |  |  |  |  | Time to fever recovery | P＜0.05 |  |
|  |  |  |  |  |  |  |  |  |  | Time to cough recovery | P＜0.05 |  |
|  |  |  |  |  |  |  |  |  |  | CRP | P＜0.05 |  |
|  |  |  |  |  |  |  |  |  |  | PCT | P＜0.05 |  |
| Fen Hu, 2020 | China | 300 | Non-severe COVID-19 | Jinyinhua Oral Liquid | JYH | Integrated Chinese and modern medicines | Treatment | Hospitalization | routine treatment(supportive oxygen therapy, administration of antivirals, and symptom management ) | Viral nucleic acid negative conversion rate | P＞0.05 | No significant difference between the two groups (P＞0.05) |
|  |  |  |  |  |  |  |  |  |  | Chest CT manifestations | P＜0.05 |  |
|  |  |  |  |  |  |  |  |  |  | Duration of hospital stay | P＜0.05 |  |
|  |  |  |  |  |  |  |  |  |  | Rate of disease aggravation | P＜0.05 |  |
|  |  |  |  |  |  |  |  |  |  | Biochemical indexes | P＞0.05 |  |
| Kaifeng Liu, 2020 | China | 20 | Non-severe COVID-19 | Xuebijing injection | XBJ | Integrated Chinese and modern medicines | Treatment | Hospitalization | routine treatment(supportive oxygen therapy, administration of antivirals, and symptom management ) | Body temperature | P＞0.05 | No serious adverse events |
|  |  |  |  |  |  |  |  |  |  | WBC | P＞0.05 |  |
|  |  |  |  |  |  |  |  |  |  | LYM | P＜0.05 |  |
|  |  |  |  |  |  |  |  |  |  | CRP | P＜0.05 |  |
|  |  |  |  |  |  |  |  |  |  | ESR | P＜0.05 |  |
| Xiaolong Chen, 2020 | China | 40 | (COVID-19) NR | Zhenqi Fuzheng capsule | ZQFZ | Integrated Chinese and modern medicines | Rehabilitation | Hospitalization | Western medicine | Immune Function | P＜0.05 | NR |
| Yadong Li, 2020 | China | 12 | Severe COVID-19 | Qingfei Paidu Decoction | QFPD | Integrated Chinese and modern medicines | Treatment | Hospitalization | routine treatment(supportive oxygen therapy, administration of antivirals, and symptom management ) | WBC | P＜0.05 | The treatment group had a higher incidence（P＜0.05） |
|  |  |  |  |  |  |  |  |  |  | PCO2 | P＜0.05 |  |
|  |  |  |  |  |  |  |  |  |  | PO2 | P＜0.05 |  |
|  |  |  |  |  |  |  |  |  |  | Duration of hospital stay | P＜0.05 |  |
|  |  |  |  |  |  |  |  |  |  | Chest CT manifestations | P＜0.05 |  |
|  |  |  |  |  |  |  |  |  |  | Viral nucleic acid negative conversion rate | P＜0.05 |  |
|  |  |  |  |  |  |  |  |  |  | Total Effective Rate | P＜0.05 |  |
| Yongjiang Liu, 2021 | China | 50 | Severe COVID-19 | Huashibaidu Formula | HSBD | Integrated Chinese and modern medicines | Treatment | Hospitalization | routine treatment(supportive oxygen therapy, administration of antivirals, and symptom management ) | Total Effective Rate | P＜0.05 | The control group had a higher incidence（P＜0.05） |
|  |  |  |  |  |  |  |  |  |  | WBC | P＜0.05 |  |
|  |  |  |  |  |  |  |  |  |  | LYM | P＜0.05 |  |
|  |  |  |  |  |  |  |  |  |  | CRP | P＜0.05 |  |
|  |  |  |  |  |  |  |  |  |  | ESR | P＜0.05 |  |
| Youli Zhang, 2020 | ChinaChina | 120 | Non-severe COVID-19 | Jinyinhua Oral Liquid | JYH | Integrated Chinese and modern medicines | Treatment | Hospitalization | routine treatment(supportive oxygen therapy, administration of antivirals, and symptom management ) | Rate of fever recovery | P＞0.05 | No significant difference between the two groups (P＞0.05) |
|  |  |  |  |  |  |  |  |  |  | Rate of fatigue recovery | P＞0.05 |  |
|  |  |  |  |  |  |  |  |  |  | Rate of cough recovery | P＜0.05 |  |
|  |  |  |  |  |  |  |  |  |  | Chest CT manifestations | P＜0.05 |  |
|  |  |  |  |  |  |  |  |  |  | Viral nucleic acid negative conversion rate | P＞0.05 |  |
| Xiaolong Chen, 2020 | ChinaChina | 34 | (COVID-19) NR | Feilike capsule | FLK | Integrated Chinese and modern medicines | Treatment | Hospitalization | routine treatment(supportive oxygen therapy, administration of antivirals, and symptom management ) | Total Effective Rate | P＜0.05 | No serious adverse events |
| Shiqi Sun, 2021 | ChinaChina | 80 | (COVID-19) Mix | Liushen Pill | LS | Integrated Chinese and modern medicines | Treatment | Hospitalization | routine treatment(supportive oxygen therapy, administration of antivirals, and symptom management ) | Time to fever recovery | P＜0.05 | No serious adverse events |
|  |  |  |  |  |  |  |  |  |  | Total Effective Rate | P＜0.05 |  |
|  |  |  |  |  |  |  |  |  |  | TCM symptom scores | P＜0.05 |  |
|  |  |  |  |  |  |  |  |  |  | Rate of sore throat disappearance | P＜0.05 |  |
|  |  |  |  |  |  |  |  |  |  | Rate of dry stool recovery | P＜0.05 |  |
|  |  |  |  |  |  |  |  |  |  | Rate of cough recovery | P＞0.05 |  |
|  |  |  |  |  |  |  |  |  |  | Rate of fatigue recovery | P＞0.05 |  |
|  |  |  |  |  |  |  |  |  |  | Rate of nasal congestion/runny nose recovery | P＞0.05 |  |
|  |  |  |  |  |  |  |  |  |  | Rate of muscle pain disappearance | P＞0.05 |  |
|  |  |  |  |  |  |  |  |  |  | Rate of anorexia disappearance | P＞0.05 |  |
|  |  |  |  |  |  |  |  |  |  | Rate of dry mouth recovery | P＞0.05 |  |
|  |  |  |  |  |  |  |  |  |  | Rate of diarrhea disappearance | P＞0.05 |  |
| Zhijian Luo, 2021 | China | 60 | Severe COVID-19 | Xuebijing injection | XBJ | Integrated Chinese and modern medicines | Treatment | Hospitalization | routine treatment(supportive oxygen therapy, administration of antivirals, and symptom management ) | ARDS | P＜0.05 | No serious adverse events |
|  |  |  |  |  |  |  |  |  |  | Mechanical ventilation | P＜0.05 |  |
|  |  |  |  |  |  |  |  |  |  | IMV | P＜0.05 |  |
|  |  |  |  |  |  |  |  |  |  | NMV | P＜0.05 |  |
|  |  |  |  |  |  |  |  |  |  | Septic shock | P＜0.05 |  |
|  |  |  |  |  |  |  |  |  |  | 28-day mortality | P＞0.05 |  |
|  |  |  |  |  |  |  |  |  |  | Rate of disease aggravation | P＜0.05 |  |
|  |  |  |  |  |  |  |  |  |  | severe develop into moderate cases | P＜0.05 |  |
|  |  |  |  |  |  |  |  |  |  | Time to fever recovery | P＜0.05 |  |
|  |  |  |  |  |  |  |  |  |  | Time to cough recovery | P＜0.05 |  |
|  |  |  |  |  |  |  |  |  |  | Time to shortness of breath recovery | P＜0.05 |  |
|  |  |  |  |  |  |  |  |  |  | Time to fatigue recovery | P＜0.05 |  |
|  |  |  |  |  |  |  |  |  |  | The length of ICU stay | P＜0.05 |  |
|  |  |  |  |  |  |  |  |  |  | Leucocytes | P＞0.05 |  |
|  |  |  |  |  |  |  |  |  |  | LYM | P＜0.05 |  |
|  |  |  |  |  |  |  |  |  |  | CRP | P＜0.05 |  |
|  |  |  |  |  |  |  |  |  |  | IL-6 | P＜0.05 |  |
|  |  |  |  |  |  |  |  |  |  | IL-8 | P＜0.05 |  |
|  |  |  |  |  |  |  |  |  |  | TNF | P＜0.05 |  |

**Table S4. Summary of the included SRs**

| **Study** | **Country** | **Number of RCTs** | **Population** | **Intervention category** | **Abbreviations** | **TCM alone/Integrated Chinese and modern medicines** | **Health strategy** | **Setting** | **Control** | **Outcome** | **p-value** | **Adverse event** |
| --- | --- | --- | --- | --- | --- | --- | --- | --- | --- | --- | --- | --- |
| Lin Ang, 2020 | Korea | 7 | COVID-19 (NR) | Traditional Chinese medicine | TCM | Integrated Chinese and modern medicines | Treatment | NR | Western medicine | Total Effective Rate | P＜0.05 | No significant difference between the two groups (P＞0.05) |
|  |  |  |  |  |  |  |  |  |  | Rate of cough recovery | P＜0.05 |  |
|  |  |  |  |  |  |  |  |  |  | Rate of sputum disappearance | P＜0.05 |  |
|  |  |  |  |  |  |  |  |  |  | TCM syndrome score | P＜0.05 |  |
|  |  |  |  |  |  |  |  |  |  | WBC | P＜0.05 |  |
|  |  |  |  |  |  |  |  |  |  | LYM | P＜0.05 |  |
|  |  |  |  |  |  |  |  |  |  | CRP | P＜0.05 |  |
|  |  |  |  |  |  |  |  |  |  | Total procalcitonin level | P＜0.05 |  |
|  |  |  |  |  |  |  |  |  |  | Duration of Symptoms | P＜0.05 |  |
|  |  |  |  |  |  |  |  |  |  | Anxiety | P＜0.05 |  |
|  |  |  |  |  |  |  |  |  |  | Chest CT manifestations | NR |  |
|  |  |  |  |  |  |  |  |  |  | Oxygenation Index | P＜0.05 |  |
|  |  |  |  |  |  |  |  |  |  | Hospital Discharge Rate | P＜0.05 |  |
|  |  |  |  |  |  |  |  |  |  | Composite Events | NR |  |
| Xin Cai, 2020 | China | 6 | COVID-19 (NR) | Traditional Chinese medicine | TCM | Integrated Chinese and modern medicines | Treatment | NR | Western medicine | Total Effective Rate | P＜0.05 | No significant difference between the two groups (P＞0.05) |
|  |  |  |  |  |  |  |  |  |  | Clinical cure rate | P＜0.05 |  |
|  |  |  |  |  |  |  |  |  |  | Rate of fever recovery | P＜0.05 |  |
|  |  |  |  |  |  |  |  |  |  | Rate of cough recovery | P＜0.05 |  |
|  |  |  |  |  |  |  |  |  |  | Rate of fatigue recovery | P＜0.05 |  |
|  |  |  |  |  |  |  |  |  |  | Time to fever recovery | P＜0.05 |  |
|  |  |  |  |  |  |  |  |  |  | Time to cough recovery | P＜0.05 |  |
|  |  |  |  |  |  |  |  |  |  | Time to fatigue recovery | P＜0.05 |  |
|  |  |  |  |  |  |  |  |  |  | Chest CT manifestations | P＞0.05 |  |
|  |  |  |  |  |  |  |  |  |  | Rate of disease aggravation | P＜0.05 |  |
| Hea Sun Chun,2021 | China | 18 | COVID-19 (NR) | Chinese medicine injection (Xiyanping, Reduning, Tanreqing, Xuebijing, Xingnoajing, Shenfu, Shenmai, and Shengmai), | CMI (Mix 1) | TCM alone | Treatment | Hospitalization | Western medicine | Total Effective Rate | P＞0.05 | No significant difference between the two groups (P＞0.05) |
|  |  |  |  |  |  |  |  |  |  | Time to cough recovery | P＜0.05 |  |
|  |  |  |  |  |  |  |  |  |  | Time to fever recovery | P＜0.05 |  |
|  |  |  |  |  |  |  |  |  |  | Chest CT manifestations | P＜0.05 |  |
| Xuqin Du,2021 | China | 12 | Non-severe COVID-19 | Traditional Chinese medicine | TCM | Integrated Chinese and modern medicines | Treatment | Hospitalization | Western medicine | Chest CT manifestations | P＜0.05 | No significant difference between the two groups (P＞0.05) |
|  |  |  |  |  |  |  |  |  |  | Clinical cure rate | P＜0.05 |  |
|  |  |  |  |  |  |  |  |  |  | Rate of disease aggravation | P＜0.05 |  |
|  |  |  |  |  |  |  |  |  |  | Viral nucleic acid negative conversion rate | P＞0.05 |  |
|  |  |  |  |  |  |  |  |  |  | Rate of fever recovery | P＞0.05 |  |
|  |  |  |  |  |  |  |  |  |  | Rate of cough recovery | P＜0.05 |  |
|  |  |  |  |  |  |  |  |  |  | Rate of fatigue recovery | P＜0.05 |  |
|  |  |  |  |  |  |  |  |  |  | TCM syndrome score | P＜0.05 |  |
|  |  |  |  |  |  |  |  |  |  | WBC | P＜0.05 |  |
|  |  |  |  |  |  |  |  |  |  | LYM | P＜0.05 |  |
|  |  |  |  |  |  |  |  |  |  | CRP | P＜0.05 |  |
| Xuqin Du,2021 | China | 18 | COVID-19 (NR) | Honeysuckle | Honeysuckle | Integrated Chinese and modern medicines | Treatment | Hospitalization | Western medicine | Chest CT manifestations | P＜0.05 | No significant difference between the two groups (P＞0.05) |
|  |  |  |  |  |  |  |  |  |  | Clinical cure rate | P＜0.05 |  |
|  |  |  |  |  |  |  |  |  |  | Viral nucleic acid negative conversion rate | P＞0.05 |  |
|  |  |  |  |  |  |  |  |  |  | Rate of disease aggravation | P＜0.05 |  |
|  |  |  |  |  |  |  |  |  |  | Rate of fever recovery | P＞0.05 |  |
|  |  |  |  |  |  |  |  |  |  | Rate of cough recovery | P＜0.05 |  |
|  |  |  |  |  |  |  |  |  |  | Rate of fatigue recovery | P＞0.05 |  |
|  |  |  |  |  |  |  |  |  |  | TCM syndrome score | P＜0.05 |  |
|  |  |  |  |  |  |  |  |  |  | WBC | P＜0.05 |  |
|  |  |  |  |  |  |  |  |  |  | LYM | P＜0.05 |  |
|  |  |  |  |  |  |  |  |  |  | CRP | P＜0.05 |  |
| Yin Fan | China | 7 | COVID-19 (NR) | Traditional Chinese medicine | TCM | Integrated Chinese and modern medicines | Treatment | Hospitalization | Western medicine | TCM syndrome score | P＜0.05 | No significant difference between the two groups (P＞0.05) |
|  |  |  |  |  |  |  |  |  |  | CRP | P＜0.05 |  |
|  |  |  |  |  |  |  |  |  |  | Chest CT manifestations | P＜0.05 |  |
| Zhihuan Zhou, 2020 | China | 20 | Non-severe COVID-19 | Traditional Chinese medicine | TCM | Integrated Chinese and modern medicines | Treatment | NR | Western medicine | Time to fever recovery | P＜0.05 | NR |
|  |  |  |  |  |  |  |  |  |  | Time to cough recovery | NR |  |
|  |  |  |  |  |  |  |  |  |  | Time to shortness of breath recovery | NR |  |
|  |  |  |  |  |  |  |  |  |  | Chest CT manifestations | NR |  |
|  |  |  |  |  |  |  |  |  |  | CRP | NR |  |
| Caiyun Hu, 2020 | China | 42 | COVID-19 (NR) | Lianhua Qingwen | LHQW | Integrated Chinese and modern medicines | Treatment | NR | Western medicine | Time to fever recovery | P＜0.05 | No significant difference between the two groups (P＞0.05) |
|  |  |  |  |  |  |  |  |  |  | Time to cough recovery | P＜0.05 |  |
|  |  |  |  |  |  |  |  |  |  | Time to sputum recovery | P＜0.05 |  |
|  |  |  |  |  |  |  |  |  |  | Rate of shortness of breath recovery | P＜0.05 |  |
|  |  |  |  |  |  |  |  |  |  | Rate of dyspnea recovery | P＜0.05 |  |
|  |  |  |  |  |  |  |  |  |  | Total Effective Rate | P＜0.05 |  |
|  |  |  |  |  |  |  |  |  |  | Short healing period | P＜0.05 |  |
|  |  |  |  |  |  |  |  |  |  | Rate of fatigue recovery | P＜0.05 |  |
|  |  |  |  |  |  |  |  |  |  | Rate of muscle pain disappearance | P＜0.05 |  |
|  |  |  |  |  |  |  |  |  |  | Rate of sore throat disappearance | P＞0.05 |  |
|  |  |  |  |  |  |  |  |  |  | Rate of nausea/vomiting disappearance | P＞0.05 |  |
|  |  |  |  |  |  |  |  |  |  | Rate of diarrhea disappearance | P＞0.05 |  |
|  |  |  |  |  |  |  |  |  |  | Rate of anorexia disappearance | P＞0.05 |  |
|  |  |  |  |  |  |  |  |  |  | Rate of headache recovery | P＞0.05 |  |
|  |  |  |  |  |  |  |  |  |  | Rate of disease aggravation | P＜0.05 |  |
|  |  |  |  |  |  |  |  |  |  | Rate of chest tightness disappearance | P＜0.05 |  |
| Caiyun Hu2022 | China | 217 | COVID-19 (NR) | Lianhua Qingwen | LHQW | Integrated Chinese and modern medicines | Treatment | NR | Western medicine | NR | NR | No significant difference between the two groups (P＞0.05) |
| Zitong Feng, 2021 | China | 8 | Confirmed or suspected COVID-19 | Traditional Chinese medicine | TCM | Integrated Chinese and modern medicines | Treatment | NR | Western medicine | the duration of loss of smell | NR | No serious adverse events |
|  |  |  |  |  |  |  |  |  |  | the recovery of COVID-19 patients | NR |  |
| Zheng Fan, 2021 | China | 5 | Non-severe COVID-19 | Lianhua Qingwen | LHQW | Integrated Chinese and modern medicines | Treatment | NR | Western medicine | Total Effective Rate | P＜0.05 | No significant difference between the two groups (P＞0.05) |
|  |  |  |  |  |  |  |  |  |  | Chest CT manifestations | P＜0.05 |  |
|  |  |  |  |  |  |  |  |  |  | Rate of disease aggravation | P＜0.05 |  |
|  |  |  |  |  |  |  |  |  |  | Time to fever recovery | P＜0.05 |  |
| Yi Guo, 2020 | China | 44 | COVID-19 (NR) | Chinese medicine injection（Xiyanping, Reduning, Tanreqing） | CMI (Mix 2) | TCM alone | Treatment | NR | Western medicine | Total Effective Rate | P＜0.05 | The control group had a higher incidence（P＜0.05） |
|  |  |  |  |  |  |  |  |  |  | Time to fever recovery | P＜0.05 |  |
|  |  |  |  |  |  |  |  |  |  | Time to pulmonary rales disappearance | P＜0.05 |  |
|  |  |  |  |  |  |  |  |  |  | Time to cough recovery | P＜0.05 |  |
|  |  |  |  |  |  |  |  |  |  | Duration of hospital stay | P＜0.05 |  |
| Ansul Kumar, 2022 | India | 32 | COVID-19 (NR) | Traditional Chinese medicine | TCM | - | Treatment | NR | No restriction | Time to viral assay conversion | P＞0.05 | No significant difference between the two groups (P＞0.05) |
|  |  |  |  |  |  |  |  |  |  | Rate of fever recovery | P＜0.05 |  |
|  |  |  |  |  |  |  |  |  |  | Time to fever recovery | P＜0.05 |  |
|  |  |  |  |  |  |  |  |  |  | Rate of cough recovery | P＜0.05 |  |
|  |  |  |  |  |  |  |  |  |  | Time to cough recovery | P＜0.05 |  |
|  |  |  |  |  |  |  |  |  |  | chest pain | P＞0.05 |  |
|  |  |  |  |  |  |  |  |  |  | Rate of sore throat disappearance | P＞0.05 |  |
|  |  |  |  |  |  |  |  |  |  | Rate of fatigue recovery | P＜0.05 |  |
|  |  |  |  |  |  |  |  |  |  | Time to fatigue recovery | P＜0.05 |  |
|  |  |  |  |  |  |  |  |  |  | Chest CT manifestations | P＜0.05 |  |
|  |  |  |  |  |  |  |  |  |  | Duration of hospital stay | P＞0.05 |  |
|  |  |  |  |  |  |  |  |  |  | Total Effective Rate | P＜0.05 |  |
|  |  |  |  |  |  |  |  |  |  | WBC | P＜0.05 |  |
|  |  |  |  |  |  |  |  |  |  | LYM | P＜0.05 |  |
|  |  |  |  |  |  |  |  |  |  | CRP | P＜0.05 |  |
|  |  |  |  |  |  |  |  |  |  | IL-6 | P＞0.05 |  |
|  |  |  |  |  |  |  |  |  |  | ESR | P＞0.05 |  |
| Fei Jiang, 2021 | China | 35 | COVID-19 (NR) | Traditional Chinese medicine | TCM | Integrated Chinese and modern medicines | Treatment | NR | Western medicine | Rate of disease aggravation | P＜0.05 | No significant difference between the two groups (P＞0.05) |
|  |  |  |  |  |  |  |  |  |  | Viral nucleic acid negative conversion rate | P＞0.05 |  |
|  |  |  |  |  |  |  |  |  |  | Chest CT manifestations | P＜0.05 |  |
|  |  |  |  |  |  |  |  |  |  | Total Effective Rate | P＜0.05 |  |
|  |  |  |  |  |  |  |  |  |  | Rate of fever recovery | P＜0.05 |  |
|  |  |  |  |  |  |  |  |  |  | Time to fever recovery | P＜0.05 |  |
|  |  |  |  |  |  |  |  |  |  | Rate of cough recovery | P＜0.05 |  |
|  |  |  |  |  |  |  |  |  |  | Time to cough recovery | P＜0.05 |  |
|  |  |  |  |  |  |  |  |  |  | Rate of fatigue recovery | P＜0.05 |  |
|  |  |  |  |  |  |  |  |  |  | Time to fatigue recovery | P＜0.05 |  |
|  |  |  |  |  |  |  |  |  |  | WBC | P＜0.05 |  |
|  |  |  |  |  |  |  |  |  |  | LYM | P＜0.05 |  |
|  |  |  |  |  |  |  |  |  |  | Duration of hospital stay | P＜0.05 |  |
| Li, F., 2021 | China | 8 | COVID-19 (NR) | Traditional Chinese medicine | TCM | Integrated Chinese and modern medicines | Treatment | NR | Western medicine | Total Effective Rate | P＜0.05 | NR |
|  |  |  |  |  |  |  |  |  |  | Chest CT manifestations | P＜0.05 |  |
|  |  |  |  |  |  |  |  |  |  | Rate of disease aggravation | P＜0.05 |  |
|  |  |  |  |  |  |  |  |  |  | Rate of fever recovery | P＜0.05 |  |
|  |  |  |  |  |  |  |  |  |  | Rate of fatigue recovery | P＜0.05 |  |
|  |  |  |  |  |  |  |  |  |  | Rate of cough recovery | P＜0.05 |  |
|  |  |  |  |  |  |  |  |  |  | Rate of sputum disappearance | P＜0.05 |  |
|  |  |  |  |  |  |  |  |  |  | Time to fever recovery | P＜0.05 |  |
|  |  |  |  |  |  |  |  |  |  | Time to fatigue recovery | P＜0.05 |  |
|  |  |  |  |  |  |  |  |  |  | Time to cough recovery | P＜0.05 |  |
| Liang, S. B., 2021 | China | 7 | COVID-19 (NR) | Chinese patent medicine | CPM | - | Treatment | NR | Conventional western therapy ± placebo of CPM | Clinical cure rate | P＜0.05 | Unclear |
|  |  |  |  |  |  |  |  |  |  | Rate of disease aggravation | P＜0.05 |  |
|  |  |  |  |  |  |  |  |  |  | Mortality rate | P＞0.05 |  |
|  |  |  |  |  |  |  |  |  |  | Rate of cough recovery | P＜0.05 |  |
|  |  |  |  |  |  |  |  |  |  | Rate of fatigue recovery | P＜0.05 |  |
|  |  |  |  |  |  |  |  |  |  | Rate of fever recovery | P＞0.05 |  |
|  |  |  |  |  |  |  |  |  |  | Time to fever recovery | P＜0.05 |  |
|  |  |  |  |  |  |  |  |  |  | Time to cough recovery | P＜0.05 |  |
|  |  |  |  |  |  |  |  |  |  | Time to fatigue recovery | P＜0.05 |  |
|  |  |  |  |  |  |  |  |  |  | Viral nucleic acid negative conversion rate | P＞0.05 |  |
|  |  |  |  |  |  |  |  |  |  | Chest CT manifestations | P＜0.05 |  |
| Zhuang, J., 2021 | China | 3 | Non-severe COVID-19 | Lianhua Qingwen | LHQW | Integrated Chinese and modern medicines | Treatment | NR | Western medicine | Rate of disease aggravation | P＜0.05 | NR |
|  |  |  |  |  |  |  |  |  |  | Time to fever recovery | P＜0.05 |  |
|  |  |  |  |  |  |  |  |  |  | Rate of fever recovery | P＜0.05 |  |
|  |  |  |  |  |  |  |  |  |  | Rate of cough recovery | P＜0.05 |  |
|  |  |  |  |  |  |  |  |  |  | Rate of fatigue recovery | P＜0.05 |  |
|  |  |  |  |  |  |  |  |  |  | Rate of shortness of breath recovery | P＞0.05 |  |
| Aihua Liu, 2021 | China | 7 | Confirmed or suspected COVID-19 | Traditional Chinese medicine | TCM | - | Treatment | NR | - | Rate of disease aggravation | P＞0.05 | NR |
|  |  |  |  |  |  |  |  |  |  | Total Effective Rate | P＜0.05 |  |
|  |  |  |  |  |  |  |  |  |  | Chest CT manifestations | P＜0.05 |  |
|  |  |  |  |  |  |  |  |  |  | Time to fever recovery | P＜0.05 |  |
|  |  |  |  |  |  |  |  |  |  | Rate of fever recovery | P＜0.05 |  |
|  |  |  |  |  |  |  |  |  |  | Rate of cough recovery | P＜0.05 |  |
|  |  |  |  |  |  |  |  |  |  | Rate of fatigue recovery | P＜0.05 |  |
|  |  |  |  |  |  |  |  |  |  | Rate of sputum disappearance | P＜0.05 |  |
| Lingling Liu, 2021 | China | 7 | COVID-19 (NR) | Traditional Chinese medicine | TCM | Integrated Chinese and modern medicines | Treatment | NR | Western medicine | Total Effective Rate | P＜0.05 | The control group had a higher incidence（P＜0.05） |
|  |  |  |  |  |  |  |  |  |  | TCM syndrome score | P＜0.05 |  |
|  |  |  |  |  |  |  |  |  |  | Rate of fever recovery | P＜0.05 |  |
|  |  |  |  |  |  |  |  |  |  | Rate of cough recovery | P＜0.05 |  |
|  |  |  |  |  |  |  |  |  |  | Rate of fatigue recovery | P＜0.05 |  |
|  |  |  |  |  |  |  |  |  |  | Chest CT manifestations | P＜0.05 |  |
|  |  |  |  |  |  |  |  |  |  | Rate of disease aggravation | P＜0.05 |  |
| Ming Liu, 2020 | China | 11 | COVID-19 (NR) | Traditional Chinese medicine | TCM | Integrated Chinese and modern medicines | Treatment | Hospitalization | Western medicine | Total Effective Rate | P＜0.05 | No significant difference between the two groups (P＞0.05) |
|  |  |  |  |  |  |  |  |  |  | Clinical cure rate | P＜0.05 |  |
|  |  |  |  |  |  |  |  |  |  | Rate of disease aggravation | P＜0.05 |  |
|  |  |  |  |  |  |  |  |  |  | Duration of hospital stay | P＜0.05 |  |
|  |  |  |  |  |  |  |  |  |  | Rate of fever recovery | P＜0.05 |  |
|  |  |  |  |  |  |  |  |  |  | Rate of cough recovery | P＜0.05 |  |
|  |  |  |  |  |  |  |  |  |  | Rate of fatigue recovery | P＜0.05 |  |
|  |  |  |  |  |  |  |  |  |  | Rate of sputum disappearance | P＜0.05 |  |
|  |  |  |  |  |  |  |  |  |  | Rate of chest tightness disappearance | P＜0.05 |  |
|  |  |  |  |  |  |  |  |  |  | Rate of anorexia disappearance | P＜0.05 |  |
|  |  |  |  |  |  |  |  |  |  | Rate of muscle pain disappearance | P＞0.05 |  |
|  |  |  |  |  |  |  |  |  |  | Rate of nausea/vomiting disappearance | P＞0.05 |  |
|  |  |  |  |  |  |  |  |  |  | Rate of diarrhea disappearance | P＞0.05 |  |
|  |  |  |  |  |  |  |  |  |  | Time to fever recovery | P＜0.05 |  |
|  |  |  |  |  |  |  |  |  |  | Time to fatigue recovery | P＜0.05 |  |
|  |  |  |  |  |  |  |  |  |  | CRP | P＜0.05 |  |
|  |  |  |  |  |  |  |  |  |  | WBC | P＜0.05 |  |
|  |  |  |  |  |  |  |  |  |  | TNF-α | P＞0.05 |  |
|  |  |  |  |  |  |  |  |  |  | LYM | P＞0.05 |  |
| Ming Liu, 2021 | China | 8 | COVID-19 (NR) | Lianhua Qingwen | LHQW | Integrated Chinese and modern medicines | Treatment | Hospitalization | Western medicine | Total Effective Rate | P＜0.05 | Unclear |
|  |  |  |  |  |  |  |  |  |  | Symptom recovery rate | P＜0.05 |  |
|  |  |  |  |  |  |  |  |  |  | Rate of diarrhea disappearance | P＜0.05 |  |
|  |  |  |  |  |  |  |  |  |  | Rate of disease aggravation | P＜0.05 |  |
|  |  |  |  |  |  |  |  |  |  | WBC | P＜0.05 |  |
|  |  |  |  |  |  |  |  |  |  | LYM | P＜0.05 |  |
|  |  |  |  |  |  |  |  |  |  | CRP | P＜0.05 |  |
|  |  |  |  |  |  |  |  |  |  | PCT | P＜0.05 |  |
|  |  |  |  |  |  |  |  |  |  | Rate of fever recovery | P＜0.05 |  |
|  |  |  |  |  |  |  |  |  |  | Rate of cough recovery | P＜0.05 |  |
|  |  |  |  |  |  |  |  |  |  | Rate of sputum disappearance | P＜0.05 |  |
|  |  |  |  |  |  |  |  |  |  | Rate of shortness of breath recovery | P＜0.05 |  |
|  |  |  |  |  |  |  |  |  |  | Rate of fatigue recovery | P＜0.05 |  |
|  |  |  |  |  |  |  |  |  |  | Rate of chest tightness disappearance | P＜0.05 |  |
|  |  |  |  |  |  |  |  |  |  | Rate of anorexia disappearance | P＜0.05 |  |
|  |  |  |  |  |  |  |  |  |  | Chest CT manifestations | P＜0.05 |  |
|  |  |  |  |  |  |  |  |  |  | Symptom recovery time | P＜0.05 |  |
| JiaHui Ouyang | China | 10 | Non-severe COVID-19 | Traditional Chinese medicine | TCM | Integrated Chinese and modern medicines | Treatment | Hospitalization | Western medicine | Total Effective Rate | P＜0.05 | No significant difference between the two groups (P＞0.05) |
|  |  |  |  |  |  |  |  |  |  | Time to fever recovery | P＜0.05 |  |
|  |  |  |  |  |  |  |  |  |  | Viral nucleic acid negative conversion rate | P＜0.05 |  |
|  |  |  |  |  |  |  |  |  |  | WBC | P＜0.05 |  |
|  |  |  |  |  |  |  |  |  |  | LYM | P＜0.05 |  |
|  |  |  |  |  |  |  |  |  |  | Chest CT manifestations | P＞0.05 |  |
|  |  |  |  |  |  |  |  |  |  | Rate of fever recovery | P＞0.05 |  |
|  |  |  |  |  |  |  |  |  |  | Rate of cough recovery | P＞0.05 |  |
|  |  |  |  |  |  |  |  |  |  | Rate of fatigue recovery | P＞0.05 |  |
|  |  |  |  |  |  |  |  |  |  | Rate of disease aggravation | P＜0.05 |  |
| Guodong Qi, 2020 | China | 4 | Non-severe COVID-19 | Lianhua Qingwen | LHQW | Integrated Chinese and modern medicines | Treatment | Hospitalization | Western medicine | Total Effective Rate | P＜0.05 | No serious adverse events |
|  |  |  |  |  |  |  |  |  |  | Rate of fever recovery | P＜0.05 |  |
|  |  |  |  |  |  |  |  |  |  | Rate of cough recovery | P＜0.05 |  |
|  |  |  |  |  |  |  |  |  |  | Rate of fatigue recovery | P＜0.05 |  |
|  |  |  |  |  |  |  |  |  |  | Rate of chest tightness disappearance | P＜0.05 |  |
|  |  |  |  |  |  |  |  |  |  | Rate of anorexia disappearance | P＜0.05 |  |
|  |  |  |  |  |  |  |  |  |  | Rate of sputum disappearance | P＜0.05 |  |
| Fangfang Zhou, 2021 | China | 6 | COVID-19 (NR) | Chinese herbal decoction | CHD | Integrated Chinese and modern medicines | Treatment | Hospitalization | Western medicine | Clinical cure rate | P＞0.05 | No significant difference between the two groups (P＞0.05) |
|  |  |  |  |  |  |  |  |  |  | Total Effective Rate | P＞0.05 |  |
|  |  |  |  |  |  |  |  |  |  | Mortality rate | P＞0.05 |  |
|  |  |  |  |  |  |  |  |  |  | Duration of hospital stay | P＜0.05 |  |
|  |  |  |  |  |  |  |  |  |  | Rate of cough recovery | P＜0.05 |  |
|  |  |  |  |  |  |  |  |  |  | Rate of fatigue recovery | P＜0.05 |  |
|  |  |  |  |  |  |  |  |  |  | Rate of fever recovery | P＞0.05 |  |
|  |  |  |  |  |  |  |  |  |  | Rate of shortness of breath recovery | P＞0.05 |  |
|  |  |  |  |  |  |  |  |  |  | Chest CT manifestations | P＞0.05 |  |
| Xufei Luo, 2021 | China | 19 | COVID-19 (NR) | Traditional Chinese medicine | TCM | Integrated Chinese and modern medicines | Treatment | NR | Western medicine | Total Effective Rate | P＜0.05 | No significant difference between the two groups (P＞0.05) |
|  |  |  |  |  |  |  |  |  |  | Chest CT manifestations | P＜0.05 |  |
|  |  |  |  |  |  |  |  |  |  | Rate of disease aggravation | P＜0.05 |  |
|  |  |  |  |  |  |  |  |  |  | Viral nucleic acid negative conversion rate | P＜0.05 |  |
|  |  |  |  |  |  |  |  |  |  | Rate of fever recovery | P＜0.05 |  |
|  |  |  |  |  |  |  |  |  |  | Rate of cough recovery | P＜0.05 |  |
|  |  |  |  |  |  |  |  |  |  | Rate of fatigue recovery | P＜0.05 |  |
|  |  |  |  |  |  |  |  |  |  | Duration of hospital stay | P＞0.05 |  |
| Yanli Tang, 2021 | China | 5 | COVID-19 (NR) | Lianhua Qingwen | LHQW | Integrated Chinese and modern medicines | Treatment | NR | Western medicine | Rate of fever recovery | P＜0.05 | Unclear |
|  |  |  |  |  |  |  |  |  |  | Time to fever recovery | P＞0.05 |  |
|  |  |  |  |  |  |  |  |  |  | Rate of cough recovery | P＜0.05 |  |
|  |  |  |  |  |  |  |  |  |  | Time to cough recovery | P＜0.05 |  |
|  |  |  |  |  |  |  |  |  |  | Rate of fatigue recovery | P＜0.05 |  |
|  |  |  |  |  |  |  |  |  |  | Time to fatigue recovery | P＜0.05 |  |
|  |  |  |  |  |  |  |  |  |  | Total Effective Rate | P＜0.05 |  |
|  |  |  |  |  |  |  |  |  |  | Chest CT manifestations | P＜0.05 |  |
|  |  |  |  |  |  |  |  |  |  | Rate of disease aggravation | P＜0.05 |  |
|  |  |  |  |  |  |  |  |  |  | WBC | P＜0.05 |  |
|  |  |  |  |  |  |  |  |  |  | LYM | P＜0.05 |  |
|  |  |  |  |  |  |  |  |  |  | CRP | P＜0.05 |  |
|  |  |  |  |  |  |  |  |  |  | PCT | P＜0.05 |  |
| Shihua Shi, 2021 | China | 48 | COVID-19 (NR) | Traditional Chinese medicine | TCM | Integrated Chinese and modern medicines | Treatment | NR | Western medicine | Rate of nausea/vomiting disappearance | P＞0.05 | NR |
|  |  |  |  |  |  |  |  |  |  | Rate of nausea/vomiting disappearance | P＞0.05 |  |
|  |  |  |  |  |  |  |  |  |  | Rate of anorexia disappearance | P＜0.05 |  |
|  |  |  |  |  |  |  |  |  |  | Rate of diarrhea disappearance | P＞0.05 |  |
|  |  |  |  |  |  |  |  |  |  | The recovery of liver function | P＞0.05 |  |
|  |  |  |  |  |  |  |  |  |  | Rate of disease aggravation | P＜0.05 |  |
|  |  |  |  |  |  |  |  |  |  | Time to viral assay conversion | P＜0.05 |  |
| Shihua Shi, 2021 | China | 30 | COVID-19 (NR) | Traditional Chinese medicine | TCM | Integrated Chinese and modern medicines | Treatment | NR | Western medicine | LYM | P＜0.05 | No significant difference between the two groups (P＞0.05) |
|  |  |  |  |  |  |  |  |  |  | CD4+ improvement | P＜0.05 |  |
|  |  |  |  |  |  |  |  |  |  | CD8+ improvement | P＞0.05 |  |
|  |  |  |  |  |  |  |  |  |  | CD4+/CD8+ Ratio improvement | P＜0.05 |  |
|  |  |  |  |  |  |  |  |  |  | CD3+ improvement | P＜0.05 |  |
|  |  |  |  |  |  |  |  |  |  | WBC | P＞0.05 |  |
|  |  |  |  |  |  |  |  |  |  | TNF-α | P＜0.05 |  |
|  |  |  |  |  |  |  |  |  |  | IL-6 | P＞0.05 |  |
|  |  |  |  |  |  |  |  |  |  | Mortality rate | P＜0.05 |  |
|  |  |  |  |  |  |  |  |  |  | Time to fever recovery | P＜0.05 |  |
|  |  |  |  |  |  |  |  |  |  | Time to cough recovery | P＜0.05 |  |
|  |  |  |  |  |  |  |  |  |  | Time to the Remission of Chest Tightness | P＜0.05 |  |
|  |  |  |  |  |  |  |  |  |  | Time to fatigue recovery | P＜0.05 |  |
| Chengqian Shi, 2022 | China | 5 | Non-severe COVID-19 | Lianhua Qingwen | LHQW | Integrated Chinese and modern medicines | Treatment | NR | Western medicine | Total Effective Rate | P＜0.05 | NR |
|  |  |  |  |  |  |  |  |  |  | Chest CT manifestations | P＜0.05 |  |
|  |  |  |  |  |  |  |  |  |  | Rate of disease aggravation | P＜0.05 |  |
| Wentai Pang, 2020 | China | 11 | COVID-19 (NR) | Traditional Chinese medicine | TCM | Integrated Chinese and modern medicines | Treatment | NR | Western medicine | Rate of disease aggravation | P＜0.05 | No significant difference between the two groups (P＞0.05) |
|  |  |  |  |  |  |  |  |  |  | Mortality rate | P＞0.05 |  |
|  |  |  |  |  |  |  |  |  |  | Duration of hospital stay | P＜0.05 |  |
|  |  |  |  |  |  |  |  |  |  | Viral nucleic acid negative conversion rate | P＞0.05 |  |
|  |  |  |  |  |  |  |  |  |  | clinical symptom score | P＞0.05 |  |
|  |  |  |  |  |  |  |  |  |  | Time to fever recovery | P＜0.05 |  |
|  |  |  |  |  |  |  |  |  |  | Rate of fever recovery | P＞0.05 |  |
|  |  |  |  |  |  |  |  |  |  | Time to cough recovery | P＞0.05 |  |
|  |  |  |  |  |  |  |  |  |  | Rate of cough recovery | P＜0.05 |  |
|  |  |  |  |  |  |  |  |  |  | Time to fatigue recovery | P＞0.05 |  |
|  |  |  |  |  |  |  |  |  |  | Rate of fatigue recovery | P＜0.05 |  |
|  |  |  |  |  |  |  |  |  |  | Rate of shortness of breath recovery | P＜0.05 |  |
|  |  |  |  |  |  |  |  |  |  | Rate of diarrhea disappearance | P＞0.05 |  |
|  |  |  |  |  |  |  |  |  |  | Rate of muscle pain disappearance | P＞0.05 |  |
| Qi Wang, 2021 | China | 16 | COVID-19 (NR) | Qingfei Paidu Decoction | QFPD | Integrated Chinese and modern medicines | Treatment | NR | Western medicine | Time to viral assay conversion | P＜0.05 | No serious adverse events |
|  |  |  |  |  |  |  |  |  |  | Duration of hospital stay | P＜0.05 |  |
|  |  |  |  |  |  |  |  |  |  | TCM syndrome score | P＜0.05 |  |
|  |  |  |  |  |  |  |  |  |  | Time to fever recovery | P＜0.05 |  |
|  |  |  |  |  |  |  |  |  |  | Time to cough recovery | P＜0.05 |  |
|  |  |  |  |  |  |  |  |  |  | Total Effective Rate | P＜0.05 |  |
|  |  |  |  |  |  |  |  |  |  | WBC | P＜0.05 |  |
|  |  |  |  |  |  |  |  |  |  | CRP | P＜0.05 |  |
|  |  |  |  |  |  |  |  |  |  | ESR | P＜0.05 |  |
|  |  |  |  |  |  |  |  |  |  | laboratory indexes: WBC；NEUT；CRP； LYMPH； ESR ； DD ；ALT； AST ; PCO2; PO2; CD3; CD4; and CD8 | P＜0.05 |  |
|  |  |  |  |  |  |  |  |  |  | Chest CT manifestations | P＜0.05 |  |
| Heping Wang,2021 | China | 25 | COVID-19 (Mix) | Traditional Chinese medicine | TCM | Integrated Chinese and modern medicines | Treatment | NR | Western medicine | Clinical cure rate | P＜0.05 | No significant difference between the two groups (P＞0.05) |
|  |  |  |  |  |  |  |  |  |  | Viral nucleic acid negative conversion rate | P＞0.05 |  |
|  |  |  |  |  |  |  |  |  |  | (ARDS) incidence of unfavorable clinical events of acute respiratory distress syndrome | P＜0.05 |  |
|  |  |  |  |  |  |  |  |  |  | Forest plot of mechanical ventilation. | P＜0.05 |  |
|  |  |  |  |  |  |  |  |  |  | Rate of disease aggravation | P＜0.05 |  |
|  |  |  |  |  |  |  |  |  |  | Time to fever recovery | P＜0.05 |  |
|  |  |  |  |  |  |  |  |  |  | Mortality rate | P＜0.05 |  |
|  |  |  |  |  |  |  |  |  |  | Duration of hospital stay | NR |  |
|  |  |  |  |  |  |  |  |  |  | Chest CT manifestations | P＜0.05 |  |
| Deng-chao Wang, 2021 | China | 6 | COVID-19 (NR) | Lianhua Qingwen | LHQW | Integrated Chinese and modern medicines | Treatment | Hospitalization | Western medicine | Time to fever recovery | P＜0.05 | No significant difference between the two groups (P＞0.05) |
|  |  |  |  |  |  |  |  |  |  | Rate of fever recovery | P＜0.05 |  |
|  |  |  |  |  |  |  |  |  |  | Rate of cough recovery | P＜0.05 |  |
|  |  |  |  |  |  |  |  |  |  | Rate of shortness of breath recovery | P＜0.05 |  |
|  |  |  |  |  |  |  |  |  |  | Rate of fatigue recovery | P＜0.05 |  |
|  |  |  |  |  |  |  |  |  |  | Total Effective Rate | P＜0.05 |  |
| Chun-Yang Sun, 2020 | China | 7 | COVID-19 (NR) | Traditional Chinese medicine | TCM | Integrated Chinese and modern medicines | Treatment | NR | Western medicine | Total Effective Rate | P＜0.05 | No significant difference between the two groups (P＞0.05) |
|  |  |  |  |  |  |  |  |  |  | Viral nucleic acid negative conversion rate | P＜0.05 |  |
|  |  |  |  |  |  |  |  |  |  | Chest CT manifestations | P＜0.05 |  |
|  |  |  |  |  |  |  |  |  |  | WBC | P＜0.05 |  |
|  |  |  |  |  |  |  |  |  |  | LYM | P＜0.05 |  |
|  |  |  |  |  |  |  |  |  |  | CRP | P＜0.05 |  |
|  |  |  |  |  |  |  |  |  |  | IL-6 | P＜0.05 |  |
| Shuxia Wang, 2020 | China | 7 | COVID-19 (NR) | Lianhua Qingwen | LHQW | Integrated Chinese and modern medicines | Treatment | Hospitalization | Western medicine | Total Effective Rate | P＜0.05 | NR |
|  |  |  |  |  |  |  |  |  |  | Chest CT manifestations | P＜0.05 |  |
|  |  |  |  |  |  |  |  |  |  | Rate of disease aggravation | P＜0.05 |  |
|  |  |  |  |  |  |  |  |  |  | Time to fever recovery | P＜0.05 |  |
|  |  |  |  |  |  |  |  |  |  | Time to clinical symptoms recovery | P＜0.05 |  |
|  |  |  |  |  |  |  |  |  |  | Duration of hospital stay | P＜0.05 |  |
| Qianfei Wang, 2021 | China | 13 | COVID-19 (NR) | Traditional Chinese medicine | TCM | Integrated Chinese and modern medicines | Treatment | Hospitalization | Western medicine | Total Effective Rate | P＜0.05 | No serious adverse events |
|  |  |  |  |  |  |  |  |  |  | Rate of fever recovery | P＜0.05 |  |
|  |  |  |  |  |  |  |  |  |  | Chest CT manifestations | P＜0.05 |  |
|  |  |  |  |  |  |  |  |  |  | LYM | P＜0.05 |  |
|  |  |  |  |  |  |  |  |  |  | CRP | P＜0.05 |  |
| Xiaozheng Wu, 2021 | China | 15 | Non-severe COVID-19 | Traditional Chinese medicine | TCM | Integrated Chinese and modern medicines | Treatment | NR | Western medicine | Total Effective Rate | P＜0.05 | No significant difference between the two groups (P＞0.05) |
|  |  |  |  |  |  |  |  |  |  | Rate of fever recovery | P＜0.05 |  |
|  |  |  |  |  |  |  |  |  |  | Time to fever recovery | P＜0.05 |  |
|  |  |  |  |  |  |  |  |  |  | Rate of cough recovery | P＜0.05 |  |
|  |  |  |  |  |  |  |  |  |  | Time to cough recovery | P＜0.05 |  |
|  |  |  |  |  |  |  |  |  |  | Rate of fatigue recovery | P＜0.05 |  |
|  |  |  |  |  |  |  |  |  |  | Time to fatigue recovery | P＜0.05 |  |
|  |  |  |  |  |  |  |  |  |  | Chest CT manifestations | P＜0.05 |  |
|  |  |  |  |  |  |  |  |  |  | Rate of disease aggravation | P＜0.05 |  |
|  |  |  |  |  |  |  |  |  |  | Viral nucleic acid negative conversion rate | P＞0.05 |  |
| Yangzihan Wang, 2022 | UK | 13 | Confirmed or suspected COVID-19 | Traditional Chinese medicine | TCM | Integrated Chinese and modern medicines | Treatment | NR | Western medicine | Rate of disease aggravation | NR | No serious adverse events |
|  |  |  |  |  |  |  |  |  |  | Time to fever recovery | P＜0.05 |  |
|  |  |  |  |  |  |  |  |  |  | Rate of fever recovery | P＜0.05 |  |
|  |  |  |  |  |  |  |  |  |  | Rate of cough recovery | P＜0.05 |  |
|  |  |  |  |  |  |  |  |  |  | Rate of fatigue recovery | P＜0.05 |  |
|  |  |  |  |  |  |  |  |  |  | Rate of sputum disappearance | P＜0.05 |  |
|  |  |  |  |  |  |  |  |  |  | Rate of shortness of breath recovery | P＜0.05 |  |
|  |  |  |  |  |  |  |  |  |  | Rate of muscle pain disappearance | P＜0.05 |  |
|  |  |  |  |  |  |  |  |  |  | Rate of chest tightness disappearance | P＞0.05 |  |
|  |  |  |  |  |  |  |  |  |  | Rate of diarrhea disappearance | P＞0.05 |  |
|  |  |  |  |  |  |  |  |  |  | Rate of nausea/vomiting disappearance | P＞0.05 |  |
|  |  |  |  |  |  |  |  |  |  | Rate of anorexia disappearance | P＞0.05 |  |
|  |  |  |  |  |  |  |  |  |  | Rate of sore throat disappearance | P＞0.05 |  |
|  |  |  |  |  |  |  |  |  |  | Rate of headache recovery | P＞0.05 |  |
|  |  |  |  |  |  |  |  |  |  | Rate of nasal congestion/runny nose recovery | P＞0.05 |  |
|  |  |  |  |  |  |  |  |  |  | Chest CT manifestations | P＜0.05 |  |
| Lizhao Yan, 2021 | China | 13 | COVID-19 (NR) | Traditional Chinese medicine | TCM | Integrated Chinese and modern medicines | Treatment | Hospitalization | Western medicine | Rate of disease aggravation | P＜0.05 | NR |
|  |  |  |  |  |  |  |  |  |  | Clinical cure rate | P＜0.05 |  |
|  |  |  |  |  |  |  |  |  |  | Mortality rate | P＞0.05 |  |
|  |  |  |  |  |  |  |  |  |  | Rate of cough recovery | P＞0.05 |  |
|  |  |  |  |  |  |  |  |  |  | Rate of diarrhea disappearance | P＞0.05 |  |
|  |  |  |  |  |  |  |  |  |  | Chest CT manifestations | P＞0.05 |  |
|  |  |  |  |  |  |  |  |  |  | Time to fever recovery | P＜0.05 |  |
|  |  |  |  |  |  |  |  |  |  | Time to cough recovery | P＞0.05 |  |
|  |  |  |  |  |  |  |  |  |  | Time to rhinobyon remission | P＞0.05 |  |
|  |  |  |  |  |  |  |  |  |  | the coryza remission time | P＞0.05 |  |
|  |  |  |  |  |  |  |  |  |  | Time to fatigue recovery | P＜0.05 |  |
|  |  |  |  |  |  |  |  |  |  | Duration of hospital stay | P＜0.05 |  |
|  |  |  |  |  |  |  |  |  |  | the disappearance rate of fever | P＜0.05 |  |
|  |  |  |  |  |  |  |  |  |  | the disappearance rate of exhaustion | P＜0.05 |  |
|  |  |  |  |  |  |  |  |  |  | the disappearance rate of anorexia | P＞0.05 |  |
|  |  |  |  |  |  |  |  |  |  | the disappearance rate of sore throat | P＞0.05 |  |
|  |  |  |  |  |  |  |  |  |  | Rate of fever recovery | P＜0.05 |  |
|  |  |  |  |  |  |  |  |  |  | Rate of nasal congestion/runny nose recovery | P＞0.05 |  |
|  |  |  |  |  |  |  |  |  |  | the disappearance rate of coryza | P＞0.05 |  |
|  |  |  |  |  |  |  |  |  |  | Rate of sore throat disappearance | P＞0.05 |  |
|  |  |  |  |  |  |  |  |  |  | Rate of fatigue recovery | P＜0.05 |  |
|  |  |  |  |  |  |  |  |  |  | Rate of headache recovery | P＞0.05 |  |
|  |  |  |  |  |  |  |  |  |  | Rate of nausea/vomiting disappearance | P＞0.05 |  |
|  |  |  |  |  |  |  |  |  |  | Rate of nausea/vomiting disappearance | P＞0.05 |  |
| Xingjiang Xiong, 2020 | China | 18 | COVID-19 (NR) | Traditional Chinese medicine | TCM | Integrated Chinese and modern medicines | Treatment | Hospitalization | Western medicine | Chest CT manifestations | P＜0.05 | No significant difference between the two groups (P＞0.05) |
|  |  |  |  |  |  |  |  |  |  | Mortality rate | P＞0.05 |  |
|  |  |  |  |  |  |  |  |  |  | Clinical cure rate | P＜0.05 |  |
|  |  |  |  |  |  |  |  |  |  | the effects of CHM on ranging between mild and critical cases | P＞0.05 |  |
|  |  |  |  |  |  |  |  |  |  | Rate of disease aggravation | P＜0.05 |  |
|  |  |  |  |  |  |  |  |  |  | Duration of hospital stay | P＜0.05 |  |
|  |  |  |  |  |  |  |  |  |  | total score of clinical symptoms | P＜0.05 |  |
|  |  |  |  |  |  |  |  |  |  | Rate of fever recovery | P＞0.05 |  |
|  |  |  |  |  |  |  |  |  |  | Time to fever recovery | P＜0.05 |  |
|  |  |  |  |  |  |  |  |  |  | symptom score of fever | P＜0.05 |  |
|  |  |  |  |  |  |  |  |  |  | Rate of cough recovery | P＜0.05 |  |
|  |  |  |  |  |  |  |  |  |  | symptom score of cough | P＜0.05 |  |
|  |  |  |  |  |  |  |  |  |  | Time to cough recovery | P＜0.05 |  |
|  |  |  |  |  |  |  |  |  |  | Rate of fatigue recovery | P＜0.05 |  |
|  |  |  |  |  |  |  |  |  |  | symptom score of fatigue | P＜0.05 |  |
|  |  |  |  |  |  |  |  |  |  | Time to fatigue recovery | P＜0.05 |  |
|  |  |  |  |  |  |  |  |  |  | TCM syndrome score | P＜0.05 |  |
|  |  |  |  |  |  |  |  |  |  | Viral nucleic acid negative conversion rate | P＜0.05 |  |
|  |  |  |  |  |  |  |  |  |  | WBC | P＞0.05 |  |
|  |  |  |  |  |  |  |  |  |  | NEU | NR |  |
|  |  |  |  |  |  |  |  |  |  | LYM | P＞0.05 |  |
|  |  |  |  |  |  |  |  |  |  | CRP | P＜0.05 |  |
| Huiyue Zhang, 2020 | China | 5 | Suspected COVID-19 | Lianhua Qingwen | LHQW | Integrated Chinese and modern medicines | Treatment | NR | Western medicine | Rate of fever recovery | P＜0.05 | Unclear |
|  |  |  |  |  |  |  |  |  |  | Rate of cough recovery | P＜0.05 |  |
|  |  |  |  |  |  |  |  |  |  | Rate of fatigue recovery | P＜0.05 |  |
|  |  |  |  |  |  |  |  |  |  | Rate of disease aggravation | P＜0.05 |  |
| Meng Yang,2020 | China | 3 | Non-severe COVID-19 | Lianhua Qingwen | LHQW | Integrated Chinese and modern medicines | Treatment | Hospitalization | Western medicine | Rate of fever recovery | P＜0.05 | No significant difference between the two groups (P＞0.05) |
|  |  |  |  |  |  |  |  |  |  | Rate of cough recovery | P＜0.05 |  |
|  |  |  |  |  |  |  |  |  |  | Rate of fatigue recovery | P＜0.05 |  |
|  |  |  |  |  |  |  |  |  |  | Rate of chest tightness disappearance | P＞0.05 |  |
|  |  |  |  |  |  |  |  |  |  | Rate of dyspnea recovery | P＜0.05 |  |
|  |  |  |  |  |  |  |  |  |  | Rate of anorexia disappearance | P＜0.05 |  |
| Guang Yang, 2021 | China | 7 | COVID-19 (Mix) | Chinese medicine injection | CMI (NS) | Integrated Chinese and modern medicines | Treatment | Hospitalization | Western medicine | Time to viral assay conversion | P＜0.05 | No significant difference between the two groups (P＞0.05) |
|  |  |  |  |  |  |  |  |  |  | Duration of hospital stay | P＜0.05 |  |
|  |  |  |  |  |  |  |  |  |  | APACHE II | P＜0.05 |  |
|  |  |  |  |  |  |  |  |  |  | Chest CT manifestations | P＜0.05 |  |
|  |  |  |  |  |  |  |  |  |  | LYM | P＜0.05 |  |
|  |  |  |  |  |  |  |  |  |  | IL-6 | P＜0.05 |  |
|  |  |  |  |  |  |  |  |  |  | Total Effective Rate | P＜0.05 |  |
|  |  |  |  |  |  |  |  |  |  | Time to viral assay conversion | P＞0.05 |  |
|  |  |  |  |  |  |  |  |  |  | Duration of hospital stay | P＞0.05 |  |
|  |  |  |  |  |  |  |  |  |  | APACHE II | P＞0.05 |  |
|  |  |  |  |  |  |  |  |  |  | Chest CT manifestations | P＞0.05 |  |
|  |  |  |  |  |  |  |  |  |  | LYM | P＞0.05 |  |
|  |  |  |  |  |  |  |  |  |  | IL-6 | P＞0.05 |  |
|  |  |  |  |  |  |  |  |  |  | Total Effective Rate | P＞0.05 |  |
| Mengjie Zeng，2020 | China | 2 | COVID-19 (NR) | Lianhua Qingwen | LHQW | Integrated Chinese and modern medicines | Treatment | Hospitalization | Western medicine | Rate of fever recovery | P＜0.05 | NR |
|  |  |  |  |  |  |  |  |  |  | Rate of cough recovery | P＜0.05 |  |
|  |  |  |  |  |  |  |  |  |  | Rate of fatigue recovery | P＜0.05 |  |
|  |  |  |  |  |  |  |  |  |  | Rate of muscle pain disappearance | P＜0.05 |  |
|  |  |  |  |  |  |  |  |  |  | Rate of sputum disappearance | P＜0.05 |  |
|  |  |  |  |  |  |  |  |  |  | Rate of shortness of breath recovery | P＜0.05 |  |
|  |  |  |  |  |  |  |  |  |  | Rate of chest tightness disappearance | P＜0.05 |  |
|  |  |  |  |  |  |  |  |  |  | Rate of dyspnea recovery | P＞0.05 |  |
|  |  |  |  |  |  |  |  |  |  | Rate of nausea/vomiting disappearance | P＞0.05 |  |
|  |  |  |  |  |  |  |  |  |  | Rate of anorexia disappearance | P＞0.05 |  |
|  |  |  |  |  |  |  |  |  |  | Time to fever recovery | P＜0.05 |  |
| Ruizhe Yu, 2022 | China | 16 | COVID-19 (NR) | Traditional Chinese medicine | TCM | Integrated Chinese and modern medicines | Treatment | Hospitalization | Western medicine | Clinical cure rate | P＜0.05 | NR |
|  |  |  |  |  |  |  |  |  |  | Rate of disease aggravation | P＜0.05 |  |
|  |  |  |  |  |  |  |  |  |  | Total Effective Rate | P＜0.05 |  |
|  |  |  |  |  |  |  |  |  |  | Rate of fever recovery | P＜0.05 |  |
|  |  |  |  |  |  |  |  |  |  | Time to fever recovery | P＜0.05 |  |
|  |  |  |  |  |  |  |  |  |  | Rate of cough recovery | P＜0.05 |  |
|  |  |  |  |  |  |  |  |  |  | Time to cough recovery | P＜0.05 |  |
|  |  |  |  |  |  |  |  |  |  | Rate of fatigue recovery | P＜0.05 |  |
|  |  |  |  |  |  |  |  |  |  | Time to fatigue recovery | P＜0.05 |  |
|  |  |  |  |  |  |  |  |  |  | Chest CT manifestations | P＜0.05 |  |
|  |  |  |  |  |  |  |  |  |  | Viral nucleic acid negative conversion rate | P＞0.05 |  |
|  |  |  |  |  |  |  |  |  |  | WBC | P＜0.05 |  |
|  |  |  |  |  |  |  |  |  |  | LYM | P＜0.05 |  |
| Bei Yin, 2021 | China | 19 | COVID-19 (NR) | Traditional Chinese medicine | TCM | Integrated Chinese and modern medicines | Treatment | Hospitalization | Western medicine | Total Effective Rate | P＜0.05 | NR |
|  |  |  |  |  |  |  |  |  |  | Rate of fever recovery | P＜0.05 |  |
|  |  |  |  |  |  |  |  |  |  | Rate of cough recovery | P＜0.05 |  |
|  |  |  |  |  |  |  |  |  |  | Chest CT manifestations | P＜0.05 |  |
|  |  |  |  |  |  |  |  |  |  | CRP | P＜0.05 |  |
|  |  |  |  |  |  |  |  |  |  | WBC | P＜0.05 |  |
| Qinxin Zheng, 2021 | China | 22 | COVID-19 (NR) | Traditional Chinese medicine | TCM | Integrated Chinese and modern medicines | Treatment | Hospitalization | Western medicine | Total Effective Rate | P＜0.05 | No significant difference between the two groups (P＞0.05) |
|  |  |  |  |  |  |  |  |  |  | Rate of disease aggravation | P＜0.05 |  |
|  |  |  |  |  |  |  |  |  |  | Mortality rate | P＜0.05 |  |
|  |  |  |  |  |  |  |  |  |  | Time to cough recovery | P＜0.05 |  |
|  |  |  |  |  |  |  |  |  |  | Time to fever recovery | P＜0.05 |  |
|  |  |  |  |  |  |  |  |  |  | Time to viral assay conversion | P＜0.05 |  |
|  |  |  |  |  |  |  |  |  |  | Duration of hospital stay | P＜0.05 |  |
| Wenbin Zhang, 2020 | China | 5 | Non-severe COVID-19 | Lianhua Qingwen | LHQW | Integrated Chinese and modern medicines | Treatment | Hospitalization | Western medicine | Total Effective Rate | P＜0.05 | The control group had a higher incidence（P＜0.05） |
|  |  |  |  |  |  |  |  |  |  | Rate of fever recovery | P＜0.05 |  |
|  |  |  |  |  |  |  |  |  |  | Rate of cough recovery | P＜0.05 |  |
|  |  |  |  |  |  |  |  |  |  | Rate of fatigue recovery | P＜0.05 |  |
|  |  |  |  |  |  |  |  |  |  | Rate of shortness of breath recovery | P＜0.05 |  |
|  |  |  |  |  |  |  |  |  |  | Time to fever recovery | P＜0.05 |  |
|  |  |  |  |  |  |  |  |  |  | Rate of disease aggravation | P＜0.05 |  |
| Lepeng Zhou, 2020 | China | 10 | COVID-19 (NR) | Traditional Chinese medicine | TCM | Integrated Chinese and modern medicines | Treatment | Hospitalization | Western medicine | Clinical cure rate | P＜0.05 | Unclear |
|  |  |  |  |  |  |  |  |  |  | Rate of fever recovery | P＞0.05 |  |
|  |  |  |  |  |  |  |  |  |  | Rate of cough recovery | P＜0.05 |  |
|  |  |  |  |  |  |  |  |  |  | Chest CT manifestations | P＜0.05 |  |
|  |  |  |  |  |  |  |  |  |  | Rate of disease aggravation | P＜0.05 |  |
| Yuqin, Wu, 2020 | China | 8 | COVID-19 (NR) | Traditional Chinese medicine | TCM | Integrated Chinese and modern medicines | Treatment | NR | Western medicine | Rate of disease aggravation | P＜0.05 | NR |
|  |  |  |  |  |  |  |  |  |  | Clinical cure rate | P＜0.05 |  |
|  |  |  |  |  |  |  |  |  |  | Mortality rate | P＞0.05 |  |
|  |  |  |  |  |  |  |  |  |  | Time to fever recovery | P＜0.05 |  |
|  |  |  |  |  |  |  |  |  |  | Symptom score | P＜0.05 |  |
|  |  |  |  |  |  |  |  |  |  | Rate of cough recovery | P＜0.05 |  |
|  |  |  |  |  |  |  |  |  |  | Rate of diarrhea disappearance | P＞0.05 |  |
|  |  |  |  |  |  |  |  |  |  | Chest CT manifestations | P＞0.05 |  |

# Table S5. Risk of bias assessment for RCTs

| **Author, year** | **Country** | **Random sequence generation** | **Allocation concealment** | **Blinding of participants and personnel** | **Blinding of outcome assessment** | **Incomplete outcome data** | **Selective reporting** | **Other bias** |
| --- | --- | --- | --- | --- | --- | --- | --- | --- |
| Xuedong An, 2021 | China | Low | Unclear | Unclear | Low | Low | High | Low |
| Xiangying Ai, 2020 | China | Low | Unclear | Unclear | Low | Low | Low | Low |
| Wenming Ban, 2021 | China | Low | Unclear | Unclear | Low | Low | Low | Low |
| Kequn Chai, 2021 | China | Unclear | Unclear | Unclear | Low | Low | Low | Low |
| Zizhou Zheng, 2020 | China | Unclear | Unclear | Unclear | Low | Low | Low | Low |
| chaowu chen,2021 | China | Low | High | Unclear | Unclear | Low | Low | Low |
| erhui chen, 2020 | China | High | High | High | Unclear | Low | Low | Low |
| liangzhong chen, 2020 | China | Low | Unclear | Unclear | Unclear | Low | Low | Low |
| suxin chen | China | Low | High | High | Unclear | Low | Low | Low |
| Yuqin Chen, 2021 | China | Low | Low | Low | Low | Low | Low | Low |
| Shuang Zhou，2021 | China | Low | Unclear | Unclear | Unclear | Low | Low | Low |
| Fataneh Hashem-Dabaghian, 2021 | Iran | Low | Unclear | Unclear | Unclear | Low | Low | Low |
| Qin He, 2021 | China | Low | Unclear | Unclear | Unclear | Low | Low | Unclear |
| Qin He, 2020 | China | Low | Unclear | Unclear | Unclear | Low | Low | Unclear |
| Can Duan, 2020 | China | Low | Unclear | Unclear | Unclear | Low | Low | Low |
| Ke Hu, 2021 | China | Low | Unclear | Unclear | Unclear | Low | Low | Low |
| Lulu Li, 2021 | China | Low | Unclear | Unclear | Unclear | Low | Low | Low |
| Lan Li, 2021 | China | Low | Unclear | Unclear | Unclear | Low | Low | Low |
| Wenxue Ju, 2022 | China | Low | Unclear | Unclear | Unclear | Low | Low | Low |
| Wei Jin, 2020 | China | Low | Unclear | Unclear | Unclear | Low | Low | Low |
| Li, L., 2021 | China | Low | Unclear | Low | Unclear | Low | Unclear | Low |
| Guorong Liao, 2020 | China | Unclear | Unclear | Unclear | Unclear | Low | Low | Unclear |
| Fan Liu, 2020 | China | Unclear | Unclear | Unclear | Unclear | Low | Low | Unclear |
| Wu Liu, 2021 | China | Low | Unclear | Unclear | Unclear | Low | Low | Unclear |
| Jia Liu, 2021 | China | Low | Low | Unclear | Low | Low | Low | Low |
| Yuanyuan Meng, 2020 | China | Unclear | Unclear | Unclear | Unclear | Low | Low | Low |
| Min Qiu, 2020 | China | Low | Unclear | Unclear | Unclear | Low | Low | Low |
| Qiuyue Shi, 2020 | China | Unclear | Unclear | Unclear | Unclear | Low | Low | Low |
| Li Ni, 2021 | China | Low | Unclear | Unclear | Low | Low | Low | Low |
| Mesri, M., 2021 | Iran | Low | Low | Low | Low | Low | Low | Low |
| Qinhai Ma, 2021 | China | High | Unclear | Unclear | Unclear | Low | Low | Low |
| Huimin Sun, 2020 | China | Low | Unclear | High | High | Low | Low | Unclear |
| Dandan Song, 2021 | China | Low | Unclear | High | Unclear | Low | Low | Low |
| Suofang Shi 1, 2020 | China | Low | Unclear | Unclear | Unclear | Low | Low | Low |
| Suofang Shi 2, 2020 | China | Unclear | Unclear | Unclear | Unclear | Low | Low | Low |
| Linqun Wang, 2020 | China | Unclear | Unclear | Unclear | Unclear | Low | Low | Low |
| Lin Wang, 2020 | China | Low | High | Unclear | Unclear | Low | Unclear | Unclear |
| Jianming Wang, 2021 | China | Low | High | Unclear | Unclear | Low | Unclear | Unclear |
| Yali Wang, 2020 | China | Unclear | Unclear | Unclear | Unclear | Low | Low | Unclear |
| Fangdan Wang, 2020 | China | Low | High | Unclear | Unclear | Low | Unclear | Unclear |
| WANG Jia-bo, 2020 | China | Low | Low | Low | Low | Low | Low | Low |
| Jingling zhou, 2021 | China | Low | Low | Unclear | Unclear | Unclear | Unclear | Unclear |
| Long Wen, 2020 | China | High | Low | Unclear | Unclear | Unclear | Unclear | Low |
| Yanshan Wei,2021 | China | High | High | High | Unclear | Unclear | Unclear | Low |
| Yue Wang, 2021 | China | Unclear | Unclear | Unclear | Unclear | Unclear | Unclear | Unclear |
| Mingzhong Xiao, 2020 | China | Low | Unclear | Unclear | Unclear | Low | High | Low |
| Hongbo Zhou, 2021 | China | Unclear | Unclear | Unclear | Unclear | Low | Low | Low |
| Xiangyong Yan, 2021 | China | High | Unclear | Unclear | Unclear | Low | Low | Low |
| Yan Xie, 2020 | China | Low | Unclear | Unclear | Unclear | High | Low | Low |
| Haiyan Wu, 2020 | China | Unclear | Unclear | Unclear | Unclear | Low | Low | Unclear |
| Xiaolong Xu, 2021 | China | Low | Unclear | Low | Low | Low | Low | Low |
| Wuzhong Xiong, 2020 | China | Low | Unclear | Unclear | Unclear | Low | Low | Low |
| Chao Yang, 2021 | China | Low | Low | Low | Unclear | Low | Low | Low |
| Zancuo Yang, 2021 | China | Low | Unclear | Unclear | Unclear | Low | Low | Low |
| Yanqun Yang, 2021 | China | Unclear | Unclear | Unclear | Unclear | Low | Low | Low |
| Bohua Yan, 2020 | China | Low | Unclear | Unclear | Unclear | Low | Low | Unclear |
| Dan Zhang, 2021 | China | Low | Unclear | High | High | Low | Low | Low |
| Ping Yu, 2020 | China | Low | Unclear | Unclear | Unclear | Low | Low | Unclear |
| Congcong Zeng, 2021 | China | Low | Low | Low | Unclear | Low | Unclear | Low |
| Xudong Yang， 2022 | China | Low | Unclear | Unclear | Unclear | Low | Unclear | Low |
| Yanyun Zheng, 2010 | China | Unclear | Unclear | Unclear | Unclear | Low | Low | Low |
| Jialiang Zhao, 2021 | China | Low | Unclear | Unclear | Unclear | Low | Low | Low |
| Feng Zhao, 2020 | China | High | Unclear | Unclear | Unclear | Low | Low | Low |
| Chen Zhao, 2021 | China | Low | High | High | High | Low | Low | Low |
| Xinyi Zhang, 2021 | China | Low | Low | Unclear | Low | Low | Low | Low |
| Ling Zhang, 2022 | China | Low | Unclear | Unclear | Unclear | Low | Low | Low |
| Yusheng Yan, 2021 | China | Low | Unclear | Unclear | Unclear | Low | Low | Low |
| Fen Hu, 2020 | China | Low | Low | Unclear | Unclear | High | Low | Unclear |
| Kaifeng Liu, 2020 | China | Low | Unclear | Unclear | Unclear | Low | Low | Unclear |
| Xiaolong Chen, 2020 | China | Low | Unclear | Unclear | Unclear | Low | Low | Unclear |
| Yadong Li, 2020 | China | High | Unclear | Unclear | Unclear | Low | Low | Low |
| Yongjiang Liu, 2021 | China | High | Unclear | Unclear | Unclear | Low | Low | Unclear |
| Youli Zhang, 2020 | China | High | Unclear | Unclear | Unclear | Low | Low | Unclear |
| Xiaolong Chen, 2020 | China | Low | Unclear | Unclear | Unclear | Low | Low | Unclear |
| Shiqi Sun, 2021 | China | Low | Low | Unclear | Unclear | Low | Low | Low |
| Zhijian Luo, 2021 | China | Low | Low | Low | Low | Low | Low | Low |

**Table S6. Quality assessment results for SRs (AMSTAR-2)**

| **Author, year** | **Term 1** | **Term 2** | **Term 3** | **Term 4** | **Term 5** | **Term 6** | **Term 7** | **Term 8** | **Term 9** | **Term 10** | **Term 11** | **Term 12** | **Term 13** | **Term 14** | **Term 15** | **Term 16** | **Overall** |
| --- | --- | --- | --- | --- | --- | --- | --- | --- | --- | --- | --- | --- | --- | --- | --- | --- | --- |
| Caiyun Hu, 2020 | Yes | No | No | Partial Yes | No | Yes | No | Yes | Yes | No | Yes | Yes | Yes | Yes | Yes | Yes | Critical Low |
| Caiyun Hu, 2022 | Yes | Yes | No | Partial Yes | No | No | No | Yes | Yes | No | Yes | Yes | Yes | Yes | Yes | Yes | Low |
| Zitong Feng, 2021 | Yes | No | No | Partial Yes | No | Yes | No | Yes | Yes | No | No meta-analysis conducted | No meta-analysis conducted | Yes | Yes | No meta-analysis conducted | Yes | Critical Low |
| Zheng Fan, 2021 | Yes | No | No | Partial Yes | Yes | Yes | No | Yes | Yes | No | Yes | Yes | Yes | Yes | No | No | Critical Low |
| Yi Guo, 2020 | Yes | No | No | Partial Yes | No | Yes | No | Yes | Partial Yes | No | No | No | Yes | No | No | Yes | Critical Low |
| Zhihuan Zhou, 2020 | No | No | No | Partial Yes | No | Yes | No | Yes | No | No | No meta-analysis conducted | No meta-analysis conducted | No | No | No meta-analysis conducted | Yes | Critical Low |
| Lin Ang, 2020 | Yes | Yes | Yes | Partial Yes | Yes | Yes | Yes | Partial Yes | Yes | No | Yes | No | Yes | No | No | Yes | Low |
| Xin Cai, 2020 | Yes | No | No | Partial Yes | Yes | Yes | No | Partial Yes | Yes | No | Yes | No | Yes | No | Yes | No | Critical Low |
| Ansul Kumar, 2022 | Yes | Partial Yes | Partial Yes | Yes | Yes | Yes | Yes | Yes | Yes | Yes | Yes | Yes | Yes | Yes | No | Yes | Low |
| Fei Jiang, 2021 | Yes | Partial Yes | Yes | Yes | Yes | Yes | Yes | Yes | Yes | Yes | Yes | Yes | Yes | Yes | Yes | Yes | High |
| Feng Li, 2021 | Yes | No | Yes | Partial Yes | Yes | Yes | Partial Yes | Yes | Yes | No | Yes | Yes | Yes | Yes | Yes | Yes | Low |
| Shibing Liang, 2021 | Yes | No | Yes | Partial Yes | No | Yes | Partial Yes | Partial Yes | Yes | No | Yes | Yes | Yes | Yes | Yes | Yes | Low |
| Jieqin Zhuang, 2021 | Yes | Yes | Yes | Partial Yes | Yes | Yes | Partial Yes | Partial Yes | Yes | No | Yes | No | Yes | Yes | Yes | Yes | Moderate |
| Aihua Liu, 2021 | Yes | No | No | Partial Yes | No | No | No | No | Yes | No | Yes | No | No | No | No | Yes | Critical Low |
| Lingling Liu, 2021 | Yes | No | Yes | Partial Yes | Yes | No | Partial Yes | Partial Yes | Yes | No | Yes | No | Yes | Yes | Yes | Yes | Low |
| Lizhao Yan, 2021 | Yes | No | No | Partial Yes | No | Yes | No | Yes | Yes | No | Yes | No | No | No | No | Yes | Critical Low |
| Xingjiang Xiong, 2020 | Yes | No | No | Yes | Yes | Yes | No | Yes | Yes | No | Yes | No | No | No | Yes | Yes | Critical Low |
| Huiyue Zhang, 2020 | Yes | No | No | No | Yes | Yes | No | Partial Yes | Yes | No | Yes | Yes | Yes | Yes | Yes | No | Critical Low |
| Meng Yang,2020 | Yes | No | No | Yes | No | No | No | Partial Yes | No | No | Yes | Yes | No | Yes | Yes | Yes | Critical Low |
| Guang Yang, 2021 | Yes | No | No | Partial Yes | Yes | Yes | No | Partial Yes | Yes | No | Yes | Yes | Yes | Yes | No | Yes | Critical Low |
| Mengjie Zeng，2020 | Yes | No | No | Yes | No | No | No | Partial Yes | Yes | No | Yes | Yes | Yes | Yes | Yes | Yes | Critical Low |
| Ruizhe Yu, 2022 | Yes | No | No | Partial Yes | No | No | No | Partial Yes | Yes | No | Yes | Yes | Yes | Yes | Yes | Yes | Critical Low |
| Bei Yin, 2021 | Yes | No | No | Yes | Yes | No | No | Yes | Yes | No | Yes | Yes | Yes | Yes | No | Yes | Critical Low |
| Ming Liu, 2020 | Yes | Yes | No | Partial Yes | Yes | Yes | No | Yes | Yes | No | Yes | Yes | Yes | Yes | Yes | Yes | Low |
| Ming Liu, 2021 | Yes | Yes | No | Yes | Yes | Yes | No | Partial Yes | Yes | No | Yes | No | Yes | Yes | No | Yes | Critical Low |
| JiaHui Ouyang, 2021 | Yes | Yes | No | Partial Yes | Yes | Yes | No | Yes | Yes | No | Yes | Yes | Yes | Yes | Yes | Yes | Low |
| Guodong Qi, 2020 | Yes | No | No | No | Yes | Yes | No | No | Yes | No | Yes | No | No | No | No | Yes | Critical Low |
| Fangfang Zhou, 2021 | Yes | No | No | Partial Yes | Yes | Yes | No | No | Yes | No | Yes | Yes | Yes | Yes | No | Yes | Critical Low |
| Shihua Shi1, 2021 | Yes | Yes | Yes | Yes | Yes | Yes | No | Yes | Yes | No | Yes | Yes | Yes | Yes | No | Yes | Critical Low |
| Shihua Shi2, 2021 | Yes | Yes | Yes | Yes | Yes | Yes | No | Yes | Yes | No | Yes | Yes | Yes | Yes | Yes | Yes | Low |
| Chengqian Shi, 2022 | Yes | Yes | No | Partial Yes | No | Yes | No | Yes | Yes | No | Yes | Yes | No | No | Yes | Yes | Critical Low |
| Wentai Pang, 2020 | Yes | Yes | No | Yes | Yes | Yes | No | Yes | Yes | No | Yes | Yes | Yes | No | Yes | Yes | Low |
| Qinxin Zheng, 2021 | Yes | No | No | No | No | No | Yes | No | Yes | No | Yes | Yes | Yes | Yes | Yes | No | Critical Low |
| Wenbin Zhang, 2020 | Yes | No | No | Partial Yes | No | Yes | Yes | No | Yes | No | Yes | No | No | No | No | No | Critical Low |
| Lepeng Zhou, 2020 | Yes | Yes | No | Yes | Yes | Yes | Yes | No | Yes | No | Yes | Yes | Yes | Yes | Yes | Yes | Moderate |
| Xiaozheng Wu, 2021 | Yes | Yes | No | Yes | Yes | Yes | No | Yes | Yes | No | Yes | Yes | Yes | Yes | Yes | Yes | Low |
| Yangzihan Wang, 2022 | No | No | No | Partial Yes | Yes | Yes | Yes | Partial Yes | Yes | No | Yes | Yes | Yes | No | No | Yes | Critical Low |
| Qi Wang, 2021 | Yes | Yes | No | Yes | Yes | Yes | Yes | Yes | Yes | No | Yes | No | No | No | Yes | Yes | Low |
| Heping Wang,2021 | Yes | Yes | No | Yes | Yes | Yes | No | Yes | Yes | No | Yes | Yes | Yes | Yes | Yes | Yes | Low |
| Dengchao Wang, 2021 | Yes | No | No | Partial Yes | No | Yes | No | Partial Yes | Yes | No | Yes | No | No | Yes | No | Yes | Critical Low |
| Chunyang Sun, 2020 | Yes | No | No | Partial Yes | Yes | No | No | Yes | Yes | No | Yes | No | No | Yes | No | Yes | Critical Low |
| Shuxia Wang, 2020 | Yes | No | No | Partial Yes | No | Yes | No | Yes | Yes | No | Yes | Yes | Yes | Yes | Yes | No | Critical Low |
| Qianfei Wang, 2021 | Yes | No | No | Partial Yes | No | Yes | No | Yes | Yes | No | Yes | No | No | No | No | Yes | Critical Low |
| Xufei Luo, 2021 | Yes | Yes | No | Yes | Yes | Yes | No | Yes | Yes | No | Yes | No | No | Yes | Yes | Yes | Critical Low |
| Yanli Tang, 2021 | Yes | No | No | Partial Yes | Yes | Yes | No | Yes | Yes | No | Yes | No | No | Yes | Yes | Yes | Critical Low |
| Heasun Chun,2021 | Yes | No | Yes | Yes | Yes | Yes | Yes | Yes | Yes | Yes | Yes | Yes | Yes | Yes | Yes | Yes | Low |
| Xuqin Du1,2021 | Yes | Yes | Yes | Yes | Yes | Yes | Yes | Yes | Yes | Yes | Yes | Yes | Yes | Yes | Yes | Yes | High |
| Xuqin Du2,2021 | Yes | Yes | Yes | Yes | Yes | Yes | Yes | Yes | Yes | Yes | Yes | Yes | Yes | Yes | Yes | Yes | High |
| Arthur Yin Fan, 2020 | Yes | Yes | Yes | Yes | Yes | Yes | Yes | Yes | Yes | Yes | Yes | Yes | Yes | Yes | Yes | Yes | High |
| Yuqin Wu, 2020 | Yes | No | No | Partial Yes | No | No | No | Yes | Yes | No | Yes | Yes | Yes | Yes | Yes | Yes | Critical Low |

*Term 1. Covering "PICO"; Term 2. Following review protocol; Term 3. Study design selection; Term 4. Literature search strategy; Term 5. Duplicated coding for study selection; Term 6. Duplicated coding for data extraction; Term 7. Justification of excluded papers; Term 8. Description of included studies; Term 9. Assessment of risk of bias; Term 10. Reporting study funding source; Term 11. Using appropriate statistical combination method; Term 12. ROB impact on meta-analysis; Term 13. Discussing of ROB impact; Term 14. Addressing heterogeneity; Term 15. Consideration of publication bias; Term 16. Reporting conflict of interest

**Table S7. Traditional Chinese medicine prescriptions (including main components)**

| **Study** | **Traditional Chinese medicine prescriptions** | **Main components** |
| --- | --- | --- |
| Yuqin Chen,2021 | Bufei Huoxue Capsules, BFHX | Preparation and Usage: obtained in the form of hard capsules (specifications: 0.35 g per capsule), four capsules at a time, three times a day. (China Pharmacopeia 2020: https://db.ouryao.com/yd2020/view.php?id=f0bd8165f8)  Main components: Psoraleae fructus (Fabaceae; Psoralea corylifolia Linn) (40%), Astragali radix (Fabaceae; Astragalus mongholicus Bunge) (40%), and Paeoniae radix rubra (Paeoniaceae; Paeonia lactiflora Pall) (20%). |
| Qing He, 2021 | Buzhong Yiqi Decoction, BZYQ | Preparation and Usage: decocted in water, one dose a day, twice in the morning and evening on an empty stomach.  Main components: 10 g Astragali radix (Fabaceae; Astragalus mongholicus Bunge), 3 g Radix Ginseng (Araliaceae; Panax ginseng C.A.Mey), 3 g Radix Angelicae Gigantis (Asclepiadaceae; Radix Angelicae Gigantis), 5 g Radix Glycyrrhizae (Fabaceae; Glycyrrhiza uralensis Fisch), 3 g Rhizoma Atractylodis (Asteraceae; Atractylodes lancea (Thunb.) DC), 3 g Rhizoma Cimicifugae (Ranunculaceae; Actaea cimicifuga L), 3g Pericarpium Aurantii Nobilis (Rutaceae; Citrus × aurantium L), and 3 g Radix Bupleuri (Apiaceae; Bupleurum chinense DC). |
| Wei Jin, 2020 | Fufang Yinchai granules + Qingqiao Jiedu granule, FFYC + QQJD | Preparation and Usage: obtained in the form of hard granules, 15 g at a time, four times a day.  Main component (FFYC): Lonice Raejaponicae Caulis (Caprifoliaceae; Lonicera japonica Thunb), Radix Bupleuri (Apiaceae; Bupleurum chinense DC), Arundo donax (Poaceae; Arundo donax L), Eriobotryae Folium (Rosaceae; Eriobotrya japonica（Thunb.）Lindl), Menthae Haplocalycis Herba (Lamiaceae; Mentha haplocalyx Briq), Pogostemonis Herba (Lamiaceae; Pogostemon cablin（Blanco）Benth), Schizonepetae Herba (Lamiaceae; Schizonepeta tenuifolia Briq)  Main component (QQJD): Lonice Raejaponicae Caulis (Caprifoliaceae; Lonicera japonica Thunb), Forsythiae Fructus (Oleaceae; Forsythia suspensa （Thunb.） Vahl), Puerariae Thomsonii Radix (Fabaceae; Pueraria thomsonii Benth), Angelicae Dahuricae Radix (Apiaceae; Angelica dahurica（Fisch.ex Hoffm.）Benth.et Hook.f.), Artemisiae Annuae Herba (Asteraceae; Artemisia annua L.), Radix Bupleuri (Apiaceae; Bupleurum chinense DC), Paridis Rhizoma (Liliaceae; Paris polyphylla Smith var.yunnanensis (Franch.)Hand.-Mazz), Isatidis Radix (Cruciferae; Isatis indigotica Fort.), Iridis Tectori Rhizoma (Iridaceae; Iris tectorum Maxim.), Taraxaci Herba (Asteraceae; Taraxacum mongolicum Hand. -Mazz.), Isatidis Folium (Brassicaceae; Isatis indigotica Fort.), Pogostemonis Herba (Lamiaceae; Pogostemon cablin（Blanco）Benth), Perillae Folium (Lamiaceae; Perilla frutescens（L.）Britt.), Peppermint Oil (Lamiaceae; Mentha haplocalyx Briq). |
| Xiaolong Chen, 2020 | Feilike capsule, FLK | Preparation and Usage: obtained in the form of hard capsules, three capsules at a time, three times a day.  Main components: Root of Phoenix Tree (Malvaceae; Firmiana plantanifolia（L.f.）Marsili), Gentianae Rhodanthae Herba (Gentianaceae; Gentiana rhodantha Franch.), Threevein Aster (Asteraceae; Aster ageratoides Turcz.), Peucedani Radix (Apiaceae; Peucedanum praeruptorum Dunn), stemonae radix (Stemonaceae; Stemona sessilifolia（Miq.）Miq.), Scutellariae Radix (Lamiaceae; Scutellaria baicalensis Georgi). |
| Suofang Shi, 2020 | Futu Shengjin Rehabilitation Formul, FTSJ | Preparation and Usage: obtained in the form of hard granules, 20 g at a time, two times a day.  Main components: Codonopsis Radix (Campanulaceae; Codonopsis pilosula (Franch.)Nannf.), Astragali radix (Fabaceae; Astragalus membranaceus（Fisch.） Bge.var.mongholicus（Bge.）Hsiao), Atractylodis Macrocephalae Rhizoma (Asteraceae; Atractylodes macrocephala Koidz.), Poria (Polyporaceae; Poria cocos（Schw.）Wolf), Pinelliae Rhizoma Praeparatum (Araceae; Pinellia ternata (Thunb.) Makino), Pericarpium Aurantii Nobilis (Rutaceae; Citrus × aurantium L), Juglandis Semen (Juglandaceae; Juglans regia L.), Dioscoreae Rhizoma (Dioscoreaceae; Dioscorea opposita Thunb.), Inulae Flos (Asteraceae; Inula japonica Thunb.), Cyperi Rhizoma (Cyperaceae; Cyperus rotundus L.), Rubiae Radix et Rhizoma (Rubiaceae; Rubia cordifolia L.), Albiziae Cortex (Fabaceae; Albizia julibrissin Durazz.), Fructus Setariae Germinatus (Poaceae; Setaria italica （L. ） Beauv.), Radix Glycyrrhizae (Fabaceae; Glycyrrhiza uralensis Fisch). |
| Kequn Chai, 2021 | Feiyan 1+2, FY 1+2 | Preparation and Usage: decocted in water, one dose a day, twice in the morning and evening.  Main components (FY 1): Tetrastigma hemsleyanum (Vitaceae; Tetrastigma hemsleyanum Diels & Gilg) 20g，Schizonepetae Herba (Lamiaceae; Schizonepeta tenuifolia Briq)12g, Radix Saposhnikoviae (Apiaceae; Saposhnikovia divaricata（Turcz.）Schischk.) 9g, Forsythiae Fructus (Oleaceae; Forsythia suspensa （Thunb.） Vahl)18g, Lonicerae Japonicae Flos (Caprifoliaceae; Lonicera japonica Thunb.)9g, Peucedani Radix (Apiaceae; Peucedanum praeruptorum Dunn) 9g, Pogostemonis Herba (Lamiaceae; Pogostemon cablin（Blanco）Benth.) 9g, Rhizoma Atractylodis (Asteraceae; Atractylodes lancea (Thunb.) DC)12g, Phragmitis Rhizoma (Poaceae; Phragmites communis Trin.) 30g, Radix Platycodonis (Campanulaceae; Platycodon grandiflorum （Jacq.）A.DC.) 6g, Radix Glycyrrhizae (Fabaceae; Glycyrrhiza uralensis Fisch)5g.  Main components (FY 2): Radix et Rhizoma Rhei (Polygonaceae; Rheum palmatum L.) 9g, Gypsum Fibrosum (CaSO4·2H2O) 30g, Natrii Sulfas (Na2SO4•10H2O) 9g, Cornu Bubali (Bubalus bubalis Linnaeus) 30g, Rhizoma Atractylodis (Asteraceae; Atractylodes lancea (Thunb.) DC)15g, Scrophulariae Radix (Scrophulariaceae; Scrophularia ningpoensis Hemsl.) 15g, Curcumae Radix (Zingiberaceae; Curcuma wenyujin Y. H. Chen et C. Ling) 15g, Bulbus Fritillariae Thunbergii (Liliaceae; Fritillaria thunbergii Miq.) 15g, Descurainiae Semen Lepidii Semen (Brassicaceae; Descurainia sophia（L.）Webb. ex Prantl.) 20g, Moutan Cortex (Ranunculaceae; Paeonia suffruticosa Andr.) 15g, Paeoniae Radix Rubra (Ranunculaceae; Paeonia lactiflora Pall.) 15g, Ginseng Radix et Rhizoma (Araliaceae; Panax ginseng C. A. Mey.) 6g, Radix Glycyrrhizae (Fabaceae; Glycyrrhiza uralensis Fisch)9g. |
| Xiangying Ai, 2020 | Feiyan Yihao Chinese medicine granules, FYYH | Preparation and Usage: decocted in water, one dose a day.  Main components: Artemisiae Annuae Herba (Asteraceae; Artemisia annua L.) 10 g, Astragali radix (Fabaceae; Astragalus membranaceus（Fisch.） Bge.var.mongholicus（Bge.）Hsiao) 45 g, Cremastrae Pseudobulbus Pleiones Pseudobulbus (Cremastra appendiculata（D.Don）Makino; Cremastra appendiculata（D.Don）Makino) 20 g, Forsythiae Fructus (Oleaceae; Forsythia suspensa （Thunb.） Vahl) 30 g, Scutellariae Radix (Lamiaceae; Scutellaria baicalensis Georgi) 10 g, Lonicerae Japonicae Flos (Caprifoliaceae; Lonicera japonica Thunb.) 15 g, Isatidis Folium (Brassicaceae; Isatis indigotica Fort.)10 g, Radix Bupleuri (Apiaceae; Bupleurum chinense DC) 5 g, Cicadae Periostracum (Cicadidae; Cryptotympana pustulata Fabricius) 10 g, Peucedani Radix (Apiaceae; Peucedanum praeruptorum Dunn) 5 g, Fritillariae Cirrhosae Bulbus (Liliaceae; Fritillaria cirrhosa D.Don)10 g, fritillariae thunbergii Bulbus (Liliaceae; Fritillaria thunbergii Miq.) 10 g, Mume Fructus (Rosaceae; Prunus mume（Sieb.）Sieb.etZucc.) 30 g, Scrophulariae Radix (Scrophulariaceae; Scrophularia ningpoensis Hemsl.) 10 g, Poria (Polyporaceae; Poria cocos（Schw.）Wolf) 30 g，Pseudostellariae Radix (Caryophyllaceae; Pseudostellaria heterophylla（Miq.）Pax ex Pax et Hoffm.) 15 g |
| Dan Zhang, 2021 | Fuzheng Gubiao Fanggan particles, FZGBFG | Preparation and Usage: decocted in water, one dose a day, twice in the morning and evening.  Main components: Astragali radix (Fabaceae; Astragalus membranaceus（Fisch.） Bge.var.mongholicus（Bge.）Hsiao) 12g, Atractylodis Macrocephalae Rhizoma (Asteraceae; Atractylodes macrocephala Koidz.) 9g, Radix Saposhnikoviae (Apiaceae; Saposhnikovia divaricata （Turcz.）Schischk.) 6g, Poria (Polyporaceae; Poria cocos(Schw.)Wolf ) 12g，Pericarpium Aurantii Nobilis (Rutaceae; Citrus × aurantium L)6g, Forsythiae Fructus (Oleaceae; Forsythia suspensa （Thunb.） Vahl)9g, Lonicerae Japonicae Flos (Caprifoliaceae; Lonicera japonica Thunb.) 10g, Perillae Folium (Lamiaceae; Perilla frutescens（L.）Britt.) 6g, Radix Glycyrrhizae (Fabaceae; Glycyrrhiza uralensis Fisch)3g, Coicis Semen (Poaceae; Coix lacryma-jobi L.var.ma-yuen(Roman.) Stapf) 15g, Pogostemonis Herba (Lamiaceae; Pogostemon cablin（Blanco）Benth.)10g, Radix Platycodonis (Campanulaceae; Platycodon grandiflorum （Jacq.）A.DC.) 9g. |
| Linqun Wang, 2020 | Gegen Qinlian Pills, GGQL | Preparation and Usage: obtained in the form of pills, 3g at a time, three times a day. (China Pharmacopeia 2020: https://db.ouryao.com/yd2020/view.php?id=f6ddd13f27)  Main components: Puerariae Lobatae Radix (Fabaceae; Pueraria lobata（Willd.）Ohwi), Scutellariae Radix (Lamiaceae; Scutellaria baicalensis Georgi), Coptidis Rhizoma (Ranunculaceae; Coptis chinensis Franch.), Radix Glycyrrhizae (Fabaceae; Glycyrrhiza uralensis Fisch). |
| Hongbo Zhou, 2021 | Hamao oil pieces | Preparation and Usage: decocted in water, 0.2g at a time, two times a day.  Main components: Oviductus ranae. |
| Jia Liu, 2021  Chen Zhao, 2021  Yongjiang Liu, 2021 | Huashibaidu Formula, HSBD | Preparation and Usage: decocted in water, one-two dose a day, twice-four times a day.  Main components: Ephedrae Herba (Ephedraceae; Ephedra sinica Stapf) 6g, Armeniacae Semen Amarum (Rosaceae; Prunus armeniaca L.var.ansu Maxim.) 9g, Gypsum Fibrosum (CaSO4·2H2O) 15g, Radix Glycyrrhizae (Fabaceae; Glycyrrhiza uralensis Fisch)3g, Pogostemonis Herba (Lamiaceae; Pogostemon cablin（Blanco）Benth.) 10g, Magnoliae Officinalis Cortex (Magnoliaceae; Magnolia officinalis Rehd.et Wils.)10g, Rhizoma Atractylodis (Asteraceae; Atractylodes lancea (Thunb.) DC) 15g, Tsaoko Fructus (Zingiberaceae; Amomum tsao-ko Crevost et Lemaire) 10g, Pinelliae Rhizoma Praeparatum (Araceae; Pinellia ternata (Thunb.) Makino) 9g, Poria (Polyporaceae; Poria cocos（Schw.）Wolf) 15g, Radix et Rhizoma Rhei (Polygonaceae; Rheum palmatum L.), Astragali radix (Fabaceae; Astragalus mongholicus Bunge) 10g, Descurainiae Semen Lepidii Semen (Brassicaceae; Descurainia sophia（L.）Webb. ex Prantl.) 10g, Paeoniae Radix Rubra (Ranunculaceae; Paeonia lactiflora Pall.) 10g. |
| Mingzhong Xiao, 2020 | Huoxiang Zhengqi dropping pills+ Lianhua Qingwen granules/capsules, HXZQ+ LHQW | reparation and Usage (HXZQ): obtained in the form of pills, 2.6-5.2g at a time, two times a day. (China Pharmacopeia 2020: https://db.ouryao.com/yd2020/view.php?id=f06dbe74fb)  Preparation and Usage (LHQW): obtained in the form of hard granules/capsules, 6g/4 capsules at a time, three times a day. (China Pharmacopeia 2020: https://db.ouryao.com/yd2020/view.php?id=fe3dfefc6d; https://db.ouryao.com/yd2020/view.php?id=fe7d49e1ac)  Main component (HXZQ): Pogostemonis Herba (Lamiaceae; Pogostemon cablin（Blanco）Benth), Rhizoma Atractylodis (Asteraceae; Atractylodes lancea (Thunb.) DC.), Magnoliae Officinalis Cortex (Magnoliaceae; Magnolia officinalis Rehd.et Wils.), Angelicae Dahuricae Radix (Apiaceae; Angelica dahurica（Fisch.ex Hoffm.）Benth.et Hook.f.), Arecae Semen (Arecaceae; Areca catechu L.), Pinelliae Rhizoma (Araceae; Pinellia ternata（Thunb.） Breit.), Radix Glycyrrhizae (Fabaceae; Glycyrrhiza uralensis Fisch), Perillae Folium (Lamiaceae; Perilla frutescens（L.）Britt.).  Main component (LHQW): Forsythiae Fructus (Oleaceae; Forsythia suspensa （Thunb.） Vahl), Ephedrae Herba (Ephedraceae; Ephedra sinica Stapf), Lonicerae Japonicae Flos (Caprifoliaceae; Lonicera japonica Thunb.), Isatidis Radix (Cruciferae; Isatis indigotica Fort.), Menthae Haplocalycis Herba (Lamiaceae; Mentha haplocalyx Briq), Dryopteridis Crassirhizomatis Rhizoma (Dryopteridaceae; Dryopteris crassirhizoma Nakai), Rhodiolae Crenulatae Radix et Rhizoma (Crassulaceae; Rhodiola crenulata （Hook. f. et Thoms. ）H. Ohba), Gypsum Fibrosum (CaSO4·2H2O), Pogostemonis Herba (Lamiaceae; Pogostemon cablin（Blanco）Benth), Radix et Rhizoma Rhei (Polygonaceae; Rheum palmatum L.), Houttuyniae Herba (Saururaceae; Houttuynia cordata Thunb.), and Radix Glycyrrhizae (Fabaceae; Glycyrrhiza uralensis Fisch). |
| Yanqun Yang, 2021 | Modified Jiegeng Xingren Decoction, JGXR | Preparation and Usage: decocted in water, one dose a day.  Main components: Radix Platycodonis (Campanulaceae; Platycodon grandiflorum （Jacq.）A.DC.) 15 g, Armeniacae Semen Amarum (Rosaceae; Prunus armeniaca L.var.ansu Maxim.) 12 g, Forsythiae Fructus (Oleaceae; Forsythia suspensa （Thunb.） Vahl) 10 g, Prunellae Spica (Lamiaceae; Prunella vulgaris L.) 10g, Bulbus Fritillariae Thunbergii (Liliaceae; Fritillaria thunbergii Miq.) 10 g, Sargent gloryvine (Arecaceae; Daemonorops margaritae(Hance)Becc.) 15g, Moutan Cortex (Ranunculaceae; Paeonia suffruticosa Andr.) 15 g, Phragmitis Rhizoma (Poaceae; Phragmites communis Trin.) 20 g, Radix Glycyrrhizae (Fabaceae; Glycyrrhiza uralensis Fisch) 6 g. |
| Bohua Yan, 2020 | Jinhao Jiere granules + Huoxiangzhengqi oral liquids, JHJR + HXZQ | Preparation and Usage (JHJR): obtained in the form of hard granules, 8g at a time, three times a day.  Preparation and Usage (HXZQ): obtained in the form of mixed liquid, 5~10ml a time at a time, two times a day. (China Pharmacopeia 2020: https://db.ouryao.com/yd2020/view.php?id=ff6d6d452b)  Main component (JHJR): Artemisiae Annuae Herba (Asteraceae; Artemisia annua L.), Lonicerae Japonicae Flos (Caprifoliaceae; Lonicera japonica Thunb.), Schizonepetae Herba (Lamiaceae; Schizonepeta tenuifolia Briq), Scutellariae Radix (Lamiaceae; Scutellaria baicalensis Georgi), Radix Saposhnikoviae (Apiaceae; Saposhnikovia divaricata（Turcz.）Schischk.), Radix Platycodonis (Campanulaceae; Platycodon grandiflorum （Jacq.）A.DC.).  Main component (HXZQ): Rhizoma Atractylodis (Asteraceae; Atractylodes lancea (Thunb.) DC), Pericarpium Aurantii Nobilis (Rutaceae; Citrus × aurantium L), Magnoliae Officinalis Cortex (Magnoliaceae; Magnolia officinalis Rehd.et Wils.), Angelicae Dahuricae Radix (Apiaceae; Angelica dahurica（Fisch.ex Hoffm.）Benth.et Hook.f.), Poria (Polyporaceae; Poria cocos（Schw.）Wolf), Arecae Pericarpium (Palmaceous; Areca catechu L.), Pinelliae Rhizoma (Araceae; Pinellia ternata（Thunb.） Breit.) 9g, Radix Glycyrrhizae (Fabaceae; Glycyrrhiza uralensis Fisch), Patchouli Oil (Lamiaceae; Pogostemon cablin （Blanco）Benth), Perilla leaf oil (Lamiaceae; Perilla frutescens(L.) Britt). |
| Xuedong An, 2021  Can Duan, 2020 | Jinhua Qinggan granules, JHQG | Preparation and Usage: obtained in the form of hard granules, 5g at a time, three times a day.  Main components: Lonicerae Japonicae Flos (Caprifoliaceae; Lonicera japonica Thunb.), Gypsum Fibrosum (CaSO4·2H2O), Ephedrae Herba (Ephedraceae; Ephedra sinica Stapf), Armeniacae Semen Amarum (Rosaceae; Prunus armeniaca L.var.ansu Maxim.), Scutellariae Radix (Lamiaceae; Scutellaria baicalensis Georgi), Forsythiae Fructus (Oleaceae; Forsythia suspensa （Thunb.） Vahl), Bulbus Fritillariae Thunbergii (Liliaceae; Fritillaria thunbergii Miq.), Anemarrhenae Rhizoma (Liliaceae; Anemarrhena asphodeloides Bge.), Arctii Fructus (Asteraceae; Arctium lappa L.), Artemisiae Annuae Herba (Asteraceae; Artemisia annua L.), Menthae Haplocalycis Herba (Lamiaceae; Mentha haplocalyx Briq), and Radix Glycyrrhizae (Fabaceae; Glycyrrhiza uralensis Fisch). |
| Fen Hu, 2020  Youli Zhang, 2020 | Jinyinhua Oral Liquid, JYH | Preparation and Usage: obtained in the form of mixed liquid, 20~60ml at a time, two times a day.  Main components: Lonicerae Japonicae Flos (Caprifoliaceae; Lonicera japonica Thunb.) |
| Jialiang Zhao, 2021 | Kangbingdu 1, KBD 1 | Preparation and Usage: decocted in water, one dose a day, three times a day.  Main components: Rhizoma Atractylodis (Asteraceae; Atractylodes lancea (Thunb.) DC) 15 g, Pericarpium Aurantii Nobilis (Rutaceae; Citrus × aurantium L) 10 g, Magnoliae Officinalis Cortex (Magnoliaceae; Magnolia officinalis Rehd.et Wils.) 10 g, Pogostemonis Herba (Lamiaceae; Pogostemon cablin（Blanco）Benth.) 10 g, Tsaoko Fructus (Zingiberaceae; Amomum tsao-ko Crevost et Lemaire) 15 g, Ephedrae Herba (Ephedraceae; Ephedra sinica Stapf) 5 g, Notopterygii Rhizoma et Radix (Apiaceae; Notopterygium incisum Ting ex H. T. Chang) 10 g, Rhizoma Zingiberis Recens (Zingiberaceae; Rhizoma Zingiberis Recens) 10 g, Armeniacae Semen Amarum (Rosaceae; Prunus armeniaca L.var.ansu Maxim.) 10 g, Amomi Fructus Rotundus (Zingiberaceae; Amomum kravanh Pierre ex Gagnep.) 10 g, Coicis Semen (Poaceae; Coix lacryma-jobi L.var.ma-yuen(Roman.) Stapf) 15 g, Astragali radix (Fabaceae; Astragalus membranaceus（Fisch.） Bge.var.mongholicus（Bge.）Hsiao) 20 g, Notoginseng Radix Et Rhizoma (Araliaceae; Panax notoginseng （ Burk.） F. H. Chen) 15 g, Pinelliae Rhizoma Praeparatum (Araceae; Pinellia ternata (Thunb.) Makino) 15 g, Radix Ginseng (Araliaceae; Panax ginseng C.A.Mey) 20 g. |
| WANG Jia-bo, 2020 | Keguan-1 | Preparation and Usage: obtained in the form of powder versions, 19.4 g twice daily.  Main components: Lonicerae Japonicae Flos (Caprifoliaceae; Lonicera japonica Thunb.) 30 g, Forsythiae Fructus (Oleaceae; Forsythia suspensa （Thunb.） Vahl) 30 g, Mori Cortex (Moraceae; Morus alba L.) 15 g, Chrysanthemi Flos (Asteraceae; Chrysanthemum morifolium Ramat.) 10 g, Coicis Semen (Poaceae; Coix lacryma-jobi L.var.ma-yuen(Roman.) Stapf) 30 g, Bulbus Fritillariae Thunbergii (Liliaceae; Fritillaria thunbergii Miq.) 15 g, and Armeniacae Semen Amarum (Rosaceae; Prunus armeniaca L.var.ansu Maxim.) 9g. |
| Fataneh Hashem-Dabaghian, 2021 | Lavender syrup decoction | Preparation and Usage: obtained in the form of mixed liquid, 9ml at a time, two times a day.  Main components: lavender (Lamiaceae; Lavandula angustifolia L.). |
| Ling Zhang, 2022  Huimin Sun, 2020 | Lianhua Qingke tablets, LHQK | Preparation and Usage: obtained in the form of tablets, four tablets at a time, three times a day.  Main components: Ephedrae Herba (Ephedraceae; Ephedra sinica Stapf), Mori Cortex (Moraceae; Morus alba L.), Gypsum Fibrosum (CaSO4·2H2O), Scutellariae Radix (Lamiaceae; Scutellaria baicalensis Georgi), Armeniacae Semen Amarum (Rosaceae; Prunus armeniaca L.var.ansu Maxim.), Forsythiae Fructus (Oleaceae; Forsythia suspensa （Thunb.） Vahl), Pinelliae Rhizoma (Araceae; Pinellia ternata（Thunb.） Breit.), Bulbus Fritillariae Thunbergii (Liliaceae; Fritillaria thunbergii Miq.), Peucedani Radix (Apiaceae; Peucedanum praeruptorum Dunn), Arctii Fructus (Asteraceae; Arctium lappa L.), Lonicerae Japonicae Flos (Caprifoliaceae; Lonicera japonica Thunb.), Radix et Rhizoma Rhei (Polygonaceae; Rheum palmatum L.), and Pericarpium Aurantii Nobilis (Rutaceae; Citrus × aurantium L). |
| Chaowu Chen,2021  Ke Hu, 2021  Ping Yu, 2020  Caiyun Hu, 2020  Caiyun Hu2022  Zheng Fan, 2021  Zhuang, J., 2021  Ming Liu, 2021  Guodong Qi, 2020  Yanli Tang, 2021  Chengqian Shi, 2022  Deng-chao Wang, 2021  Shuxia Wang, 2020  Huiyue Zhang, 2020  Meng Yang,2020  Mengjie Zeng，2020  Wenbin Zhang, 2020 | Lianhua Qingwen granules/capsules, LHQW | Preparation and Usage (LHQW): obtained in the form of hard granules/capsules, 6g/4 capsules at a time, three times a day. (China Pharmacopeia 2020: https://db.ouryao.com/yd2020/view.php?id=fe3dfefc6d; https://db.ouryao.com/yd2020/view.php?id=fe7d49e1ac)  Main components: Forsythiae Fructus (Oleaceae; Forsythia suspensa （Thunb.） Vahl), Ephedrae Herba (Ephedraceae; Ephedra sinica Stapf), Lonicerae Japonicae Flos (Caprifoliaceae; Lonicera japonica Thunb.), Isatidis Radix (Cruciferae; Isatis indigotica Fort.), Menthae Haplocalycis Herba (Lamiaceae; Mentha haplocalyx Briq), Dryopteridis Crassirhizomatis Rhizoma (Dryopteridaceae; Dryopteris crassirhizoma Nakai), Rhodiolae Crenulatae Radix et Rhizoma (Crassulaceae; Rhodiola crenulata （Hook. f. et Thoms. ）H. Ohba), Gypsum Fibrosum (CaSO4·2H2O), Pogostemonis Herba (Lamiaceae; Pogostemon cablin（Blanco）Benth), Radix et Rhizoma Rhei (Polygonaceae; Rheum palmatum L.), Houttuyniae Herba (Saururaceae; Houttuynia cordata Thunb.), and Radix Glycyrrhizae (Fabaceae; Glycyrrhiza uralensis Fisch). |
| Wu Liu, 2021 | Lianhua Qingwen granules/capsules + Feiyan 2, LHQW + FY2 | Preparation and Usage (LHQW): obtained in the form of hard granules/capsules, 6g/4 capsules at a time, three times a day. (China Pharmacopeia 2020: https://db.ouryao.com/yd2020/view.php?id=fe3dfefc6d; https://db.ouryao.com/yd2020/view.php?id=fe7d49e1ac)  Preparation and Usage (FY2): decocted in water, one dose a day, twice times a day.  Main components (FY 2): Armeniacae Semen Amarum (Rosaceae; Prunus armeniaca L.var.ansu Maxim.), Ephedrae Herba (Ephedraceae; Ephedra sinica Stapf), Ginkgo Semen (Ginkgoaceae; Ginkgo biloba L.), Pheretima (Megascolecidae; Pheretima aspergillum(E.Perrier)), Descurainiae Semen Lepidii Semen (Brassicaceae; Descurainia sophia（L.）Webb. ex Prantl.), Schisandrae Chinensis Fructus (Schisandraceae; Schisandra chinensis（Turcz.）Baill.), Pinelliae Rhizoma (Araceae; Pinellia ternata（Thunb.） Breit.), Radix Glycyrrhizae (Fabaceae; Glycyrrhiza uralensis Fisch), Perillae Fructus (Lamiaceae; Perilla frutescens(L.) Britt.), Mori Cortex (Moraceae; Morus alba L.), Farfarae Flos (Asteraceae; Tussilago farfara L.). |
| Shiqi Sun, 2021 | Liushen Pill, LS | Preparation and Usage: obtained in the form of pills, 10 capsules at a time, three times a day.  Main components: Bezoar (the gall-stone of Bos taurus domesticus Gmelin), Musk (the excretion of Moschus), cinobufagin venom toad (the excretion of Venenum Bufonis), pearl (the shell of Pernulo), realgar, and borneol. |
| Congcong Zeng, 2021 | Maxingshigan-Weijing Decoction, MXSGWJ | Preparation and Usage: decocted in water, one dose a day.  Main components: Ephedrae Herba (Ephedraceae; Ephedra sinica Stapf), 10 g of Armeniacae Semen Amarum (Rosaceae; Prunus armeniaca L.var.ansu Maxim.), 45 g of Gypsum Fibrosum (CaSO4·2H2O), 30 g of Phragmitis Rhizoma (Poaceae; Phragmites communis Trin.), 20 g of Persicae semen (Rosaceae; Prunus persica（L.）Batsch), 20 g of winter melon kernel (Dongguaren), 30 g of Trichosanthis Fructus (Cucurbitaceae; Trichosanthes kirilowii Maxim.), 12 g of Pericarpium Aurantii Nobilis (Rutaceae; Citrus × aurantium L), 12 g of Pinelliae Rhizoma Praeparatum cum Zingibere et Alumine (Araceae; Pinellia ternata (Thunb.) Makino), 12 g of Bambusae Caulis In Taenias (Poaceae; Bambusa tuldoides Munro), 30 g of Descurainiae Semen Lepidii Semen (Brassicaceae; Descurainia sophia（L.）Webb. ex Prantl.), 15 g of Acori Tatarinowii Rhizoma (Araceae; Acorus tatarinowii Schott), 10 g of curcuma zedoary (ezhu) and 5 g of Radix Glycyrrhizae (Fabaceae; Glycyrrhiza uralensis Fisch). |
| Min Qiu, 2020 | Maxing Xuanfei Jiedu Decoction, MXXFJD | Preparation and Usage: decocted in water, one dose a day, three time a day.  Main components: Ephedrae Herba (Ephedraceae; Ephedra sinica Stapf) 9 g, Armeniacae Semen Amarum (Rosaceae; Prunus armeniaca L.var.ansu Maxim.) 12 g, Gypsum Fibrosum (CaSO4·2H2O) 15~30 g, Bulbus Fritillariae Thunbergii (Liliaceae; Fritillaria thunbergii Miq.) 12 g, Cicadae Periostracum (Cicadidae; Cryptotympana pustulata Fabricius)10 g, Bombyx Batryticatus (Bombycidae; Bombyx mori Linnaeus) 15 g, Curcumae Longae Rhizoma (Zingiberaceae; Curcuma Longa L.) 12 g, Radix Platycodonis (Campanulaceae; Platycodon grandiflorum （Jacq.）A.DC.) 12 g, Aurantii Fructus (Rutaceae; Citrus aurantium L.)12 g, Tsaoko Fructus (Zingiberaceae; Amomum tsao-ko Crevost et Lemaire)9 g, Amomi Fructus Rotundus (Zingiberaceae; Amomum kravanh Pierre ex Gagnep.) 12 g. |
| Yue Wang, 2021  Yadong Li, 2020  Qi Wang, 2021 | Qingfei Paidu Decoction, QFPD | Preparation and Usage: decocted in water, one dose a day, two times a day.  Main components: Ephedrae Herba (Ephedraceae; Ephedra sinica Stapf) 9 g, Radix Glycyrrhizae (Glycyrrhiza uralensis Fisch; Fabaceae)6 g, Armeniacae Semen Amarum (Rosaceae; Prunus armeniaca L.var.ansu Maxim.) 9 g, Gypsum Fibrosum (CaSO4·2H2O) 15 ～ 30 g, Cinnamomi Ramulus (Lauraceae; Cinnamomum cassia Presl) 9 g, Alismatis Rhizoma (Alismataceae; Alisma orientale（Sam.）Juzep.) 9 g, Polyporus (Polyporaceae; Polyporus umbellatus（Pers.） Fries) 9 g, Atractylodis Macrocephalae Rhizoma (Asteraceae; Atractylodes macrocephala Koidz.) 9 g, Poria (Polyporaceae; Poria cocos（Schw.）Wolf) 15g, Radix Bupleuri (Apiaceae; Bupleurum chinense DC)16 g, Scutellariae Radix (Lamiaceae; Scutellaria baicalensis Georgi) 6 g, Pinelliae Rhizoma Praeparatum cum Zingibere et Alumine (Araceae; Pinellia ternata (Thunb.) Makino) 9 g, Rhizoma Zingiberis Recens (Zingiberaceae; Rhizoma Zingiberis Recens) 9 g，Asteris Radix et Rhizoma (Asteraceae; Aster tataricus L. f.) 9 g, Farfarae Flos (Asteraceae; Tussilago farfara L.) 9 g, Belamcandae Rhizoma (Iridaceae; Belamcanda chinensis（L.）DC) 9 g, Asari Radix et Rhizoma (Aristolochiaceae; Asarum heterotropoides Fr. Schmidt var. mandshuricum (Maxim.)Kitag) 6 g, Dioscoreae Rhizoma (Dioscoreaceae; Dioscorea opposita Thunb.) 12 g, Aurantii Fructus Immaturus (Rutaceae; Citrus aurantium L.) 6 g, Pericarpium Aurantii Nobilis (Rutaceae; Citrus × aurantium L) 6 g, Pogostemonis Herba (Lamiaceae; Pogostemon cablin（Blanco）Benth.) 9g. |
| Qinhai Ma, 2021  Xiaolong Xu, 2021 | Reduning injection, RDN | Preparation and Usage: obtained in the form of injection, intravenous drip; 20ml (10ml/piece) at a time.  Main components: Lonicerae Japonicae Flos (Caprifoliaceae; Lonicera japonica Thunb.), Artemisiae Annuae Herba (Asteraceae; Artemisia annua L.), and Gardeniae Fructus (Rubiaceae; Gardenia jasminoides Ellis) |
| Shuang Zhou, 2021 | Shenhuang granules, SH | Preparation and Usage: obtained in the form of hard granules, two times a day.  Main components: 50 g of Radix Ginseng (Araliaceae; Panax ginseng C.A.Mey), 40 g of Radix et Rhizoma Rhei (Polygonaceae; Rheum palmatum L.), 30g of Sargent gloryvine (Arecaceae; Daemonorops margaritae(Hance)Becc.), 30 g of Taraxaci Herba (Asteraceae; Taraxacum mongolicum Hand. -Mazz.), 50 g of Aconiti Lateralis Radix Praeparata (Ranunculaceae; Aconitum carmichaelii Debx.) and 6g of Hirudo (Hirudinidae; Whitmania pigra Whitman). |
| Li Ni, 2021 | Shuanghuanglian oral liquids, SHL | Preparation and Usage: obtained in the form of mixed liquid, 20ml at a time, three times a day. (China Pharmacopeia 2020: https://db.ouryao.com/yd2020/view.php?id=f97d134b2e)  Main components: Lonicerae Japonicae Flos (Caprifoliaceae; Lonicera japonica Thunb.), Astragali radix (Fabaceae; Astragalus membranaceus（Fisch.） Bge.var.mongholicus（Bge.）Hsiao), and Forsythiae Fructus (Oleaceae; Forsythia suspensa （Thunb.） Vahl). |
| Lulu Li, 2021 | Shenling Baizhu Powder + Yiqi Huoxue Powder, SLBZ +YQHX | Preparation and Usage: decocted in water/hard granules, one dose a day, three time a day.  Main components: Radix Ginseng (Araliaceae; Panax ginseng C.A.Mey) 15 g, Poria (Polyporaceae; Poria cocos（Schw.）Wolf) 10 g, Atractylodis Macrocephalae Rhizoma (Asteraceae; Atractylodes macrocephala Koidz.) 10 g, Lablab Semen Album (Fabaceae; Dolichos lablab L.) 15 g, Pericarpium Aurantii Nobilis (Rutaceae; Citrus × aurantium L) 10 g, Nelumbinis Semen (Nelumbonaceae; Nelumbo nucifera Gaertn.) 10 g, Dioscoreae Rhizoma (Dioscoreaceae; Dioscorea opposita Thunb.) 15 g, Coicis Semen (Poaceae; Coix lacryma-jobi L.var.ma-yuen(Roman.) Stapf) 15 g, Amomi Fructus (Zingiberaceae; Amomum villosum Lour.) 5 g, Radix Platycodonis (Campanulaceae; Platycodon grandiflorum （Jacq.）A.DC.) 10 g, Salviae Miltiorrhizae Radix et Rhizoma (Lamiaceae; Salvia miltiorrhiza Bge.) 15g, Pheretima (Megascolecidae; Pheretima aspergillum(E.Perrier)) 10 g, Astragali radix (Fabaceae; Astragalus membranaceus（Fisch.） Bge.var.mongholicus（Bge.）Hsiao) 15 g, Angelicae Sinensis Radix(Apiaceae; Angelica sinensis（Oliv.）Diels) 10 g, Chuanxiong Rhizoma (Apiaceae; Ligusticum chuanxiong Hort.) 10 g, Jianqu (Medicated Leaven) 10g. |
| Li, L., 2021 | Shumian capsule, SM | Preparation and Usage: obtained in the form of capsules, 1.2g/3 capsules at a time, two times a day.  Main components: Ziziphi spinosae Semen (Rhamnaceae; Ziziphus jujuba Mill. var. spinosa（Bunge）Hu ex H. F. Chou), Radix Bupleuri (Apiaceae; Bupleurum chinense DC), Paeoniae Radix Alba (Paeoniaceae; Paeonia lactiflora Pall.), Albiziae Flos (Fabaceae; Albizia julibrissin Durazz.), Albiziae Cortex (Fabaceae; Albizia julibrissin Durazz.), Bombyx Batryticatus (Bombycidae; Bombyx mori Linnaeus), Cicadae Periostracum (Cicadidae; Cryptotympana pustulata Fabricius), Junci Medulla (Juncaceae; Juncus effusus L.). |
| Qin He, 2020 | Shengmai Powder, SMP | Preparation and Usage: decocted in water, one dose a day.  Main components: Codonopsis Radix (Campanulaceae; Codonopsis pilosula (Franch.)Nannf.), Ophiopogonis Radix (Asparagaceae; Ophiopogon japonicus (L.f) Ker-Gawl.), Schisandrae Chinensis Fructus (Schisandraceae; Schisandra chinensis（Turcz.）Baill.), Curcumae Radix (Zingiberaceae; Curcuma wenyujin Y. H. Chen et C. Ling), Cyperi Rhizoma (Cyperaceae; Cyperus rotundus L.), Angelicae Sinensis Radix (Apiaceae; Angelica sinensis（Oliv.）Diels), Gypsum Fibrosum (CaSO4·2H2O), Radix Glycyrrhizae (Glycyrrhiza uralensis Fisch; Fabaceae), Wheat Rice (Poaceae; Triticum aestivum L), Jujubae Fructus (Rhamnaceae; Ziziphus jujuba Mill.). |
| Lin Wang, 2020 | Shengmai Powder + Shenling Baizhu Powder , SMP + SLBZ | Preparation and Usage: decocted in water, one dose a day.  Main components: Pseudostellariae Radix (Caryophyllaceae; Pseudostellaria heterophylla（Miq.）Pax ex Pax et Hoffm.) 30g，Ophiopogonis Radix (Asparagaceae; Ophiopogon japonicus (L.f) Ker-Gawl.)15g, Schisandrae Chinensis Fructus (Schisandraceae; Schisandra chinensis（Turcz.）Baill.) 15g, Poria (Polyporaceae; Poria cocos（Schw.）Wolf) 15g, Atractylodis Macrocephalae Rhizoma (Asteraceae; Atractylodes macrocephala Koidz.) 15g，Lablab Semen Album (Fabaceae; Dolichos lablab L.) 15g，Pericarpium Aurantii Nobilis (Rutaceae; Citrus × aurantium L)10g, Dioscoreae Rhizoma (Dioscoreaceae; Dioscorea opposita Thunb.) 20g, Radix Platycodonis (Campanulaceae; Platycodon grandiflorum （Jacq.）A.DC.) 10g, Magnoliae Officinalis Cortex (Magnoliaceae; Magnolia officinalis Rehd.et Wils.) 10g, Rehmanniae Radix (Scrophulariaceae; Rehmannia glutinosa Libosch.) 15g, Scrophulariae Radix (Scrophulariaceae; Scrophularia ningpoensis Hemsl.) 10g, Salviae Miltiorrhizae Radix et Rhizoma (Lamiaceae; Salvia miltiorrhiza Bge.) 20g, Radix Glycyrrhizae (Glycyrrhiza uralensis Fisch; Fabaceae)10g. |
| Dandan Song, 2021 | Suanzaoren Decoction, SZR | Preparation and Usage: decocted in water, one dose a day, three time a day.  Main components: Ziziphi spinosae Semen (Rhamnaceae; Ziziphus jujuba Mill. var. spinosa（Bunge）Hu ex H. F. Chou), Radix Glycyrrhizae (Glycyrrhiza uralensis Fisch; Fabaceae), Anemarrhenae Rhizoma (Liliaceae; Anemarrhena asphodeloides Bge.), Poria (Polyporaceae; Poria cocos（Schw.）Wolf), Chuanxiong Rhizoma (Apiaceae; Ligusticum chuanxiong Hort.). |
| Xiangyong Yan, 2021 | Wenyang Huashi Prescription + Qingre Xuanfei Recipe, WYHS + QRXF | Preparation and Usage: decocted in water, one dose a day, two time a day.  Main components (WYHS): Rhizoma Atractylodis (Asteraceae; Atractylodes lancea (Thunb.) DC)12 g, Pericarpium Aurantii Nobilis (Rutaceae; Citrus × aurantium L)10 g, Magnoliae Officinalis Cortex (Magnoliaceae; Magnolia officinalis Rehd.et Wils.) 10 g,Pogostemonis Herba (Lamiaceae; Pogostemon cablin（Blanco）Benth.) 10 g, Tsaoko Fructus (Zingiberaceae; Amomum tsao-ko Crevost et Lemaire) 6 g, Ephedrae Herba (Ephedraceae; Ephedra sinica Stapf) 6 g, Notopterygii Rhizoma et Radix (Apiaceae; Notopterygium incisum Ting ex H. T. Chang) 10 g, Rhizoma Zingiberis Recens (Zingiberaceae; Rhizoma Zingiberis Recens) 6 g，Arecae Semen (Arecaceae; Areca catechu L.) 10 g, Belamcandae Rhizoma (Iridaceae; Belamcanda chinensis（L.）DC) 6 g, Forsythiae Fructus (Oleaceae; Forsythia suspensa （Thunb.） Vahl)6 g, Chuanxiong Rhizoma (Apiaceae; Ligusticum chuanxiong Hort.) 10 g, Trichosanthis Fructus (Cucurbitaceae; Trichosanthes kirilowii Maxim.)10 g.  Main components (QRXF): Ephedrae Herba (Ephedraceae; Ephedra sinica Stapf) 6 g, Armeniacae Semen Amarum (Rosaceae; Prunus armeniaca L.var.ansu Maxim.) 10 g, Gypsum Fibrosum (CaSO4·2H2O) 15 g, Coicis Semen (Poaceae; Coix lacryma-jobi L.var.ma-yuen(Roman.) Stapf) 30 g，Rhizoma Atractylodis (Asteraceae; Atractylodes lancea (Thunb.) DC)10 g, Pogostemonis Herba (Lamiaceae; Pogostemon cablin（Blanco）Benth.)10 g, Phragmitis Rhizoma (Poaceae; Phragmites communis Trin.) 15 g, Descurainiae Semen Lepidii Semen (Brassicaceae; Descurainia sophia（L.）Webb. ex Prantl.) 12 g, Citri Exocarpium Rubrum (Rutaceae; Citrus reticulata Blanco)10 g, Salviae Miltiorrhizae Radix et Rhizoma (Lamiaceae; Salvia miltiorrhiza Bge.)10 g, Luffae Fructus Retinervus (Cucurbitaceae; Luffa cylindrica（L.）Roem.)10 gTrichosanthis Pericarpium (Cucurbitaceae; Trichosanthes kirilowii Maxim.) 10 g, Scutellariae Radix (Lamiaceae; Scutellaria baicalensis Georgi) 6 g, Mori Cortex (Moraceae; Morus alba L.) 10 g, Bambusae Caulis In Taenias (Poaceae; Bambusa tuldoides Munro)10 g. |
| liangzhong chen, 2020  Long Wen, 2020  Kaifeng Liu, 2020  Zhijian Luo, 2021 | Xuebijing injection, XBJ | Preparation and Usage: obtained in the form of injection, intravenous drip; 50ml at a time.  Main components: Paeoniae Radix Rubra (Ranunculaceae; Paeonia lactiflora Pall.), Angelicae Sinensis Radix (Apiaceae; Angelica sinensis（Oliv.）Diels), Chuanxiong Rhizoma (Apiaceae; Ligusticum chuanxiong Hort.), Carthami Flos (Asteraceae; Carthamus tinctorius L.) and Salviae Miltiorrhizae Radix et Rhizoma (Lamiaceae; Salvia miltiorrhiza Bge.). |
| Zizhou Zheng, 2020 | Xiaochaihu Decoction + Maxing Shigan Decoction, XCH + MXSG | Preparation and Usage: decocted in water, one dose a day, three time a day.  Main components: Radix Bupleuri (Apiaceae; Bupleurum chinense DC) 20 g, Scutellariae Radix (Lamiaceae; Scutellaria baicalensis Georgi) 12 g, Pinelliae Rhizoma Praeparatum (Araceae; Pinellia ternata (Thunb.) Makino) 12 g, Codonopsis Radix (Campanulaceae; Codonopsis pilosula (Franch.)Nannf.), 15 g, Zingiberis Rhizoma (Zingiberaceae; Zingiber officinale Rosc.) 10 g, Jujubae Fructus (Rhamnaceae; Ziziphus jujuba Mill.) 12 g, Radix Glycyrrhizae (Fabaceae; Glycyrrhiza uralensis Fisch) 10 g, Ephedrae Herba (Ephedraceae; Ephedra sinica Stapf) 10 g, Armeniacae Semen Amarum (Rosaceae; Prunus armeniaca L.var.ansu Maxim.) 12 g, Gypsum Fibrosum (CaSO4·2H2O) 30 g, Phragmitis Rhizoma (Poaceae; Phragmites communis Trin.) 30 g, Asteris Radix et Rhizoma (Asteraceae; Aster tataricus L. f.) 15 g, Farfarae Flos (Asteraceae; Tussilago farfara L.) 15 g, Cicadae Periostracum (Cicadidae; Cryptotympana pustulata Fabricius) 10 g, Coicis Semen (Poaceae; Coix lacryma-jobi L.var.ma-yuen(Roman.) Stapf) 20 g, Fructus Hordei Germinatus (Poaceae; Hordeum vulgare L.) 20 g |
| Wuzhong Xiong, 2020 | Xuanfei Baidu Decoction, XFBD | Preparation and Usage: decocted in water, one dose a day, two time a day.  Main components: Ephedrae Herba (Ephedraceae; Ephedra sinica Stapf) 8 g, Armeniacae Semen Amarum (Rosaceae; Prunus armeniaca L.var.ansu Maxim.) 15 g, Gypsum Fibrosum (CaSO4·2H2O) 30 g, Rhizoma Atractylodis (Asteraceae; Atractylodes lancea (Thunb.) DC) 10 g, Coicis Semen (Poaceae; Coix lacryma-jobi L.var.ma-yuen(Roman.) Stapf) 30 g, Pogostemonis Herba (Lamiaceae; Pogostemon cablin（Blanco）Benth.)15 g, Holothuria pervicax (Polygonaceae; Reynoutria japonica Houtt) 20 g, Descurainiae Semen Lepidii Semen (Brassicaceae; Descurainia sophia（L.）Webb. ex Prantl.) 15 g, Verbenae Herba (Verbenaceae; Verbena officinalis L.) 30 g, Phragmitis Rhizoma (Poaceae; Phragmites communis Trin.) 30 g, Artemisiae Annuae Herba (Asteraceae; Artemisia annua L.) 25 g, Citri Exocarpium Rubrum (Rutaceae; Citrus reticulata Blanco) 20 g, Radix Glycyrrhizae (Fabaceae; Glycyrrhiza uralensis Fisch) 10 g. |
| Feng Zhao, 2020 | Xuanfei Dayu Decoction, XFDY | Preparation and Usage: decocted in water, one dose a day, two time a day.  Main components: Codonopsis Radix (Campanulaceae; Codonopsis pilosula (Franch.)Nannf.) 15g, Poria (Polyporaceae; Poria cocos（Schw.）Wolf)15g,、Pericarpium Aurantii Nobilis (Rutaceae; Citrus × aurantium L) 15g, , Pinelliae Rhizoma Praeparatum (Araceae; Pinellia ternata (Thunb.) Makino) 10g, Armeniacae Semen Amarum (Rosaceae; Prunus armeniaca L.var.ansu Maxim.) 10g, Paeoniae Radix Alba (Paeoniaceae; Paeonia lactiflora Pall.) 10g, Cinnamomi Ramulus (Lauraceae; Cinnamomum cassia Presl) 10g, Trionycis Carapax (Trionychidae; Trionyx sinensis Wiegmann)12gZingiberis Rhizoma (Zingiberaceae; Zingiber officinale Rosc.) 8g, Lilii Bulbus (Liliaceae; Lilium lancifolium Thunb.) 20g, Radix Glycyrrhizae (Glycyrrhiza uralensis Fisch; Fabaceae) 6g. |
| Xinyi Zhang, 2021 | Xiyanping injection, XYP | Preparation and Usage: obtained in the form of injection, intramuscular injection; 50-100mg once, 2-3 times a day.  Main components: Andrographis paniculate |
| Yusheng Yan, 2021 | Yinghuang Qingfei Capsule, YHQF | Preparation and Usage: obtained in the form of capsules, 0.45g/3 capsules at a time, three times a day. (China Pharmacopeia 2020: https://db.ouryao.com/yd2020/view.php?id=fc7db7ef91)  Main components: Descurainiae Semen Lepidii Semen (Brassicaceae; Descurainia sophia（L.）Webb. ex Prantl.), Ephedrae Herba (Ephedraceae; Ephedra sinica Stapf), Armeniacae Semen Amarum (Rosaceae; Prunus armeniaca L.var.ansu Maxim.), Bulbus Fritillariae Thunbergii (Liliaceae; Fritillaria thunbergii Miq.), Folium Eriobotryae (Rosaceae; Eriobotrya japonica（Thunb.）Lindl.), Isatidis Folium (Brassicaceae; Isatis indigotica Fort.), Acori Tatarinowii Rhizoma (Araceae; Acorus tatarinowii Schott), Dioscoreae Nipponicae Rhizoma (Dioscoreaceae; Dioscorea nipponica Makino), Artemisiae Annuae Herba (Asteraceae; Artemisia annua L.), Ginkgo Folium (Ginkgoaceae; Ginkgo biloba L.), Schisandrae Chinensis Fructus (Schisandraceae; Schisandra chinensis (Turcz.)Baill.), Aurantii Fructus Immaturus (Rutaceae; Citrus aurantium L.), Gypsum Fibrosum (CaSO4·2H2O), Radix Glycyrrhizae (Fabaceae; Glycyrrhiza uralensis Fisch). |
| Suofang Shi, 2020 | Yiqi Yangyin Granule +Taiji Liuqi method, YQYY + TJLQ | Preparation and Usage: obtained in the form of granule, 2 packs at a time, two times a day.  Main components: Codonopsis Radix (Campanulaceae; Codonopsis pilosula (Franch.) Nannf.), Ophiopogonis Radix (Asparagaceae; Ophiopogon japonicus (L.f) Ker-Gawl.), Lilii Bulbus (Liliaceae; Lilium lancifolium Thunb.), Poria (Polyporaceae; Poria cocos（Schw.）Wolf), Atractylodis Macrocephalae Rhizoma (Asteraceae; Atractylodes macrocephala Koidz.), Pericarpium Aurantii Nobilis (Rutaceae; Citrus aurantium L), Fructus Hordei Germinatus (Poaceae; Hordeum vulgare L.), Albiziae Cortex (Fabaceae; Albizia julibrissin Durazz.), Lycii Cortex (Solanaceae; Lycium chinense Mill.), Radix Glycyrrhizae (Glycyrrhiza uralensis Fisch; Fabaceae). |
| Xiaolong Chen, 2020 | Zhenqi Fuzheng capsule, ZQFZ | Preparation and Usage: obtained in the form of capsules, 1.4g/4 capsules at a time, two times a day.  Main components: Ligustri Lucidi Fructus (Oleaceae; Ligustrum lucidum Ait.), Astragali radix (Fabaceae; Astragalus membranaceus（Fisch.） Bge.var.mongholicus（Bge.）Hsiao). |
